# Supplementary material for: Global analysis of cancer cell responses to USP9X inhibition
Source: EMBO J. 2026 Apr 7;45(9):3306–31. doi: 10.1038/s44318-026-00742-y (PMC13144739; doi:10.1038/s44318-026-00742-y)
Supplement: Supplementary file 1 — Appendix [file 44318_2026_742_MOESM1_ESM.pdf]

Appendix for

Global analysis of cancer cell responses to USP9X inhibition

Philipp Schenk *et al*.

Corresponding author: David Komander, dk@wehi.edu.au

Table of Contents

Appendix Figures S1 to S9 including Legends 2

Appendix Table S1 24

Appendix Material and Methods 25

Appendix References 30

Appendix Figure S1

A

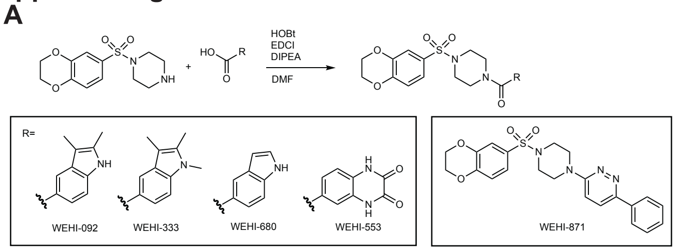

B

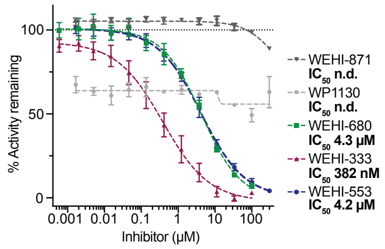

D

Compound	Steady State Affinity (K_D) [µM]	IC_{50} [µM]
FT709	0.069 ± 0.002 (N=3)	0.124 (N=4)
**WEHI-092**	**1.05 ± 0.20 (N=3)**	**0.254 (N=4)**
WEHI-333	1.05 ± 0.26 (N=3)	0.382 (N=3)
WEHI-680	3.51 ± 0.58 (N=3)	4.3 (N=3)
WEHI-871	n.d. (N=3)	n.d. (N=3)
WEHI-553	Not tested	4.2 (N=6)

C

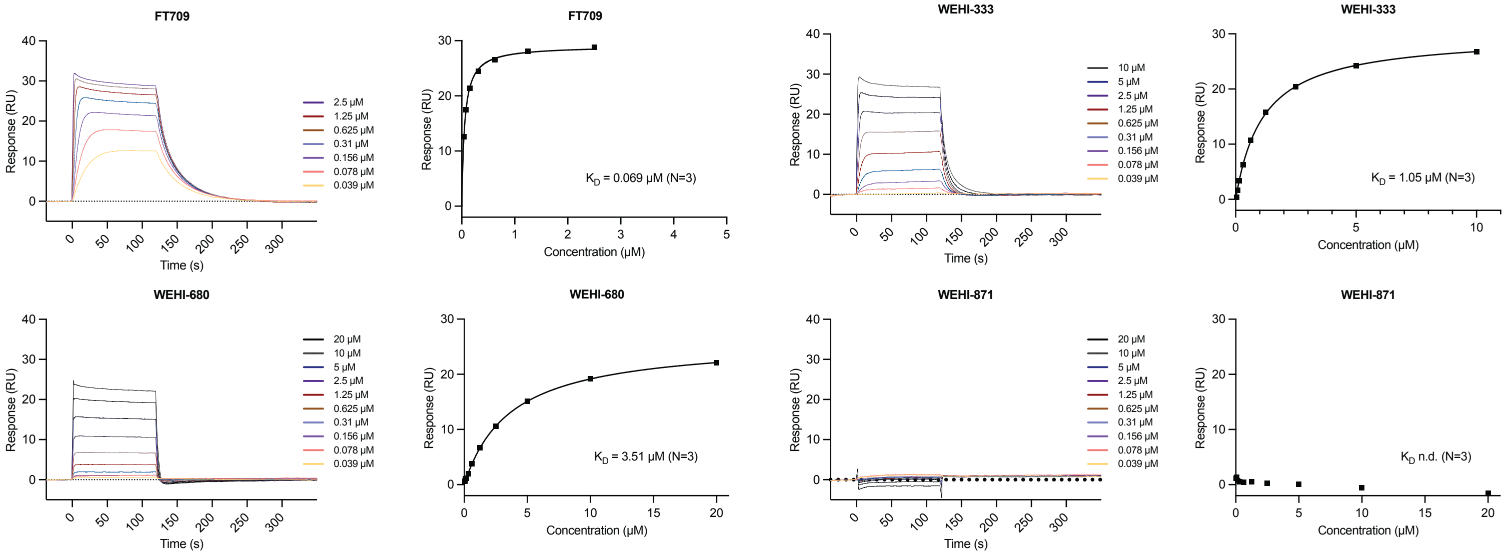

E

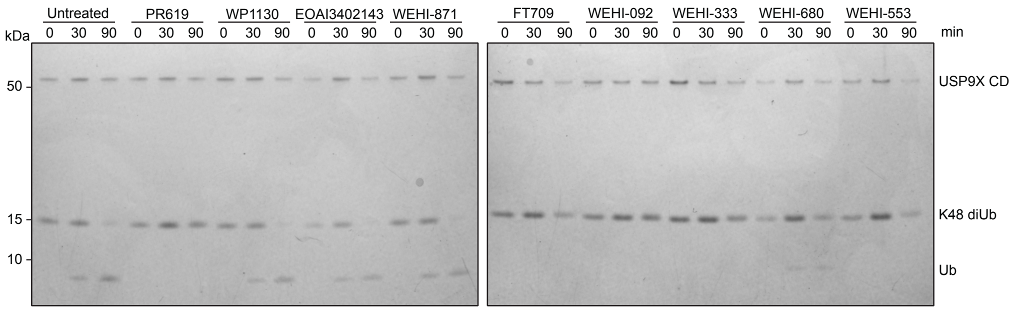

F

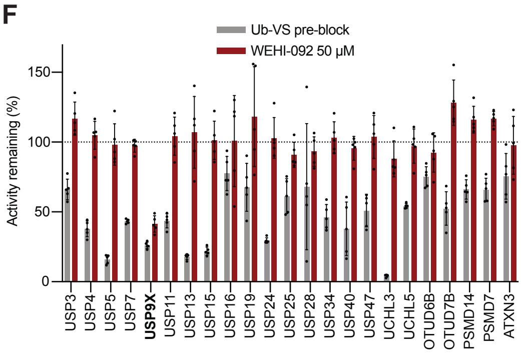

Appendix Fig S1. WEHI series USP9X inhibitors chemistry and assessment.

(**A**) Chemical synthesis of WEHI series USP9X compounds and control WEHI-871. See **Appendix Material and Methods: Medicinal chemistry** for further details. (**B**) Ub-Rho cleavage assay to assess inhibitor potency against recombinant USP9X catalytic domain. Data shown is the mean of three independent biological repeats with two technical replicates per experiment. WP1130 data is the mean of two independent biological repeats. A nonlinear curve fit generated using GraphPad Prism (v10.3) was used for calculating IC_{50} values. n.d. - not determined; error bars, s.e.m. (**C**) SPR binding assay for USP9X inhibitors to assess binding to the USP9X catalytic domain. For each compound: *Left*, fitted binding curve; *Right*, raw sensorgram data. K_D shown is the mean of three biological repeats. Representative graphs from one experiment are shown. (**D**) Overview table of K_D (determined via SPR) and IC_{50} (determined via Ub-Rho assay) for compounds tested in this study (n.d. - not determined). (**E**) Coomassie gel of a di-ubiquitin (diUb) cleavage assay using K48-linked diUb with 150 µM compound. Representative gel of two independent biological repeats. (**F**) DUB IP-MS experiment to assess maximum level of inhibition with Ub-VS probe. Pre-treatment of MCF-7 cell lysates with 0.5 µg Ub-VS was performed for 20 min at ambient temperature. Data shown is the mean of four or five biological replicates per condition which are shown as individual datapoints; error bars, s.d.

Appendix Figure S2

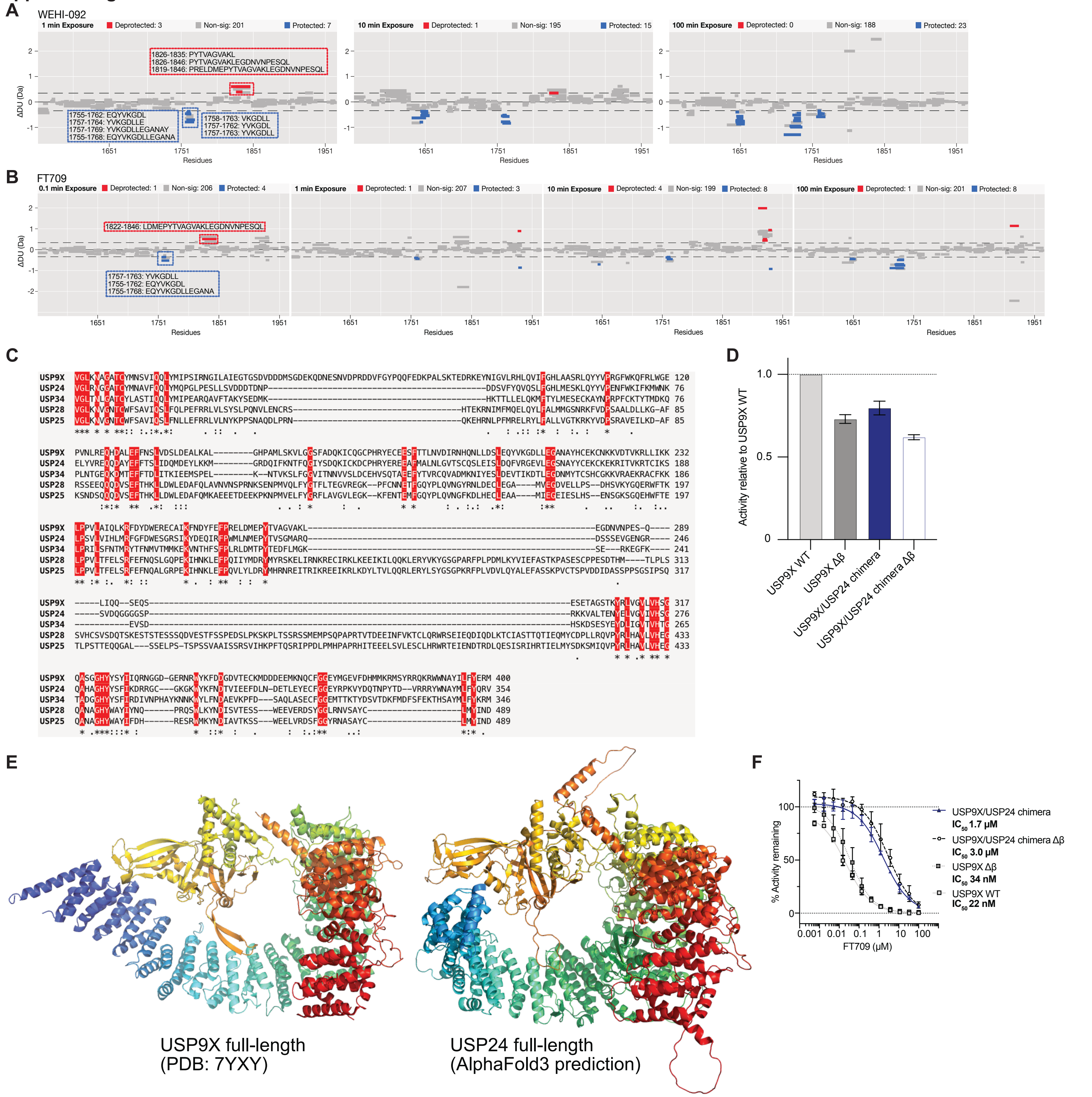

Appendix Fig S2. HDX-MS and USP9X catalytic domain mutants.

(**A**) Hybrid Woods differential plot highlighting significant ($P < 0.01$) peptide differences in hydrogen-deuterium uptake between USP9X catalytic domain apo and WEHI-092 bound protein protected peptides in blue and deprotected peptides in red. The dashed lines indicate the confidence limit at 99% (hybrid significance test, significance confirmed by a Welch's t test). Plots were generated using Deuteros software (v2.0). (**B**) As in **A** and **Fig 2A**, but for FT709 with similar peptide motifs detected as for WEHI-092 binding. (**C**) Sequence alignment of the indicated human USP DUB catalytic domains. (**D**) Catalytic activities of indicated wild-type (WT) and mutant USP9X proteins, assessed by Ub-Rho cleavage assays. Data shown is the mean of three independent biological repeats with four technical replicates per experiment. Error bars, s.e.m. (**E**) Side by side comparison of full-length USP9X (PDB: 7YXY) and full-length USP24 (AlphaFold3 prediction, residues 1-400 removed for visualisation purposes). (**F**) FT709 potency against recombinant USP9X catalytic domain variants: USP9X wild-type (WT), USP9X/USP24 chimera (USP9X with five USP24-like mutations in compound binding region), USP9X $\Delta\beta$ (USP9X β-hairpin deletion) and USP9X/USP24 chimera $\Delta\beta$ (USP9X $\Delta\beta$ with five USP24-like mutations) determined from Ub-Rho cleavage assay. Data shown is the mean of two independent biological repeats with two technical replicates per experiment. Curve shown is the nonlinear curve fit generated using GraphPad Prism (v10.3) and was used for calculating the IC_{50} values. Error bars, s.e.m.

Appendix Figure S3

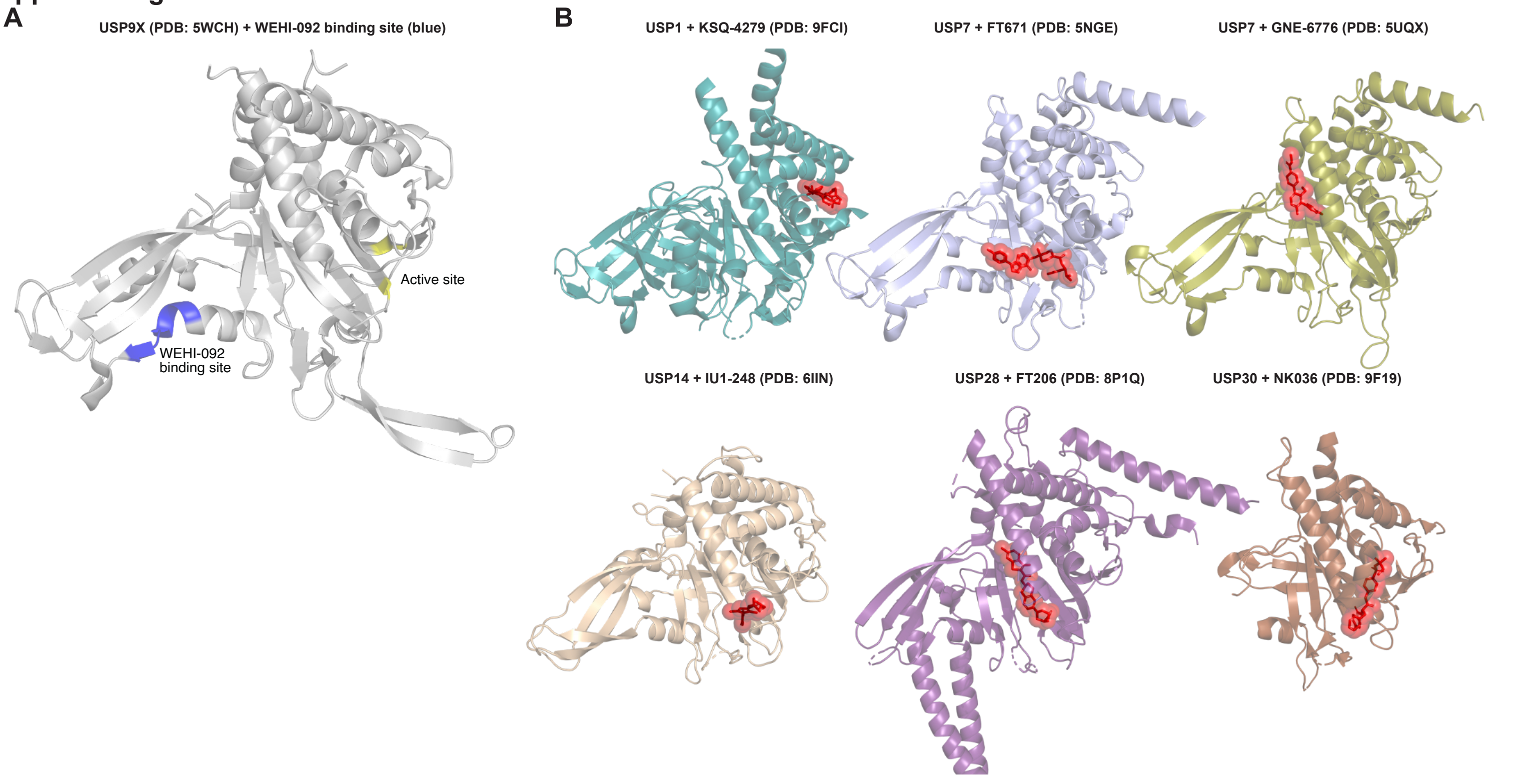

Appendix Fig S3. Comparison of binding sites of selective USP DUB inhibitors.

(**A**) USP9X catalytic domain; PDB: 5WCH (Paudel *et al*, 2019) with the WEHI-092 binding site (6 aa motif: YVKGDL) highlighted in blue and the active site highlighted in yellow. (**B**) Side by side comparison of co-crystal structures of USP DUBs and their selective inhibitors shown in red. The following co-crystal structures are visualised: USP1 in complex with KSQ-4279; PDB: 9FCI (Cadzow *et al*, 2024; Rennie *et al*, 2024), USP7 in complex with FT671; PDB: 5NGE (Turnbull *et al*, 2017), USP7 in complex with GNE-6776; PDB: 5UQX (Kategaya *et al*, 2017), USP14 in complex with IU1-248; PDB: 6IIN (Wang *et al*, 2018), USP28 in complex with FT206; PDB: 8P1Q (Ruiz *et al*, 2021; Patzke *et al*, 2024), USP30 in complex with NK036; PDB: 9F19 (Kazi *et al*, 2025).

Appendix Figure S4

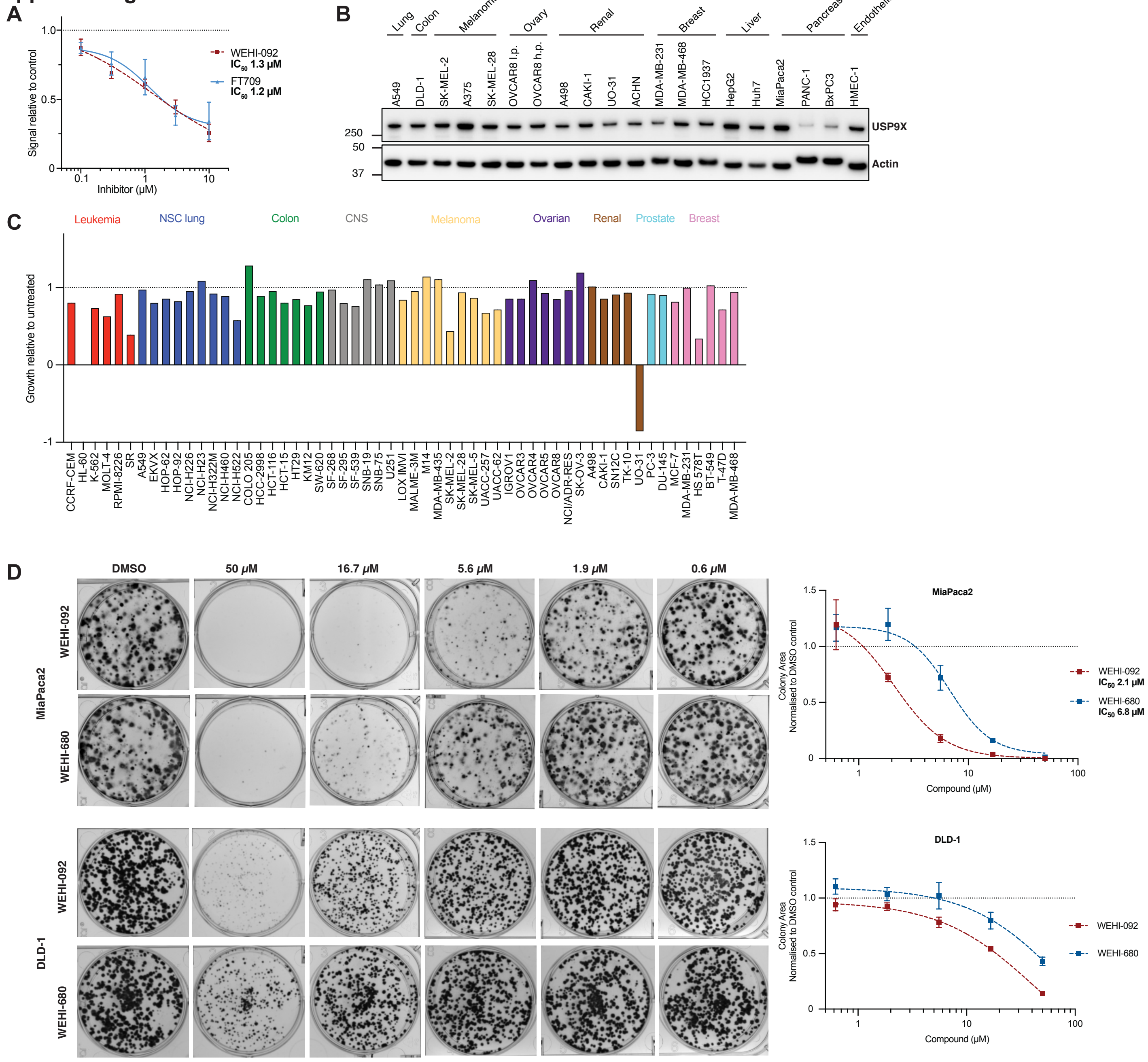

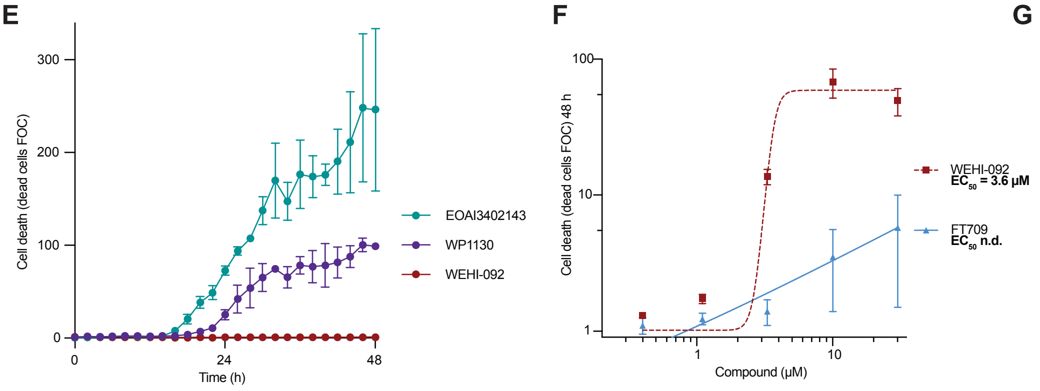

G

Compound	Mass balance (%)		P_{app} (10^{-6} cm/s)		Efflux ratio
	A-B	B-A	A-B	B-A	
WEHI-092	90	91	42.6	70.4	1.7
FT709	98	90	3.8	69.4	18

H

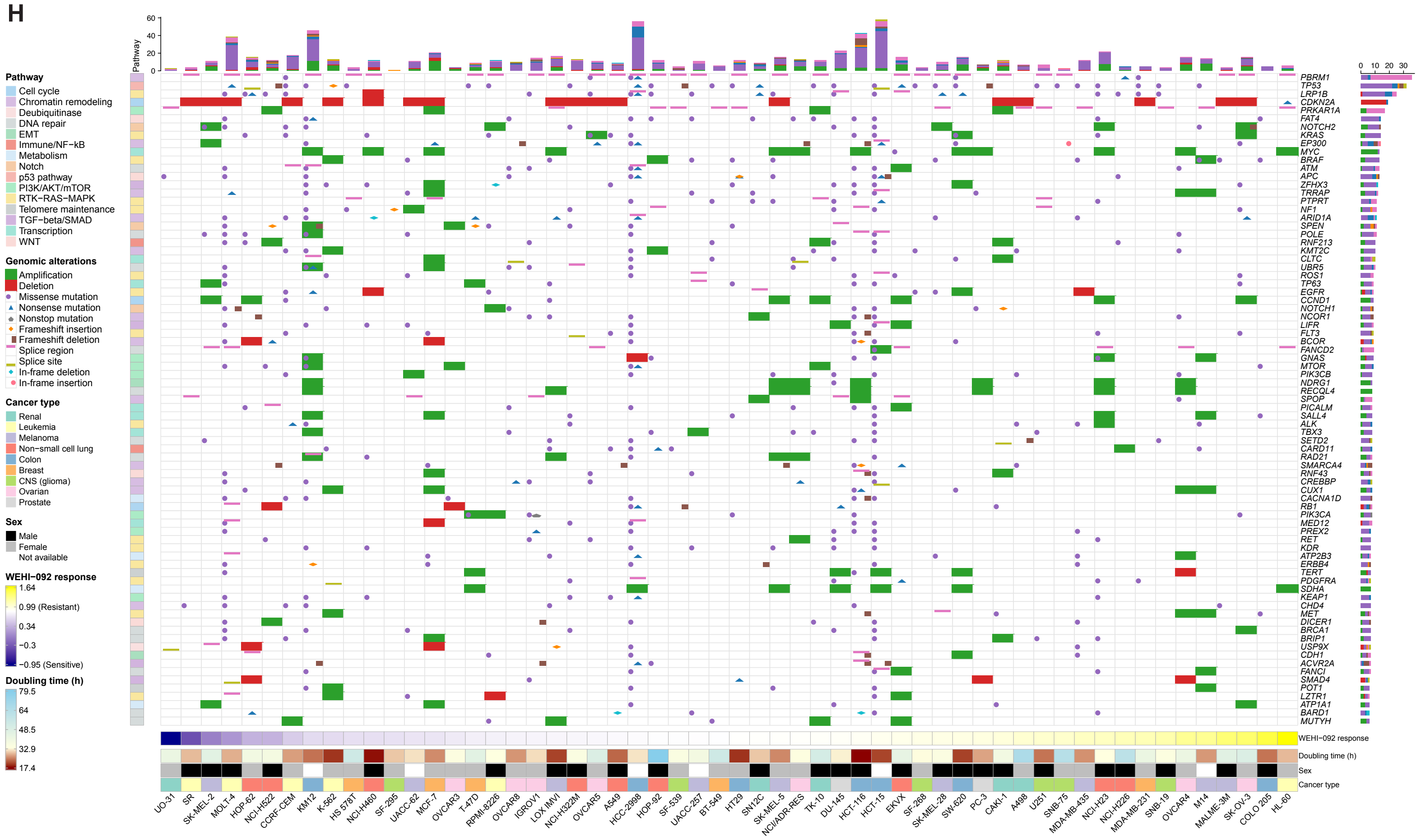

Pathway
60
40
20
0
0 10 20 30
Pathway
Cell cycle
Chromatin remodeling
Deubiquitinase
DNA repair
EMT
Immune/NF-kB
Metabolism
Notch
p53 pathway
PI3K/AKT/mTOR
RTK−RAS−MAPK
Telomere maintenance
TGF−beta/SMAD
Transcription
WNT
Genomic alterations
Amplification
Deletion
Missense mutation
Nonsense mutation
Nonstop mutation
Frameshift insertion
Frameshift deletion
Splice region
Splice site
In-frame deletion
In-frame insertion
Cancer type
Renal
Leukemia
Melanoma
Non-small cell lung
Colon
Breast
CNS (glioma)
Ovarian
Prostate
Sex
Male
Female
Not available
WEHI−092 response
1.64
0.99 (Resistant)
0.34
−0.3
−0.95 (Sensitive)
Doubling time (h)
79.5
64
48.5
32.9
17.4
PBRM1
TP53
LRP1B
CDKN2A
PRKAR1A
FAT4
NOTCH2
KRAS
EP300
MYC
BRAF
ATM
APC
ZFHX3
TRRAP
PTPRT
NF1
ARID1A
SPEN
POLE
RNF213
KMT2C
CLTC
UBR5
ROS1
TP63
EGFR
CCND1
NOTCH1
NCOR1
LIFR
FLT3
BCOR
FANCD2
GNAS
MTOR
PIK3CB
NDRG1
RECQL4
SPOP
PICALM
SALL4
ALK
TBX3
SETD2
CARD11
RAD21
SMARCA4
RNF43
CREBBP
CUX1
CACNA1D
RB1
PIK3CA
MED12
PREX2
RET
KDR
ATP2B3
ERBB4
TERT
PDGFRA
SDHA
KEAP1
CHD4
MET
DICER1
BRCA1
BRIP1
USP9X
CDH1
ACVR2A
FANCI
SMAD4
POT1
LZTR1
ATP1A1
BARD1
MUTYH
WEHI−092 response
Doubling time (h)
Sex
Cancer type
UO-31
SR
SK-MEL-2
MOLT-4
HOP-62
NCI-H522
CCRF-CEM
KM12
K-562
HS 578T
NCI-H460
SF-295
UACC-62
MCF-7
OVCAR3
T-47D
RPMI-8226
OVCAR8
IGROV1
LOX IMVI
NCI-H322M
OVCAR5
A549
HCC-2998
HOP-92
SF-539
UACC-257
BT-549
HT29
SN12C
SK-MEL-5
NCI/ADR-RES
TK-10
DU-145
HCT-116
HCT-15
EKVX
SF-268
SK-MEL-28
SW-620
PC-3
CAKI-1
A498
U251
SNB-75
MDA-MB-435
NCI-H23
NCI-H226
MDA-MB-231
SNB-19
OVCAR4
M14
MALME-3M
SK-OV-3
COLO 205
HL-60

Appendix Fig S4. WEHI-092 phenotypes across cell lines.

(**A**) IC_{50} of USP9X inhibitors in MDA-MB-231 cells measured via degradation of the USP9X substrate CEP55 by Western Blot. The compounds were incubated with cells for 24 h. Data shown is the mean of three (WEHI-092) and two (FT709) independent biological repeats. Error bars, s.e.m. (**B**) Western Blot analysis of USP9X expression levels across a subset of cell lines used in this study. Representative blot from two independent biological repeats is shown with Actin as a sample loading control. l.p. - low passage; h.p. - high passage. (**C**) NCI-60 cancer cell panel of 57 cancer cell lines treated with 10 µM WEHI-680 for 3 days. Data shown is the cell growth at 3 days post treatment relative to untreated control and relative to day 0 for each individual cell line. Negative values indicate cell death at 3 days post treatment. Cancer cell lines are colour-coded depending on the cancer type. NCS - non-small cell. CNS - central nervous system. Data shown is the mean of two technical replicates. (**D**) WEHI-092 and WEHI-680 titration of clonogenic potential assays for MiaPaca2 and DLD-1 cells. *Left,* representative images from three independent biological repeats with three technical replicates per experiment. *Right,* quantification of images plotted as dose-dependent colony area. Curve shown is the nonlinear curve fit generated using GraphPad Prism (v10.3) and was used for calculating the IC_{50} values. Error bars, s.e.m. (**E**) IncuCyte live-cell imaging data for primary human dermal fibroblast cells. Data points shown are the mean of two independent biological repeats with two technical replicates per experiment. FOC - fold over control. EOAI3402143, WP1130 and WEHI-092 were used at 50 µM. Error bars, s.e.m. (**F**) IncuCyte live-cell imaging data showing the effects of WEHI-092 and FT709 on cell killing of UO-31 cells. Data points shown are the mean of three independent biological

repeats with two technical replicates per experiment. Curve shown is the nonlinear curve fit generated using GraphPad Prism (v10.3) and was used for calculating the EC_{50} (n.d. - not determined). FOC - fold over control. Error bars, s.e.m. **(G)** Caco-2 cell permeability assessment of WEHI-092 and FT709. Values are presented as the average of two biological replicates. P_{app} - apparent permeability. **(H)** Oncoprint for NCI-60 cell line panel. Genes included in the oncoprint were those listed in the COSMIC Hallmarks of Cancer Genes dataset (v103, GRCh37) with an alteration frequency greater than 10% and *USP9X* was additionally included. Bar plot on top shows % of mutated genes per cell line (colour-coded by genomic alteration type). Bar plot on the right shows the frequency of the type of alteration (colour-coded by genomic alteration type). Each gene was assigned a pathway annotation. MDA-MB-468 cell line was excluded from the oncoprint as no data was available for this cell line. WEHI-092 response refers to NCI-60 panel testing (data from **Fig 3B**).

Appendix Figure S5

A

	**Steger et al, 2021 *Nat Commun*** HCT116 cells	**This study** DLD-1 cells
	**30 min FT671 10 µM**	
# total peptides detected	45,821	40,802
	**23,568 peptides identified in both studies**	
# K-GG peptides, 2-fold increase, adj. *p*-value <0.05	1,195	1,030
	**643 K-GG peptides identified in both studies 53.8% overlap**	

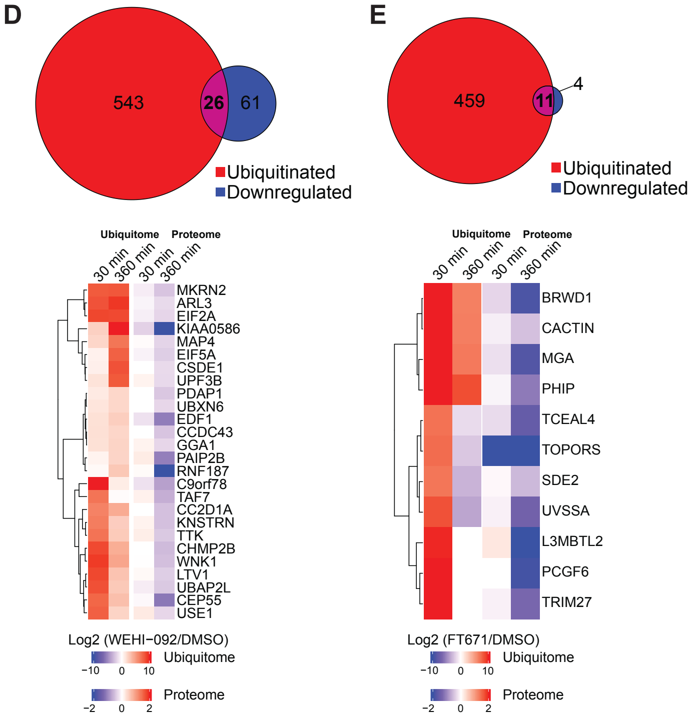

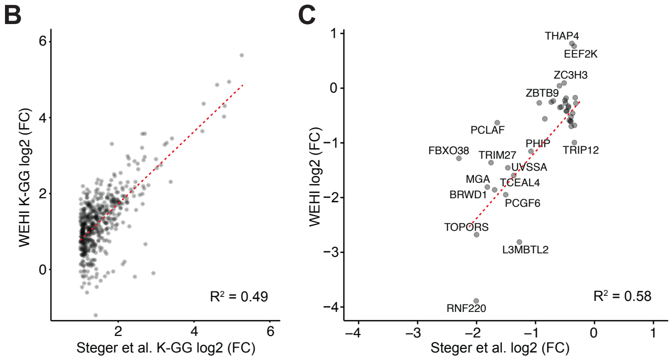

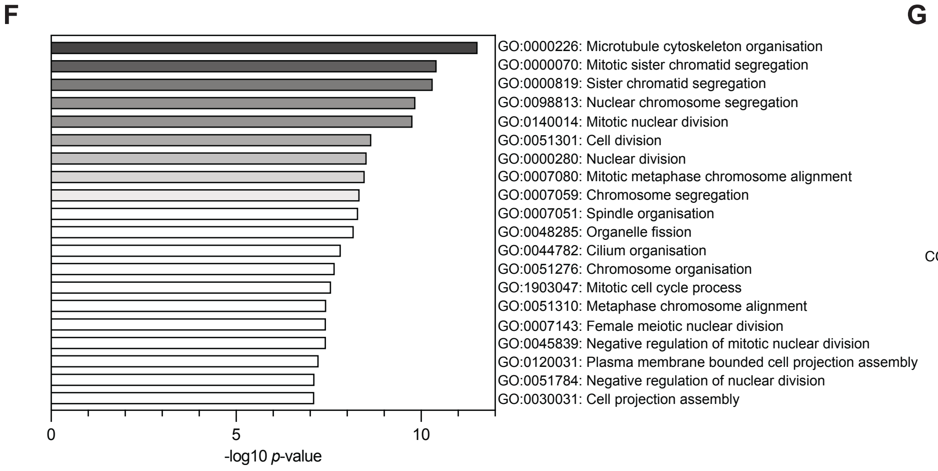

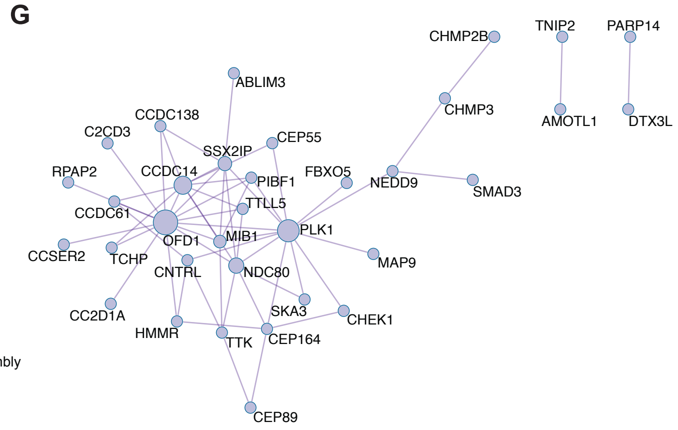

Appendix Fig S5. USP7i and USP9Xi ubiquitinomics data.

(**A**) Comparison of data output numbers of ubiquitinomics experiments benchmarking our study against the study on USP7 inhibition by Steger and colleagues (Steger *et al*, 2021) using the USP7 inhibitor FT671 on colorectal carcinoma cell lines. (**B**) Comparison between significantly increased K-GG peptides at 30 min between the study on USP7 inhibition by Steger and colleagues (Steger *et al*, 2021; filtered for log2 > onefold change FT671 over untreated control, adjusted *P* value < 0.05) and our study (adjusted *P* value < 0.05). The data was pre-filtered allowing a missingness of zero. (**C**) Comparison between proteins downregulated (log2 < -0.3-fold change FT671 over untreated control, adjusted *P* value < 0.05) in the study on USP7 inhibition by Steger and colleagues (Steger *et al*, 2021) and our study. BPCA+MinD was used for imputation. (**D**) DLD-1 time-resolved proteome and ubiquitome profiling upon WEHI-092 treatment (10 μM) for 30 min and 360 min. The data was filtered for increased ubiquitination (log2 > twofold change over untreated control, adjusted *P* value < 0.05) at any timepoint and protein level decrease (log2 < -0.3-fold change over untreated control, adjusted *P* value < 0.05) at any timepoint across four or five biological repeats per condition. Heatmap colours indicate fold changes in protein ubiquitination (left) and protein expression (right). Hierarchical clustering was performed on proteins (rows) with Euclidean distance as the similarity metric. The Venn diagram shows the number of ubiquitinated proteins, and the number of proteins depleted at any timepoint including overlapping proteins which are labelled in the heatmap. (**E**) DLD-1 time-resolved proteome and ubiquitome profiling upon USP7 inhibitor treatment with FT671 (10 μM) for 30 min and 360 min. The data was filtered for increased ubiquitination (log2 > twofold change over untreated control, adjusted *P* value < 0.05) at

any timepoint and protein level decrease (log2 < -0.3-fold change over untreated control, adjusted *P* value < 0.05) at any timepoint across four or five biological repeats per condition. Heatmap colours indicate fold changes in protein ubiquitination (left) and protein expression (right). Hierarchical clustering was performed on proteins (rows) with Euclidean distance as the similarity metric. The Venn diagram shows the number of ubiquitinated proteins, and the number of proteins depleted at any timepoint including overlapping proteins labelled in the heatmap. (**F**) Pathway and process enrichment analysis of ubiquitinomic and proteomic analysis using metascape.org (Zhou *et al*, 2019). The top 20 enriched gene ontology terms were quantitatively ranked in a bar plot by their log10 *P* value. (**G**) Protein-protein interaction enrichment analysis of 69 high confidence USP9X substrates in MDA-MB-231 cells (from **Fig 4E**) using metascape.org (Zhou *et al*, 2019). A subset of 34 out of the 69 high confidence substrates was mapped in a protein-protein interaction network. The size of the circles corresponds to the number of mapped interactions.

Appendix Figure S6

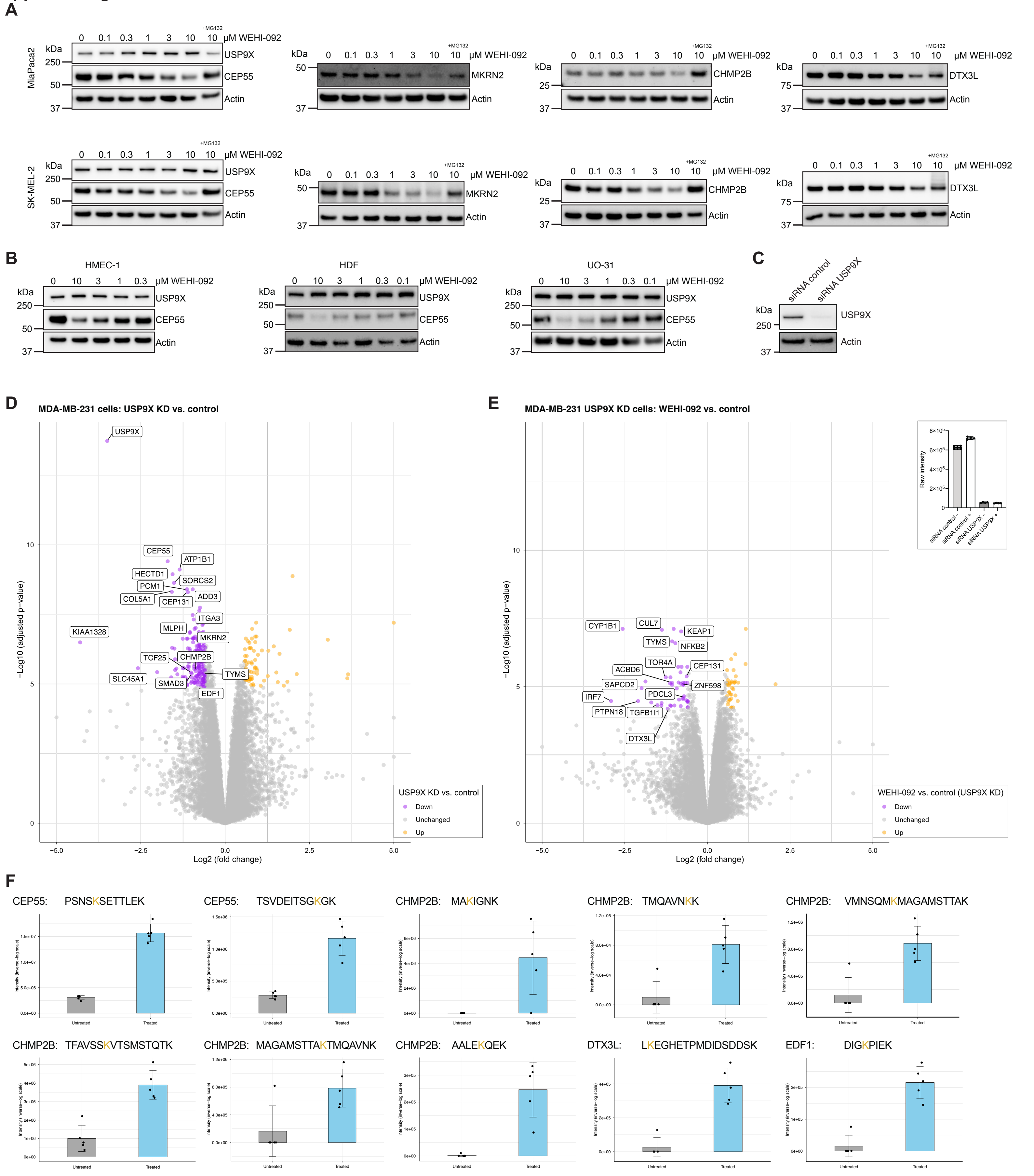

Appendix Fig S6. CEP55 protein levels are reduced in every cell line treated with WEHI-092.

(**A**) Western Blot validation of USP9X and commonly depleted proteins from **Fig 5C** in MiaPaca2 and SK-MEL-2 cells upon WEHI-092 treatment (24 h). Proteasomal inhibition using MG132 was performed at 10 μM for 24 h in presence of 5 μM QVD apoptosis blockage. The blots shown are a representative of at least two biological repeats with Actin as a sample loading control. (**B**) Western Blot validation of USP9X and CEP55 in HMEC-1, human dermal fibroblasts (HDF) and UO-31 cells (24 h). The blots shown are a representative of at least two biological repeats with Actin as a sample loading control. **(C)** Western Blot validation of siRNA-mediated gene silencing of USP9X (USP9X KD) in MDA-MB-231 cells with Actin as a sample loading control from one of five biological repeats. **(D)** Comparison of protein abundance between USP9X KD and control (non-targeting siRNA) MDA-MB-231 cells (from **C**; 96 h post siRNA transfection). Plotted is the average log2 fold change over control cells with four or five biological repeats per condition. Proteins with a depletion of log2 < -0.585 or log2 > 0.585 (adjusted *P* value < 0.05) over control are colour-coded. **(E)** Comparison of protein abundance between WEHI-092-treated (10 μM, 24 h) and control (DMSO treated) USP9X KD MDA-MB-231 cells (from **C**). Plotted is the average log2 fold change over untreated cells, with four or five biological repeats per condition. Proteins with a depletion of log2 < -0.585 or log2 > 0.585 (adjusted *P* value < 0.05) over the untreated condition are colour-coded. The bar plot shows the raw intensities (mean of four or five biological repeats per condition; error bars, s.d.) of USP9X in the proteomic analysis for the cells used in **Figs 5C-E** with "-" indicating untreated conditions and "+" indicating WEHI-092 treated conditions. **(F)**

Detailed visualisation of the increase in abundance of ubiquitinated peptides in commonly depleted proteins (**Fig 5C**) following WEHI-092 treatment (10 μM, 30 min) in MDA-MB-231 cells (data from **Fig 4E**). The data was filtered for log2 > twofold change over untreated control, adjusted *P* value < 0.05 and absolute abundance is shown. In the treated (WEHI-092, 10 μM, 30 min) condition, peptide levels were corrected for global protein abundance. Lysine (K) ubiquitination is highlighted in the peptide sequence by colour. Data shown is the mean of four or five biological repeats where each datapoint is one repeat; error bars, s.d.

Appendix Figure S7

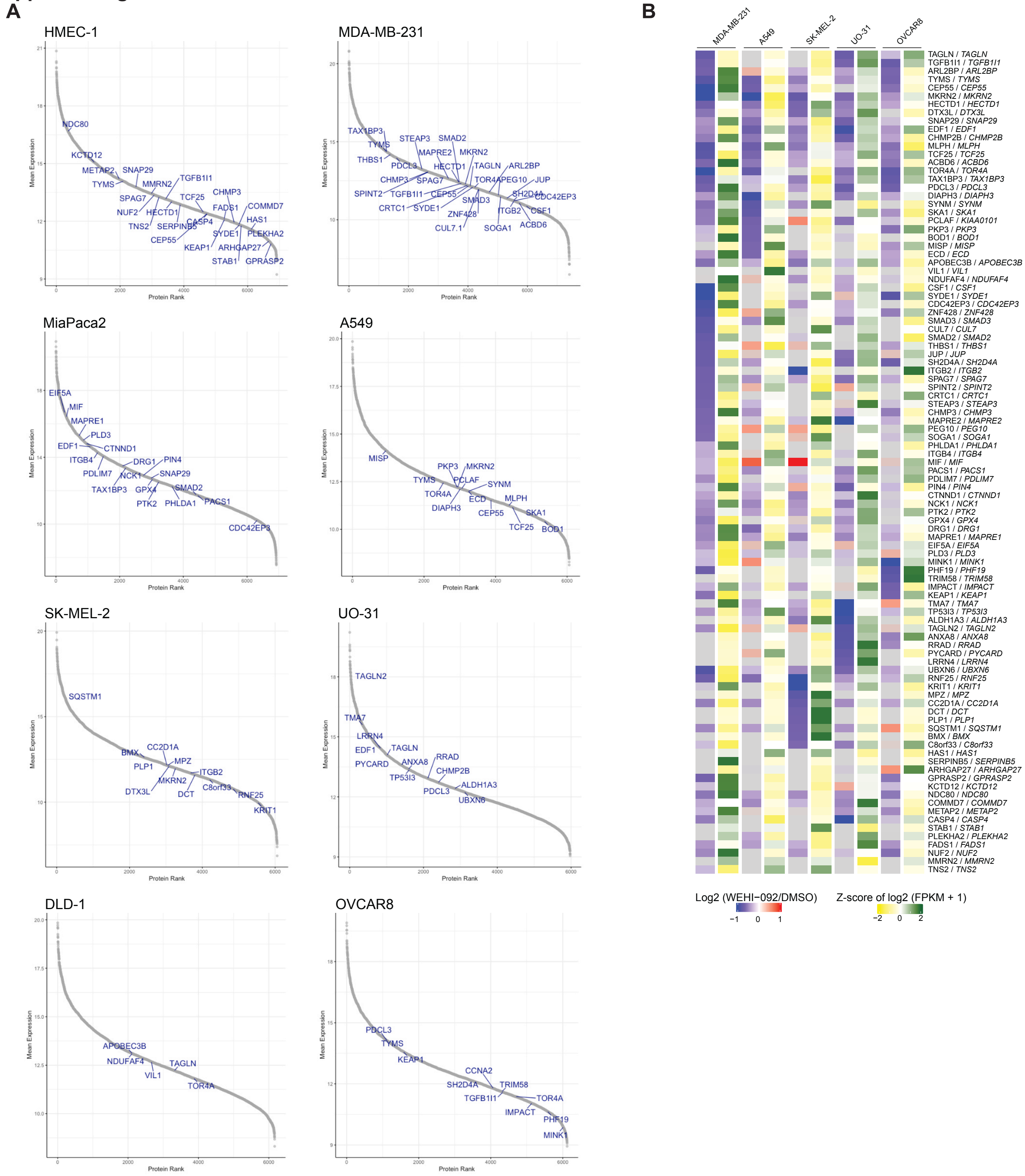

Appendix Fig S7. Proteins depleted upon WEHI-092 treatment vary in expression levels in the eight different cell lines used.

(**A**) Proteins ranked by expression as a mean over four or five biological repeats (untreated control samples used). Proteins found to be depleted upon WEHI-092 treatment (**Fig 6A**) are labelled (filtered for log2 < -0.585-fold change over untreated control condition, adjusted *P* value < 0.05) in each cell line shown. **(B)** Analysis of expression levels of 99 proteins (from **Fig 6A**, comprised of proteins that are depleted by log2 < -0.585 [adjusted *P* value < 0.05] over untreated control in at least one out of the eight cell lines tested with four or five biological repeats per cell line and condition). Data shown side by side with the data from **Fig 6A** is gene expression (Z-score of log2 fragments per kilobase per million reads [FPKM] + 1) from CellMiner NCI-60 database for the cell lines with data available.

Appendix Figure S8

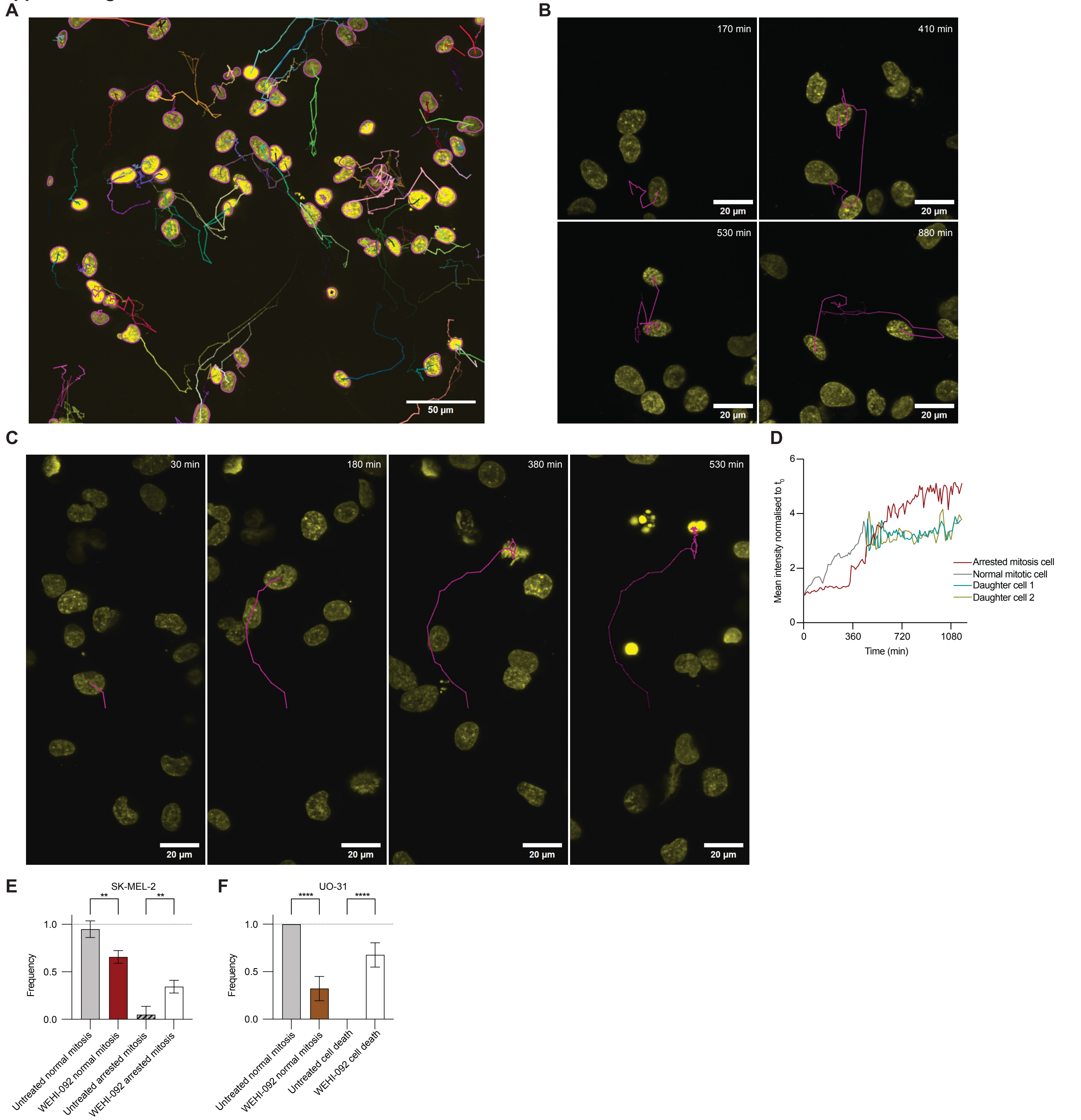

Appendix Fig S8. Lattice light sheet imaging analysis.

(**A**) Demonstration of a full field of MDA-MB-231 cells with associated segmentation, via StarDist, and associated individual cell tracks, using an advanced Kalman tracker capable of merging and splitting tracks. Scale bar: 50 µm. (**B**) Example of a mitotic MDA-MB-231 cell (yellow) with its associated track (pink). Following mitosis, the track continues to follow the two daughter cells. Scale bar: 20 µm. (**C**) Example of mitotic arrest of MDA-MB-231 cell (yellow) with associated track (pink) over time left to right. Scale bar: 20 µm. (**D**) Plot of mean pixel intensity over time for normal mitotic cell from **B**, shown in a grey line and arrested mitosis cell from **C**, shown in a red line. Grey line depicts mean intensity for a single cell as it enters mitosis with the two green lines denoting the mean pixel intensity for each daughter cell. The data was normalized to t_0. **(E)** Quantification of mitosis phenotype in SK-MEL-2 cells similar to **Fig 6D**. In the bar graph, the mean across three independent biological repeats is shown with a total of 42 divisions (untreated) and 36 divisions (WEHI-092); error bars, s.d. One-way ANOVA with post-hoc multiple comparison Tukey test was performed for significance testing. Significance level ** p = 0.0071. **(F)** Quantification of cell death phenotype in UO-31 cells. In the bar graph, the mean across four independent biological repeats is shown with a total of 53 cells (untreated) and 45 cells (WEHI-092) analysed; error bars, s.d. One-way ANOVA with post-hoc multiple comparison Tukey test was performed for significance testing. Significance level **** $p < 0.0001$.

Appendix Figure S9

A

^{1}H NMR of WEHI-092

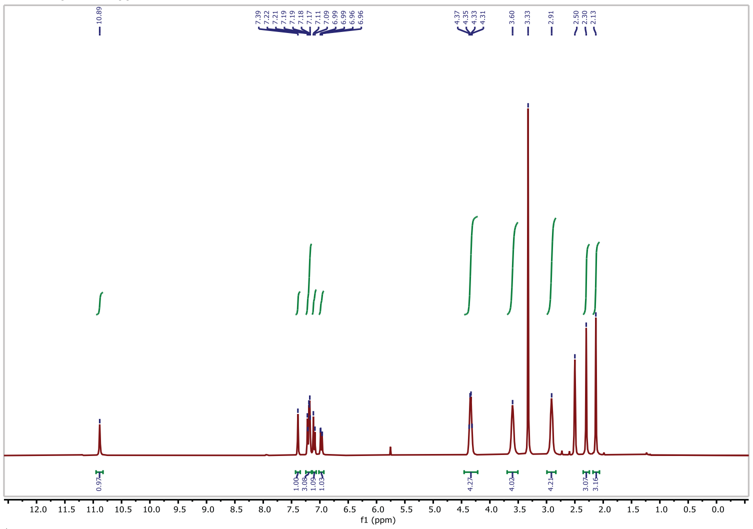

^{13}C NMR of WEHI-092

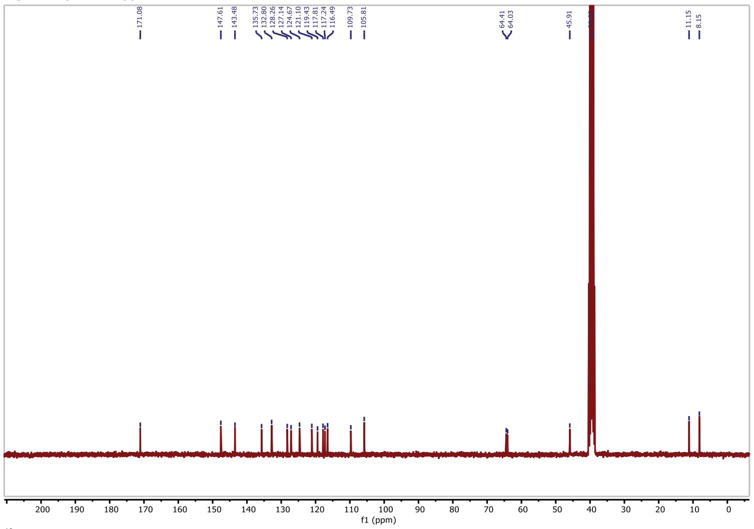

^{1}H NMR of WEHI-333

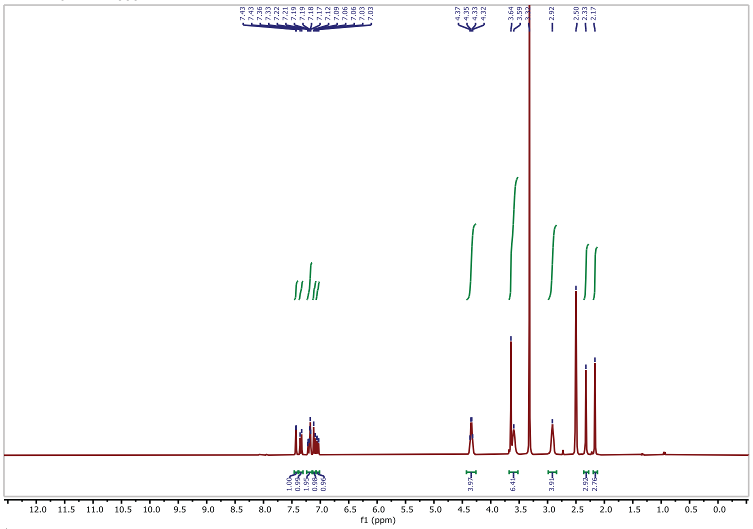

^{13}C NMR of WEHI-333

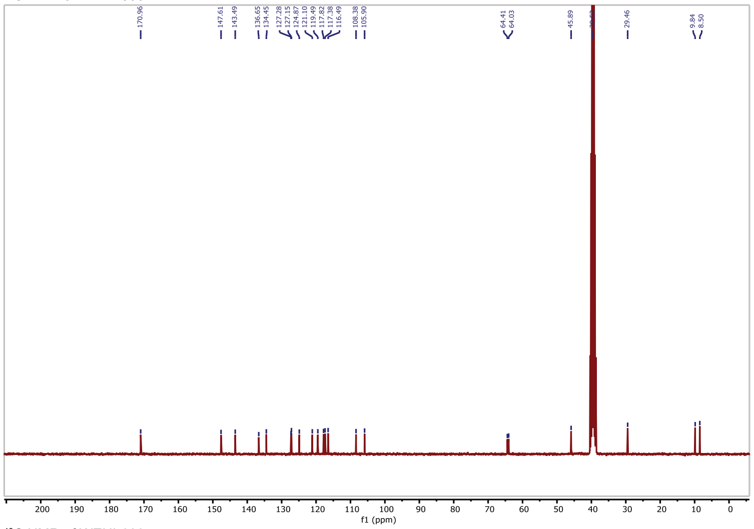

^{1}H NMR of WEHI-680

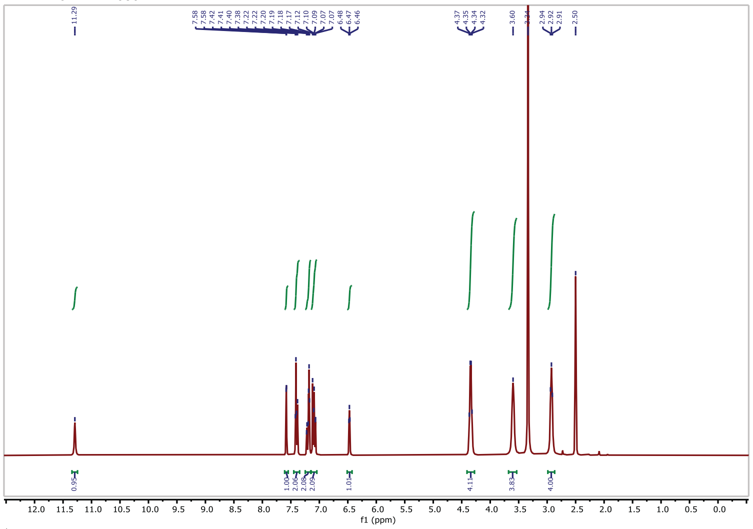

^{13}C NMR of WEHI-680

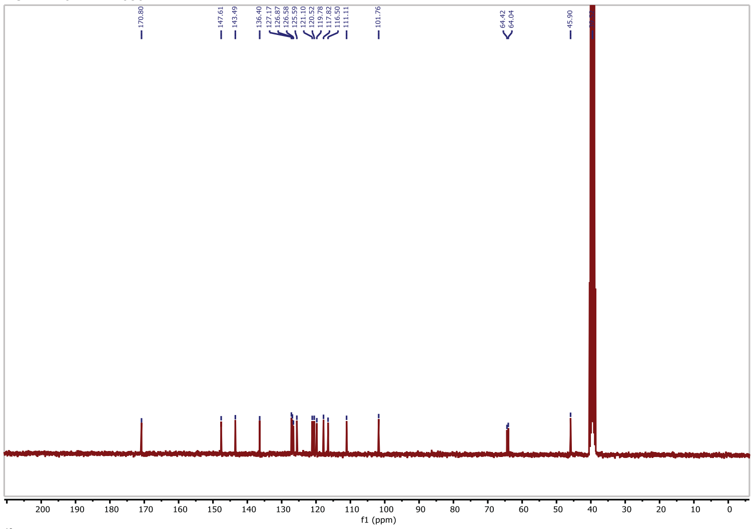

^{1}H NMR of WEHI-553

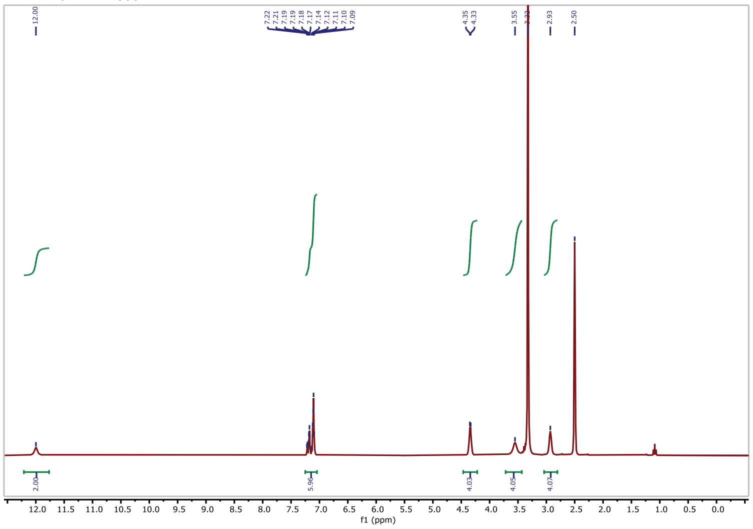

^{13}C NMR of WEHI-553

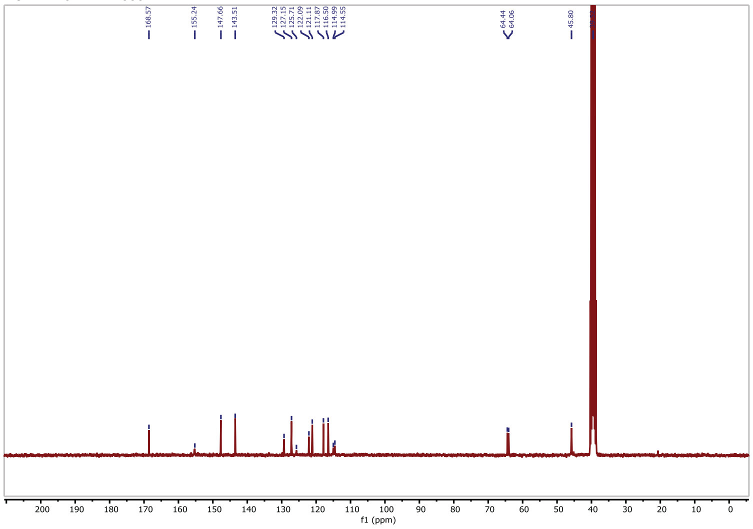

Appendix Fig S9. NMR data for WEHI series compounds.

(**A**) ^{1}H NMR (300 MHz, DMSO; shown on the left) and ^{13}C NMR (75 MHz, DMSO; shown on the right) spectra for WEHI series compounds.

Appendix Table S1. List of cell lines including culture conditions

Cell line	Growth media	Source
MDA-MB-231	DMEM + 10% FBS	WEHI
MDA-MB-468	RMPI + 10% FBS + 1X GlutaMAX + 5 µg/mL Insulin	WEHI
MCF-7	DMEM + 10% FBS + 1X Glutamine	WEHI
A549	DMEM + 10% FBS	WEHI
ACHN	RPMI + 10% FBS	WEHI
CAKI-1	RPMI + 10% FBS	WEHI
A498	RPMI + 10% FBS	WEHI
UO-31	RPMI + 10% FBS	WEHI
MiaPaca2	DMEM + 10% FBS + 2.5% horse serum	WEHI
PANC-1	DMEM + 10% FBS	WEHI
BxPC3	RPMI + 10% FBS	WEHI
DLD-1	DMEM + 10% FBS + 1X GlutaMAX	WEHI
HMEC-1	Endothelial Cell Growth Medium (Sigma-Aldrich, 211-500)	WEHI
HUVEC	Endothelial Cell Growth Medium (Sigma-Aldrich, 211-500)	StemCell Technologies (US), Female
Human dermal fibroblasts (HDF)	DMEM + 20% FBS	Lonza (Switzerland); 19TL178960, P1 58Y, Female
A375	DMEM + 10% FBS	WEHI
SK-MEL-2	RPMI + 10% FBS	WEHI
SK-MEL-28	RPMI + 10% FBS	WEHI
Huh7	DMEM + 10% FBS	WEHI
HepG2	DMEM + 10% FBS	WEHI
OVCAR8	DMEM + 10% FBS + 5 µg/mL Insulin + 0.05 µg/mL EGF + 1 µg/mL Hydrocortisone	WEHI
THP-1	RPMI + 10% FBS	WEHI

Appendix Material and Methods

Medicinal chemistry: All reagents were used as received from commercial suppliers unless stated otherwise. NMR spectra were recorded at ambient temperature on a Bruker Avance 300 MHz instrument in the specified deuterated solvent. Observed proton chemical shifts were reported as units of parts per million (ppm) relative to the respective residual solvent peak DMSO-d6 (δ 2.50). Multiplicities were reported: s (singlet), d (doublet), t (triplet), q (quartet), dd (doublet of doublets), and m (multiplet). High-resolution mass spectrometry analyses were carried out at the analytical lab at the LaTrobe University at the Bundoora campus on an Agilent 6530 TOF LC/MS Mass Spectrometer coupled to an Agilent 1290 Infinity (Agilent, United States). All data were acquired, and reference mass corrected via a dual-spray electrospray ionisation (ESI) source. Acquisition was performed using the Agilent OpenLab ECM XT software. Coupling constants were reported as a *J* value in Hertz (Hz). Abbreviations: DCM (dichloromethane), DMF (*N*,*N*-dimethylformamide), DIPEA (*N*,*N*-diisopropylethylamine), EDCI (*N*-(3-dimethylaminopropyl)-*N'*-ethylcarbodiimide), 1-hydroxybenzotriazole (HOBt) and methanol (MeOH).

Commercial compounds including EOAI3402143, FT671, FT709, PR619 and WP1130 were sourced from MedChemExpress. The negative control compound WEHI-871 3-(4-((2,3-dihydrobenzo[*b*][1,4]dioxin-6-yl)sulfonyl)piperazin-1-yl)-6-phenylpyridazine was sourced from Enamine.

Synthetic procedures for (4-((2,3-dihydrobenzo[*b*][1,4]dioxin-6-yl)sulfonyl)piperazin-1-yl)(2,3-dimethyl-1*H*-indol-5-yl)methanone **(WEHI-092)**, (4-((2,3-

dihydrobenzo[*b*][1,4]dioxin-6-yl)sulfonyl)piperazin-1-yl)(1,2,3-trimethyl-1*H*-indol-5-yl)methanone **(WEHI-333)**, (4-((2,3-dihydrobenzo[*b*][1,4]dioxin-6-yl)sulfonyl)piperazin-1-yl)(1*H*-indol-5-yl)methanone **(WEHI-680)** and 6-(4-((2,3-dihydrobenzo[*b*][1,4]dioxin-6-yl)sulfonyl)piperazin-1-carbonyl)quinoxaline-2,3(1*H*,4*H*)-dione **(WEHI-553)** are described in the following section (see also **Appendix Fig S1A**).

(4-((2,3-Dihydrobenzo[*b*][1,4]dioxin-6-yl)sulfonyl)piperazin-1-yl)(2,3-dimethyl-1*H*-indol-5-yl)methanone **(WEHI-092)**

To a stirred solution of 2,3-dimethyl-1*H*-indole-5-carboxylic acid (206 mg, 1.09 mmol), HOBt hydrate (184 mg, 1.20 mmol) and EDCI hydrochloride (251 mg, 1.31 mmol) in DMF (5 mL) was added DIPEA (665 µL, 3.82 mmol) and 1-((2,3-dihydrobenzo[*b*][1,4]dioxin-6-yl)sulfonyl)piperazine hydrochloride (350 mg, 1.09 mmol). The solution was stirred at ambient temperature for 16 h. The solution was poured onto ice/water (200 mL), whereby a precipitate formed. The solid was filtered and washed with water to give crude product (487 mg). This was purified by column chromatography (10% MeOH in DCM), fractions combined, and solvent evaporated under reduced pressure. Water was added and the whole stirred, filtered and washed with water to give (4-((2,3-dihydrobenzo[*b*][1,4]dioxin-6-yl)sulfonyl)piperazin-1-yl)(2,3-dimethyl-1*H*-indol-5-yl)methanone as a beige solid (431 mg, 87%). ^{1}H NMR (300 MHz, DMSO; **Appendix Figure S9A**) δ 10.89 (s, 1H), 7.39 (s, 1H), 7.23 – 7.15 (m, 3H), 7.10 (d, *J* = 8.3 Hz, 1H), 6.97 (dd, *J* = 8.3, 1.6 Hz, 1H), 4.34 (q, *J* = 5.2 Hz, 4H), 3.65 – 3.55 (m, 4H), 2.96 – 2.86 (m, 4H), 2.30 (s, 3H), 2.13 (s, 3H). ^{13}C NMR (75 MHz, DMSO; **Appendix Figure S9A**) δ 171.1, 147.6, 143.5, 135.7, 132.8,

128.3, 127.1, 124.7, 121.1, 119.4, 117.8, 117.2, 116.5, 109.7, 105.8, 64.4, 64.0, 45.9, 11.2, 8.2. High-resolution electrospray mass spectra (HR-ESMS) calculated for $C_{23}H_{26}N_3O_5S^+$ [M + H] 456.1588, found 456.1597.

(4-((2,3-dihydrobenzo[*b*][1,4]dioxin-6-yl)sulfonyl)piperazin-1-yl)(1,2,3-trimethyl-1*H*-indol-5-yl)methanone **(WEHI-333)**

To a stirred solution of 1,2,3-trimethylindole-5-carboxylic acid (33 mg, 0.162 mmol), HOBt hydrate (26 mg, 0.170 mmol) and EDCI hydrochloride (36 mg, 0.188 mmol) in DMF (1 mL) was added DIPEA (95 µL, 0.546 mmol) and 1-((2,3-dihydrobenzo[*b*][1,4]dioxin-6-yl)sulfonyl)piperazine hydrochloride (50 mg, 0.156 mmol). The solution was stirred at ambient temperature for 16 h. The solution was poured onto ice/water (50 mL) and stirred at ambient temperature for 2 h, whereby a precipitate formed. The solid was filtered, washed with water to give (4-((2,3-dihydrobenzo[*b*][1,4]dioxin-6-yl)sulfonyl)piperazin-1-yl)(1,2,3-trimethyl-1*H*-indol-5-yl)methanone as a beige solid (58 mg, 79%). ^{1}H NMR (300 MHz, DMSO; **Appendix Figure S9A**) δ 7.43 (d, *J* = 1.5 Hz, 1H), 7.34 (d, *J* = 8.4 Hz, 1H), 7.23 – 7.15 (m, 2H), 7.10 (d, *J* = 8.3 Hz, 1H), 7.05 (dd, *J* = 8.4, 1.6 Hz, 1H), 4.34 (q, *J* = 4.7 Hz, 4H), 3.64 (s, 3H), 3.59 (br s, 4H), 2.92 (br s, 4H), 2.33 (s, 3H), 2.17 (s, 3H). ^{13}C NMR (75 MHz, DMSO; **Appendix Figure S9A**) δ 171.0, 147.6, 143.5, 136.7, 134.5, 127.3, 127.2, 124.9, 121.1, 119.5, 117.8, 117.4, 116.5, 108.4, 105.9, 64.4, 64.0, 45.9, 29.5, 9.8, 8.5. HR-ESMS calculated for $C_{24}H_{28}N_3O_5S^+$ [M + H] 470.1744, found 470.1760.

(4-((2,3-dihydrobenzo[*b*][1,4]dioxin-6-yl)sulfonyl)piperazin-1-yl)(1*H*-indol-5-yl)methanone **(WEHI-680)**

To a stirred solution of 1*H*-indole-5-carboxylic acid (26 mg, 0.161 mmol), HOBt hydrate (26 mg, 0.170 mmol) and EDCI hydrochloride (36 mg, 0.188 mmol) in DMF (1 mL) was added DIPEA (95 µL, 0.546 mmol) and 1-((2,3-dihydrobenzo[*b*][1,4]dioxin-6-yl)sulfonyl)piperazine hydrochloride (50 mg, 0.156 mmol). The solution was stirred at ambient temperature for 16 h. The solution was poured onto ice/water (50 mL) and stirred at ambient temperature for 2 h, whereby a precipitate formed. The solid was filtered, washed with water to give (4-((2,3-dihydrobenzo[*b*][1,4]dioxin-6-yl)sulfonyl)piperazin-1-yl)(1*H*-indol-5-yl)methanone as a beige solid (64.0 mg, 96%). ^{1}H NMR (300 MHz, DMSO; **Appendix Figure S9A**) δ 11.29 (s, 1H), 7.58 (d, *J* = 1.5 Hz, 1H), 7.44 – 7.36 (m, 2H), 7.24 – 7.16 (m, 2H), 7.14 – 7.05 (m, 2H), 6.47 (t, *J* = 2.4 Hz, 1H), 4.34 (q, *J* = 4.9 Hz, 4H), 3.60 (s, 4H), 2.92 (t, *J* = 4.9 Hz, 4H). ^{13}C NMR (75 MHz, DMSO; **Appendix Figure S9A**) δ 170.8, 147.6, 143.5, 136.4, 127.2, 126.9, 126.6, 125.6, 121.1, 120.5, 119.8, 117.8, 116.5, 111.1, 101.8, 64.4, 64.0, 45.9. HR-ESMS calculated for $C_{21}H_{22}N_3O_5S^+$ [M + H] 428.1275, found 428.1289.

6-(4-((2,3-dihydrobenzo[*b*][1,4]dioxin-6-yl)sulfonyl)piperazin-1-carbonyl)quinoxaline-2,3(1*H*,4*H*)-dione (**WEHI-553**)

To a stirred solution of 2,3-dioxo-1,4-dihydroquinoxaline-6-carboxylic acid hydrate (35 mg, 0.156 mmol), HOBt hydrate (26 mg, 0.170 mmol) and EDCI hydrochloride (35 mg, 0.183 mmol) in DMF (1 mL) was added DIPEA (95 µL, 0.546 mmol) and 1-((2,3-dihydrobenzo[*b*][1,4]dioxin-6-yl)sulfonyl)piperazine hydrochloride (50 mg, 0.156 mmol). The solution was stirred at ambient temperature for 16 h. The solution was poured onto ice/water (50 mL) and stirred at ambient temperature for 2 h, whereby a white precipitate

formed. The solid was filtered, washed with water to give 6-(4-((2,3-dihydrobenzo[*b*][1,4]dioxin-6-yl)sulfonyl)piperazin-1-carbonyl)quinoxaline-2,3(1*H*,4*H*)-dione as a fawn solid (44.2 mg, 60%). ^{1}H NMR (300 MHz, DMSO; **Appendix Figure S9A**) δ 12.00 (s, 2H), 7.23 – 7.07 (m, 6H), 4.34 (d, J = 4.3 Hz, 4H), 3.55 (s, 4H), 2.93 (s, 4H). ^{13}C NMR (75 MHz, DMSO; **Appendix Figure S9A**) δ 168.6, 155.2, 147.7, 143.5, 129.3, 127.2, 125.7, 122.1, 121.1, 117.9, 116.5, 115.0, 114.6, 64.4, 64.1, 45.8. HR-ESMS calculated for $C_{21}H_{21}N_4O_7S^+$ [M + H] 473.1125, found 473.1143.

Appendix References

Cadzow L, Brenneman J, Tobin E, Sullivan P, Nayak S, Ali JA, Shenker S, Griffith J, McGuire M, Grasberger P, et al (2024) The USP1 inhibitor KSQ-4279 overcomes PARP inhibitor resistance in homologous recombination–deficient tumors. Cancer Res 84: 3419–3434

Kategaya L, Lello PD, Rougé L, Pastor R, Clark KR, Drummond J, Kleinheinz T, Lin E, Upton J-P, Prakash S et al (2017) USP7 small-molecule inhibitors interfere with ubiquitin binding. Nature 550:534–538

Kazi NH, Klink N, Gallant K, Kipka G-M, Gersch M (2025) Chimeric deubiquitinase engineering reveals structural basis for specific inhibition of the mitophagy regulator USP30. Nat Struct Mol Biol 32:1776–1786

Patzke JV, Sauer F, Nair RK, Endres E, Proschak E, Hernandez-Olmos V, Sotriffer C, Kisker C (2024) Structural basis for the bi-specificity of USP25 and USP28 inhibitors. EMBO Rep 25:2950–2973

Paudel P, Zhang Q, Leung C, Greenberg HC, Guo Y, Chern Y-H, Dong A, Li Y, Vedadi M, Zhuang Z et al (2019) Crystal structure and activity-based labeling reveal the mechanisms for linkage-specific substrate recognition by deubiquitinase USP9X. Proc Natl Acad Sci USA 116:7288–7297

Rennie ML, Gundogdu M, Arkinson C, Liness S, Frame S, Walden H (2024) Structural and biochemical insights into the mechanism of action of the clinical USP1 inhibitor, KSQ-4279. J Med Chem 67: 15557–15568

Ruiz EJ, Pinto-Fernandez A, Turnbull AP, Lan L, Charlton TM, Scott HC, Damianou A, Vere G, Riising EM, Costa CD et al (2021) USP28 deletion and small-molecule inhibition destabilizes c-MYC and elicits regression of squamous cell lung carcinoma. eLife 10:e71596

Steger M, Demichev V, Backman M, Ohmayer U, Ihmor P, Müller S, Ralser M, Daub H (2021) Time-resolved in vivo ubiquitinome profiling by DIA-MS reveals USP7 targets on a proteome-wide scale. Nat Commun 12:5399

Turnbull AP, Ioannidis S, Krajewski WW, Pinto-Fernandez A, Heride C, Martin ACL, Tonkin LM, Townsend EC, Buker SM, Lancia DR et al (2017) Molecular basis of USP7 inhibition by selective small-molecule inhibitors. Nature 550:481–486

Wang Y, Jiang Y, Ding S, Li J, Song N, Ren Y, Hong D, Wu C, Li B, Wang F et al (2018) Small molecule inhibitors reveal allosteric regulation of USP14 via steric blockade. Cell Res 28:1186–1194

Zhou Y, Zhou B, Pache L, Chang M, Khodabakhshi AH, Tanaseichuk O, Benner C, Chanda SK (2019) Metascape provides a biologist-oriented resource for the analysis of systems-level datasets. Nat Commun 10:1523
