# Supplementary material for: Global analysis of cancer cell responses to USP9X inhibition
Source: EMBO J. 2026 Apr 7;45(9):3306–31. doi: 10.1038/s44318-026-00742-y (PMC13144739; doi:10.1038/s44318-026-00742-y)
Supplement: Supplementary file 3 — Movie EV1 [file 44318_2026_742_MOESM3_ESM.zip › MovieEV1_Legend.docx]

**Movie EV1**

Movie EV1 shows an example of a normal cell division in MDA-MB-231 cells over time. DNA is shown in yellow (right), and tubulin is shown in magenta (middle). Merged channels are shown on the left. Timestamp is in h. Scale bar: 20 µm.
