# Supplementary material for: Global analysis of cancer cell responses to USP9X inhibition
Source: EMBO J. 2026 Apr 7;45(9):3306–31. doi: 10.1038/s44318-026-00742-y (PMC13144739; doi:10.1038/s44318-026-00742-y)
Supplement: Supplementary file 4 — Movie EV2 [file 44318_2026_742_MOESM4_ESM.zip › MovieEV2_Legend.docx]

**Movie EV2**

Movie EV2 shows an example of mitotic arrest in MDA-MB-231 cells over time. DNA is shown in yellow (middle), and tubulin is shown in magenta (right). Merged channels are shown on the left. Timestamp is in h. Scale bar: 20 µm.
