# Supplementary material for: Global analysis of cancer cell responses to USP9X inhibition
Source: EMBO J. 2026 Apr 7;45(9):3306–31. doi: 10.1038/s44318-026-00742-y (PMC13144739; doi:10.1038/s44318-026-00742-y)
Supplement: Supplementary file 6 — Movie EV4 [file 44318_2026_742_MOESM6_ESM.zip › MovieEV4_Legend.docx]

**Movie EV4**

Movie EV4 shows examples of microtubule disassembly upon WEHI-092 (15 µM) treatment in MDA-MB-231 cells. Tubulin is shown in a Fire LUT generated with ImageJ/Fiji.
