# Supplementary material for: Global analysis of cancer cell responses to USP9X inhibition
Source: EMBO J. 2026 Apr 7;45(9):3306–31. doi: 10.1038/s44318-026-00742-y (PMC13144739; doi:10.1038/s44318-026-00742-y)
Supplement: Supplementary file 7 — Movie EV5 [file 44318_2026_742_MOESM7_ESM.zip › MovieEV5_Legend.docx]

**Movie EV5**

Movie EV5 shows an example of the full-field timelapse for UO-31 cells with untreated DMSO control cells on the left and WEHI-092 (15 µM) treated cells on the right. DNA is shown in yellow, tubulin is shown in magenta, and AnnexinV is shown in cyan. Timestamp is in h and 21 h window is shown. Scale bar: 100 µm.
