# Supplementary figures and images for: Global analysis of cancer cell responses to USP9X inhibition
Source: EMBO J. 2026 Apr 7;45(9):3306–31. doi: 10.1038/s44318-026-00742-y (PMC13144739; doi:10.1038/s44318-026-00742-y)

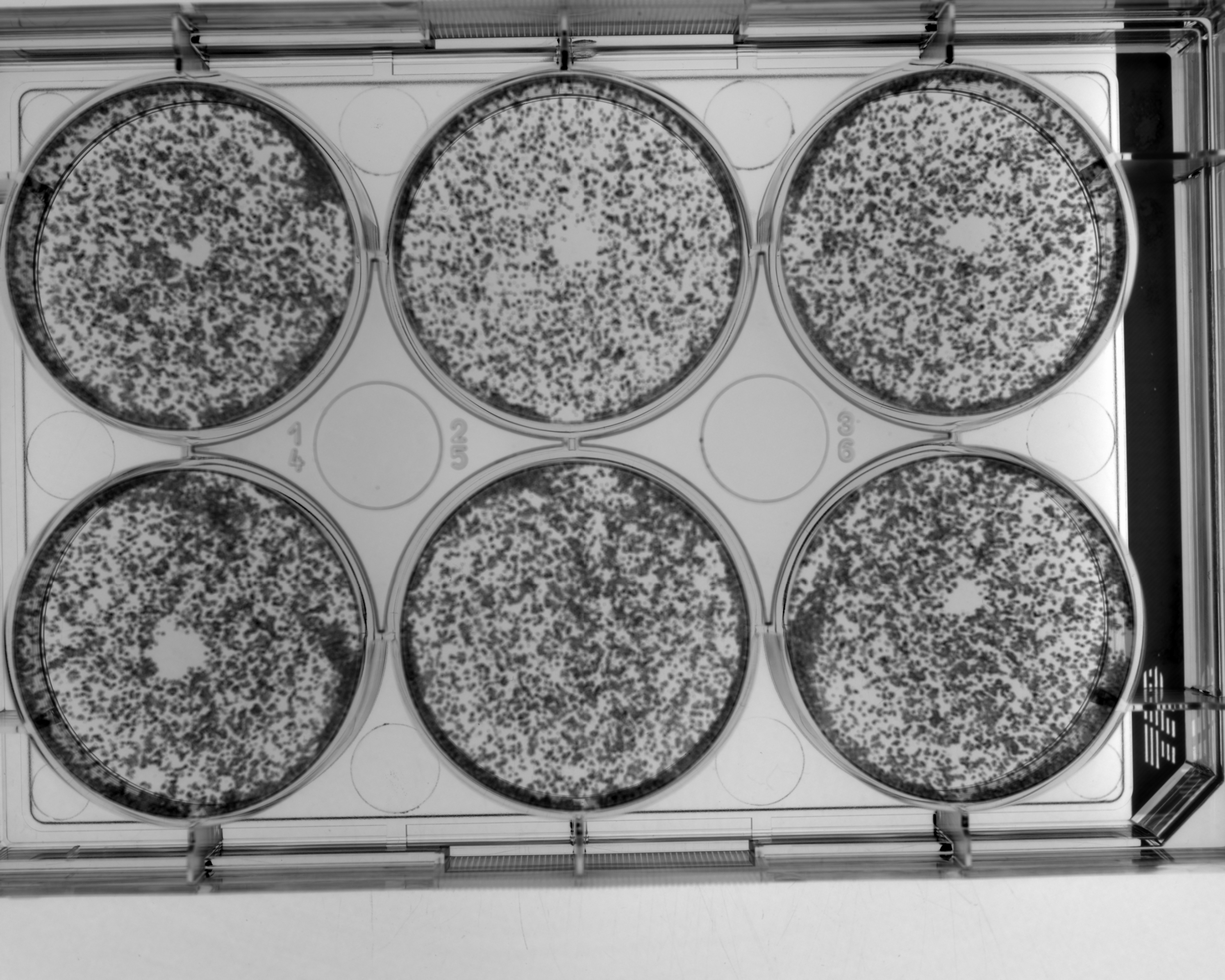

Supplement: Supplementary file 10 — Source data Fig. 3 [file 44318_2026_742_MOESM10_ESM.zip › FIgure 3/3E/BxPC3/BxPC3_3.tif]

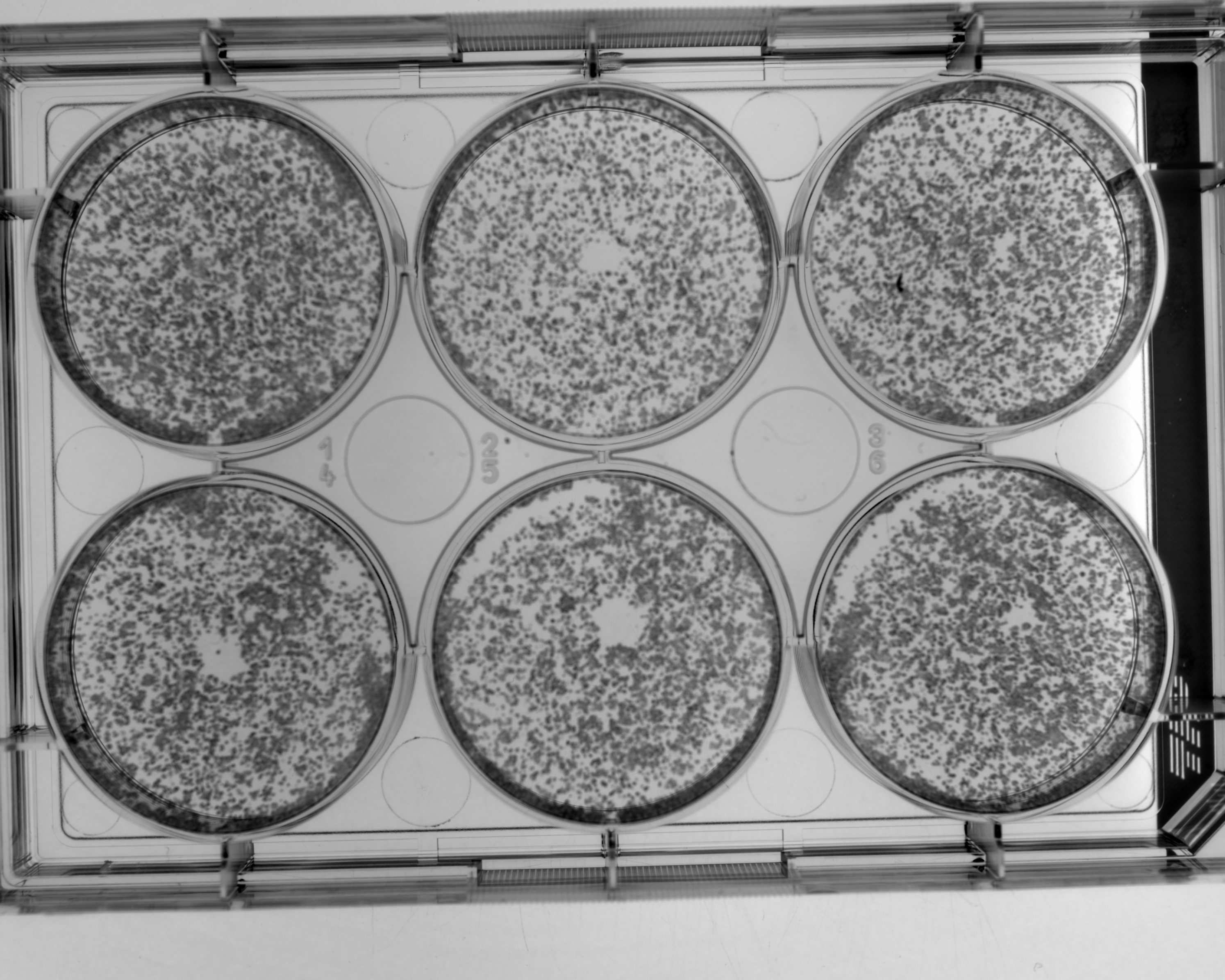

Supplement: Supplementary file 10 — Source data Fig. 3 [file 44318_2026_742_MOESM10_ESM.zip › FIgure 3/3E/BxPC3/BxPC3_2.tif]

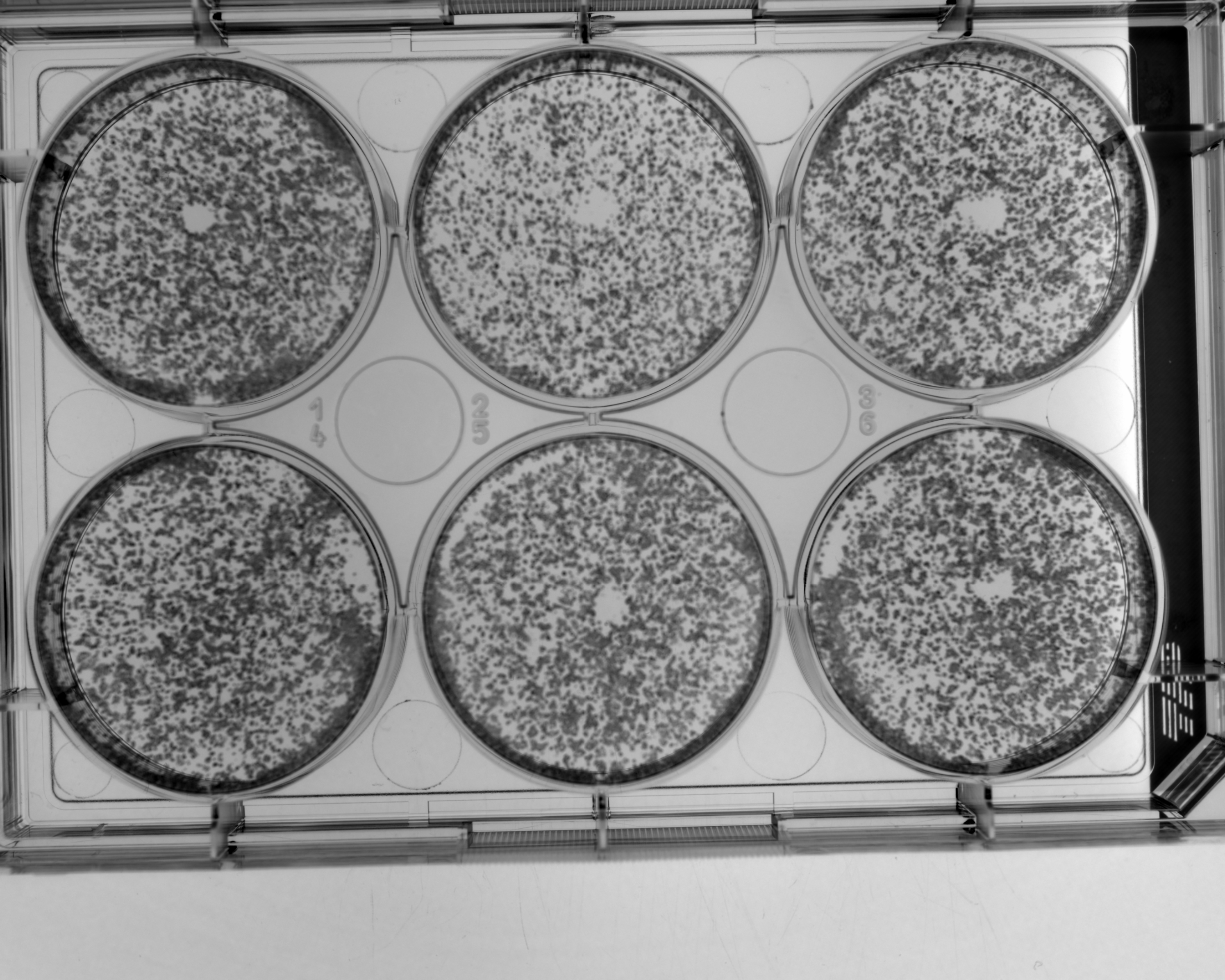

Supplement: Supplementary file 10 — Source data Fig. 3 [file 44318_2026_742_MOESM10_ESM.zip › FIgure 3/3E/BxPC3/BxPC3_1.tif]

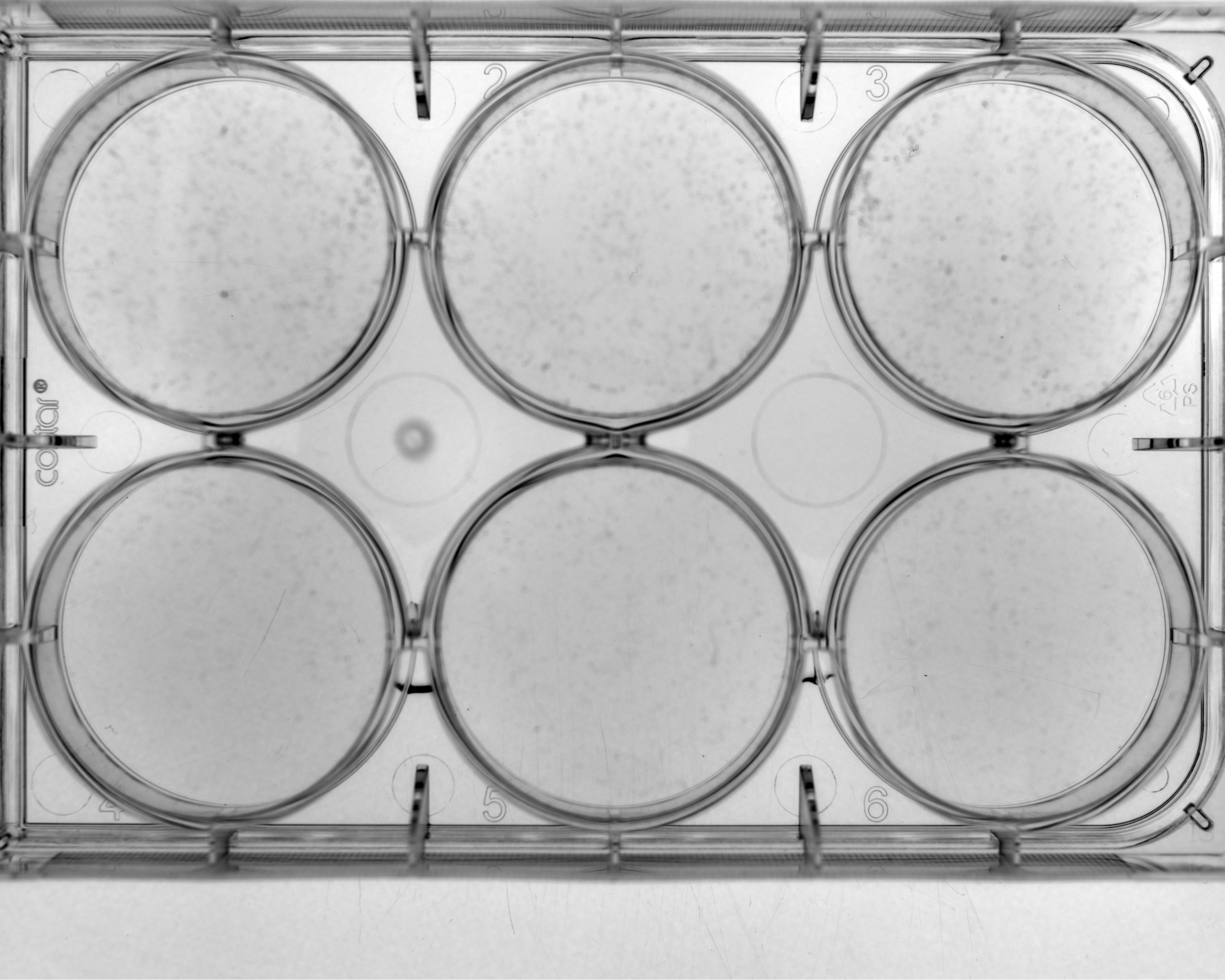

Supplement: Supplementary file 10 — Source data Fig. 3 [file 44318_2026_742_MOESM10_ESM.zip › FIgure 3/3E/MDA-MB-231/MDA-MB-231_2.tif]

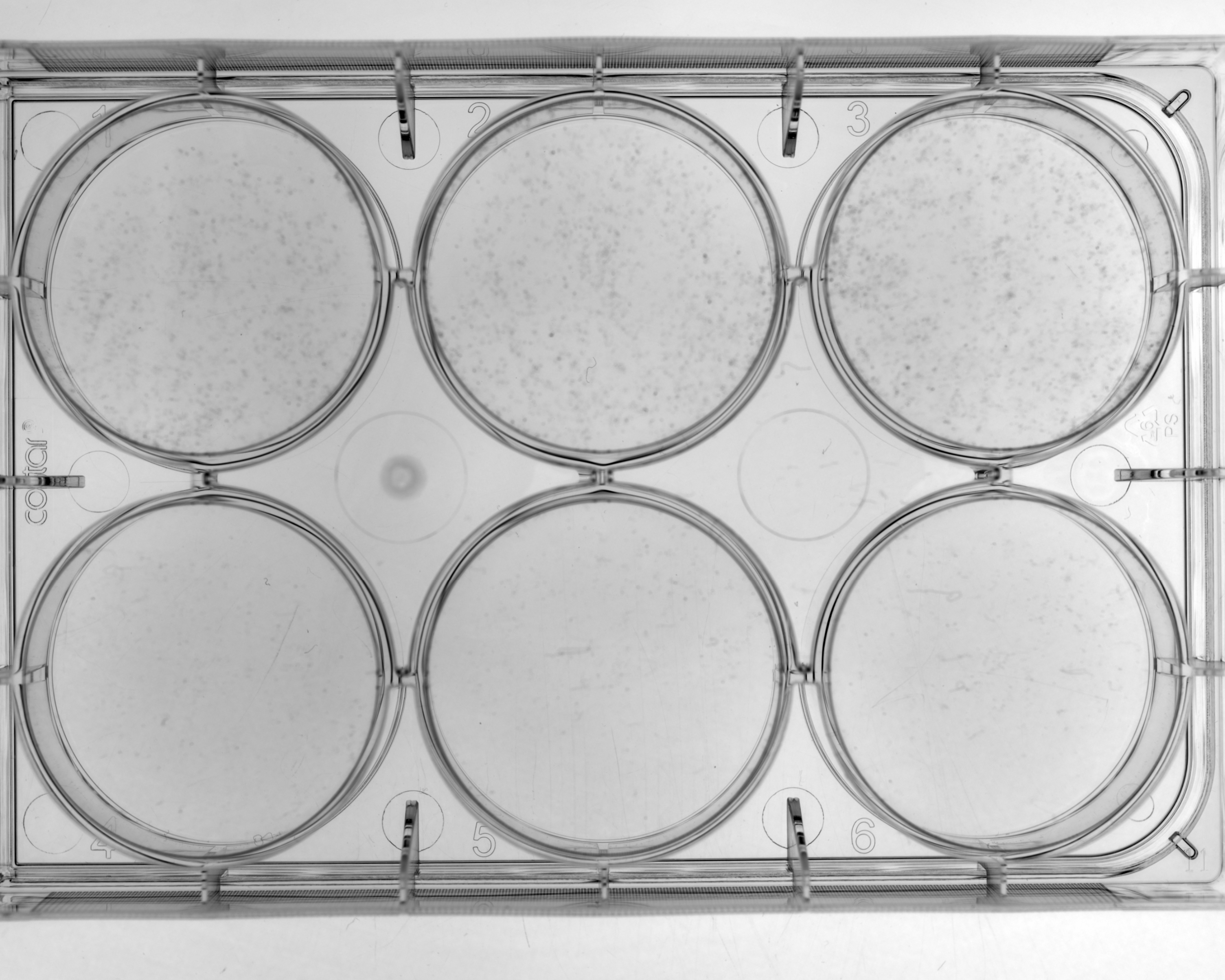

Supplement: Supplementary file 10 — Source data Fig. 3 [file 44318_2026_742_MOESM10_ESM.zip › FIgure 3/3E/MDA-MB-231/MDA-MB-231_3.tif]

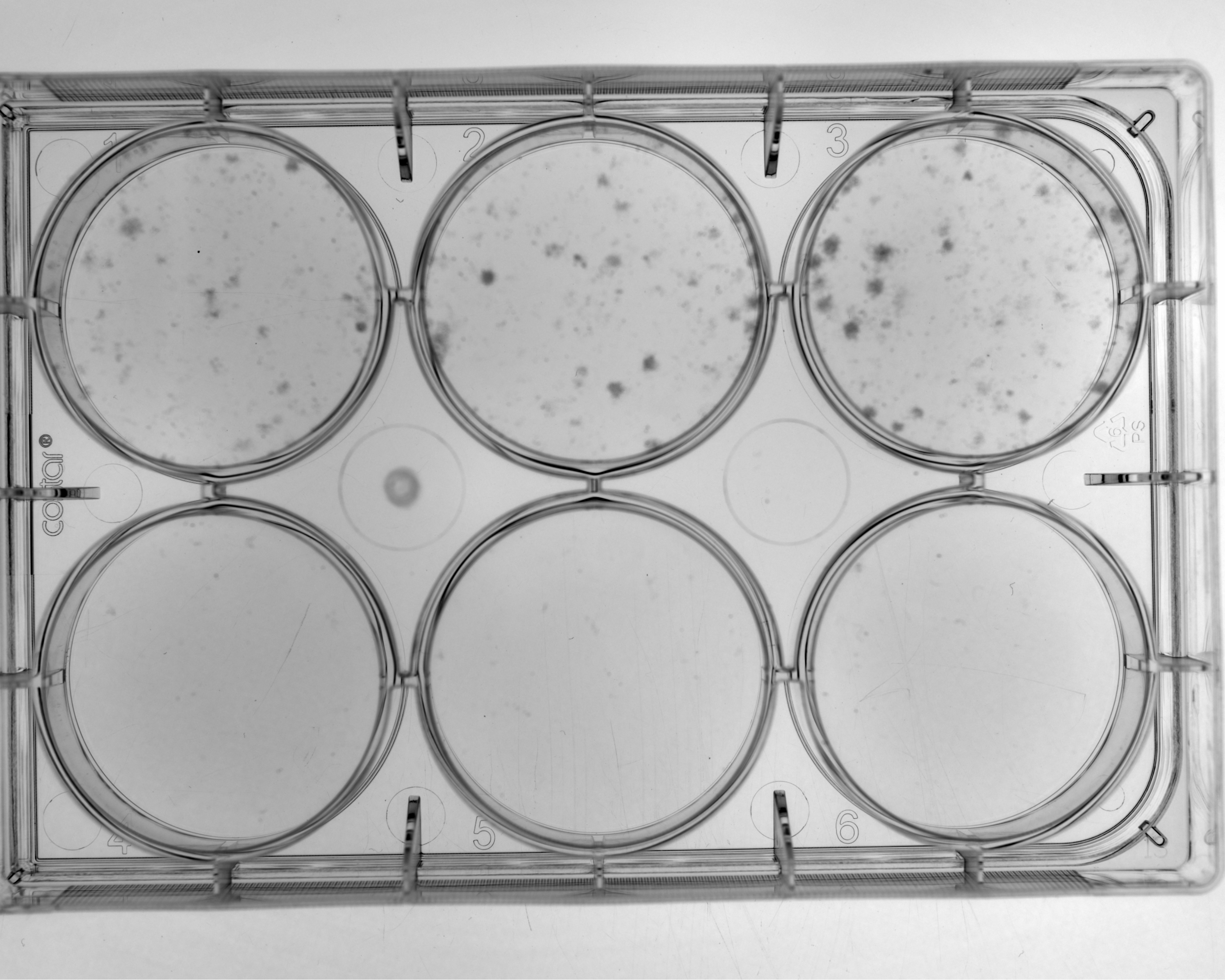

Supplement: Supplementary file 10 — Source data Fig. 3 [file 44318_2026_742_MOESM10_ESM.zip › FIgure 3/3E/MDA-MB-231/MDA-MB-231_1.tif]

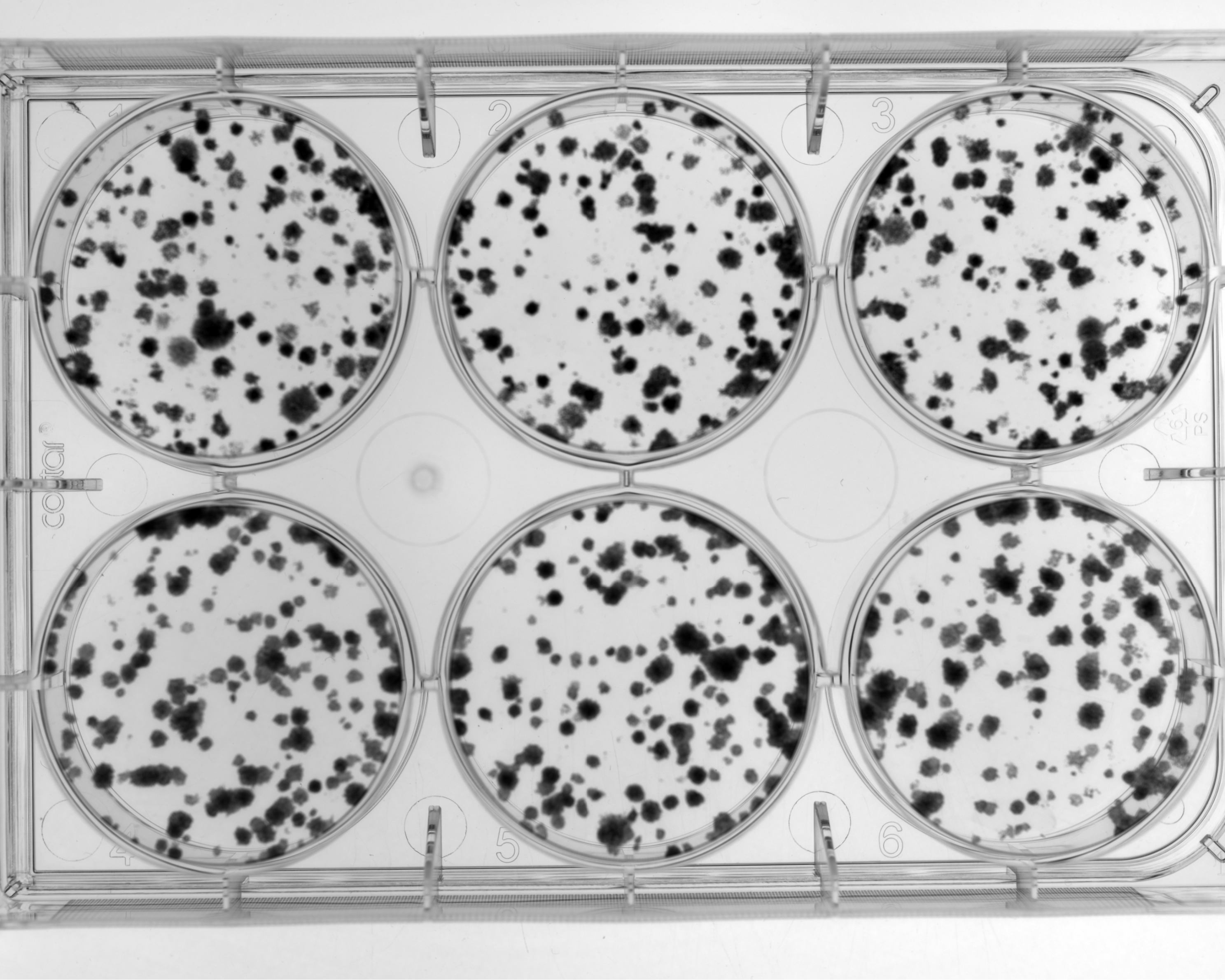

Supplement: Supplementary file 10 — Source data Fig. 3 [file 44318_2026_742_MOESM10_ESM.zip › FIgure 3/3E/ACHN/ACHN_1.tif]

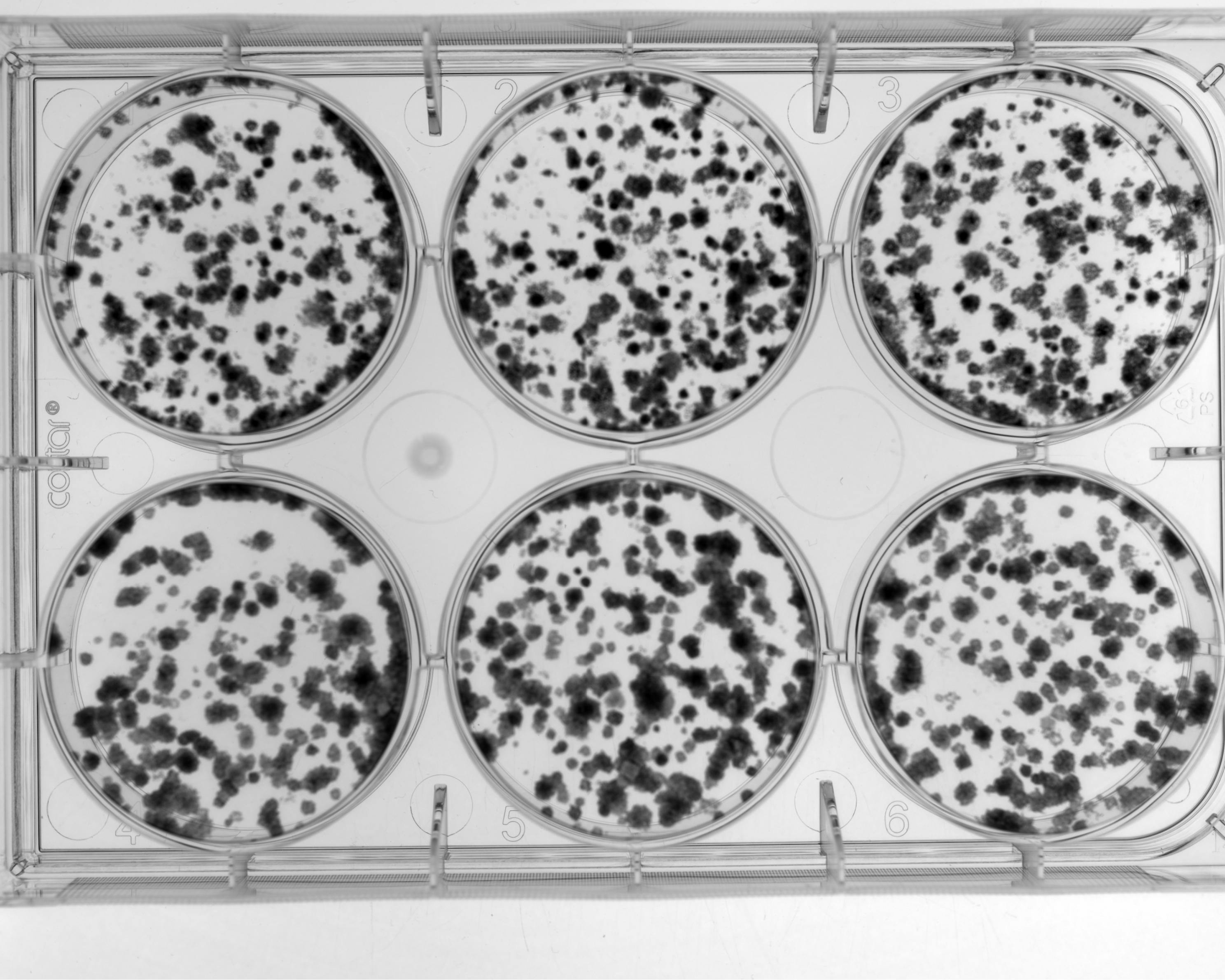

Supplement: Supplementary file 10 — Source data Fig. 3 [file 44318_2026_742_MOESM10_ESM.zip › FIgure 3/3E/ACHN/ACHN_2.tif]

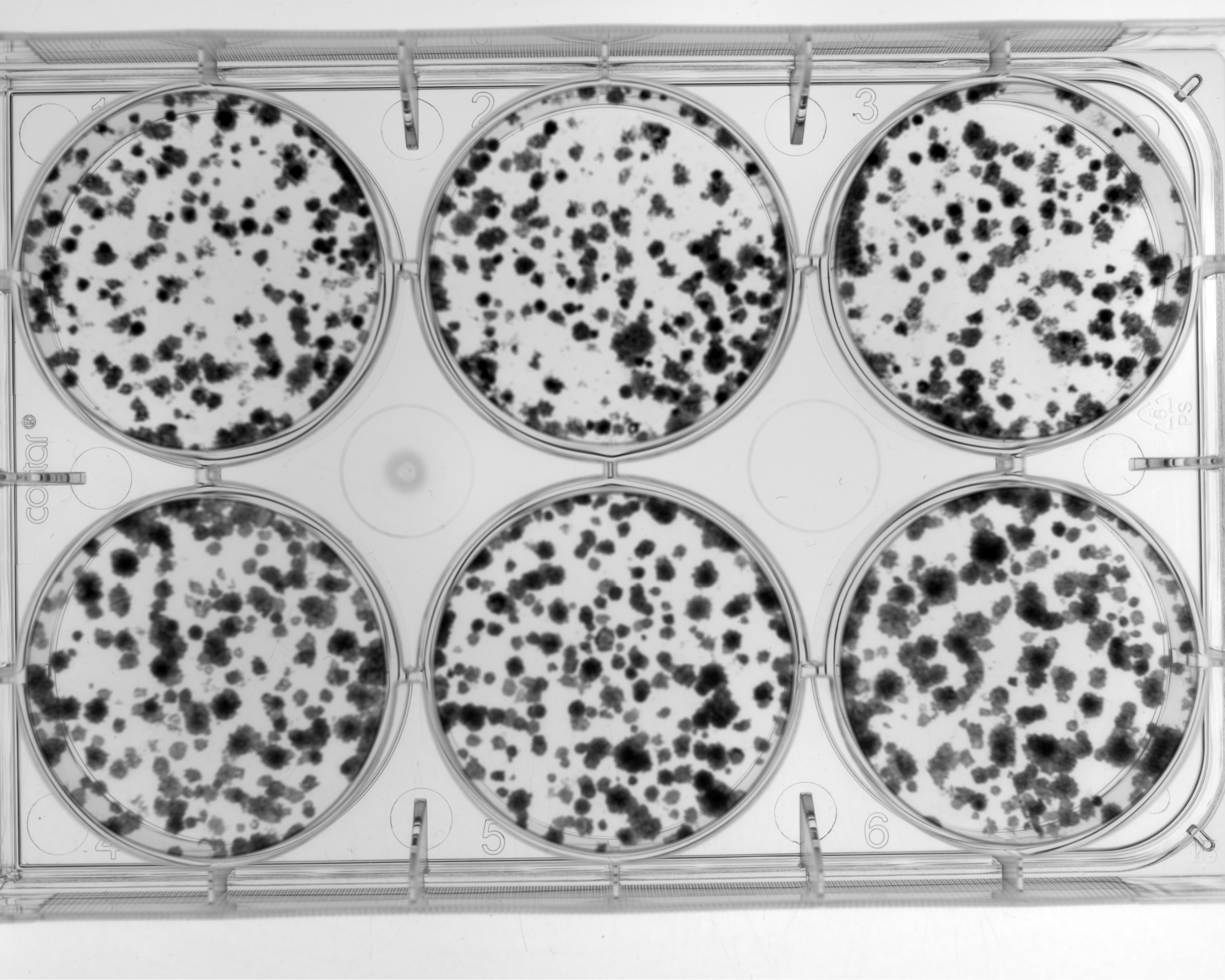

Supplement: Supplementary file 10 — Source data Fig. 3 [file 44318_2026_742_MOESM10_ESM.zip › FIgure 3/3E/ACHN/ACHN_3.tif]

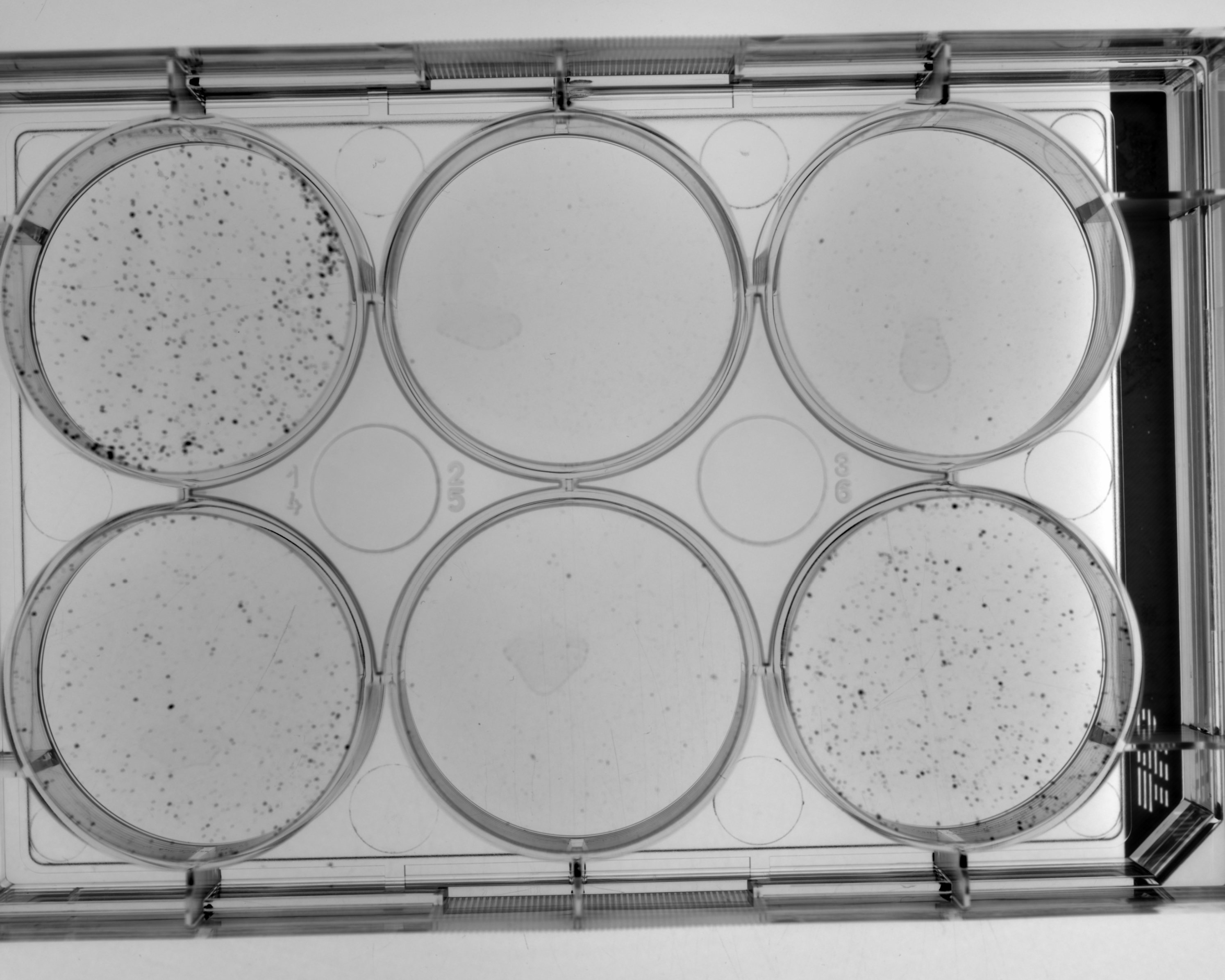

Supplement: Supplementary file 10 — Source data Fig. 3 [file 44318_2026_742_MOESM10_ESM.zip › FIgure 3/3E/MDA-MB-468/MDA-MB-468_3.tif]

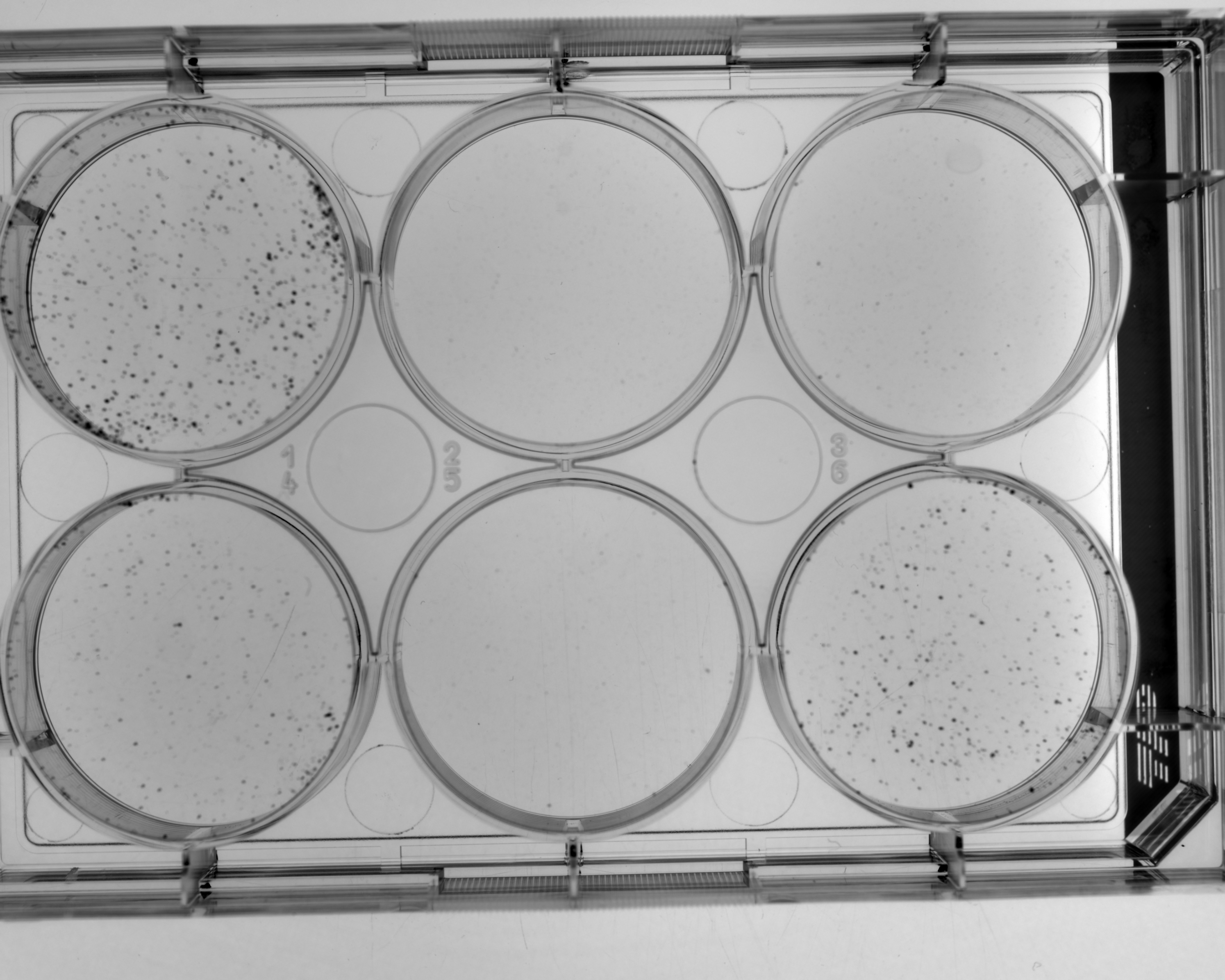

Supplement: Supplementary file 10 — Source data Fig. 3 [file 44318_2026_742_MOESM10_ESM.zip › FIgure 3/3E/MDA-MB-468/MDA-MB-468_2.tif]

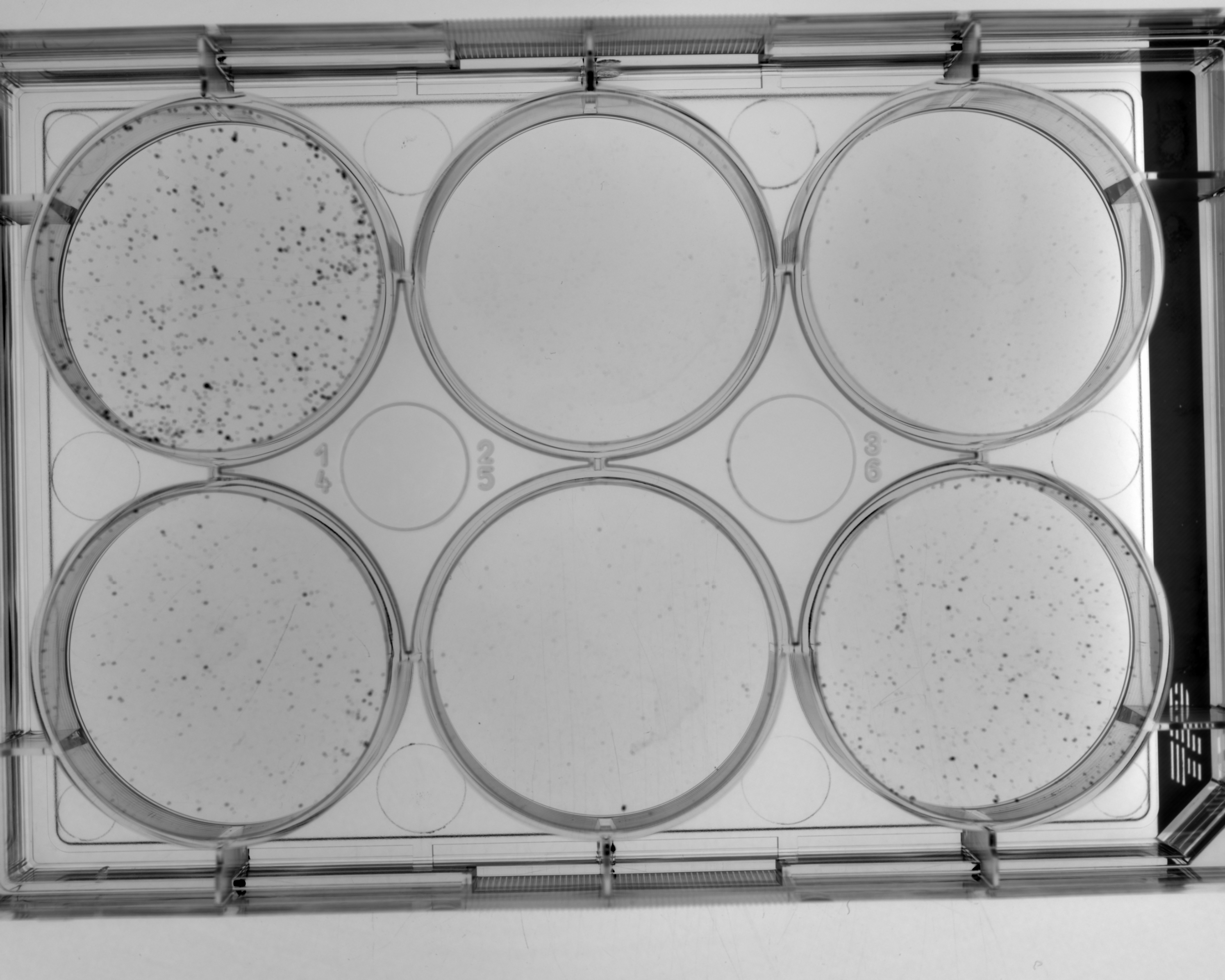

Supplement: Supplementary file 10 — Source data Fig. 3 [file 44318_2026_742_MOESM10_ESM.zip › FIgure 3/3E/MDA-MB-468/MDA-MB-468_1.tif]

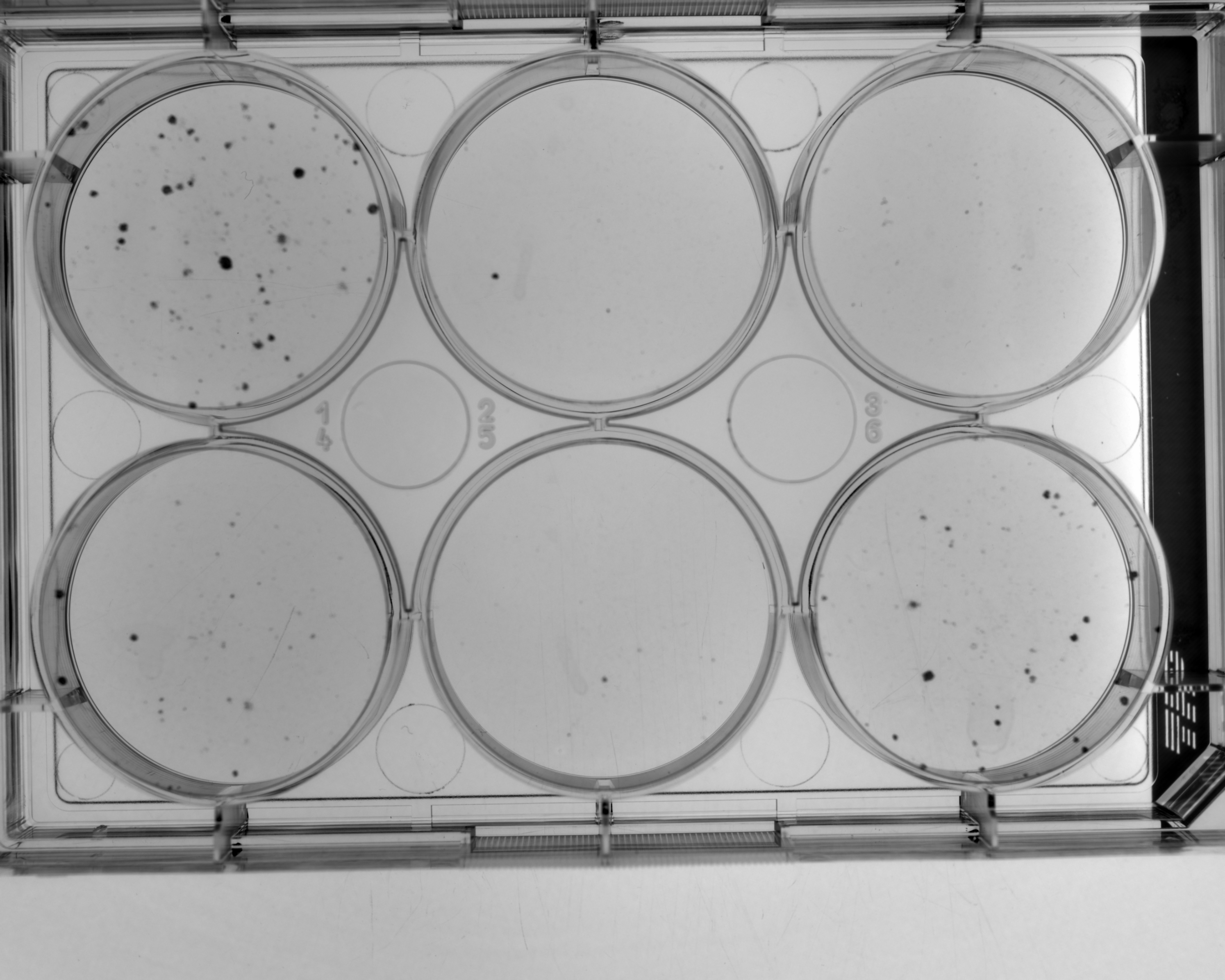

Supplement: Supplementary file 10 — Source data Fig. 3 [file 44318_2026_742_MOESM10_ESM.zip › FIgure 3/3E/PANC-1/PANC-1_3.tif]

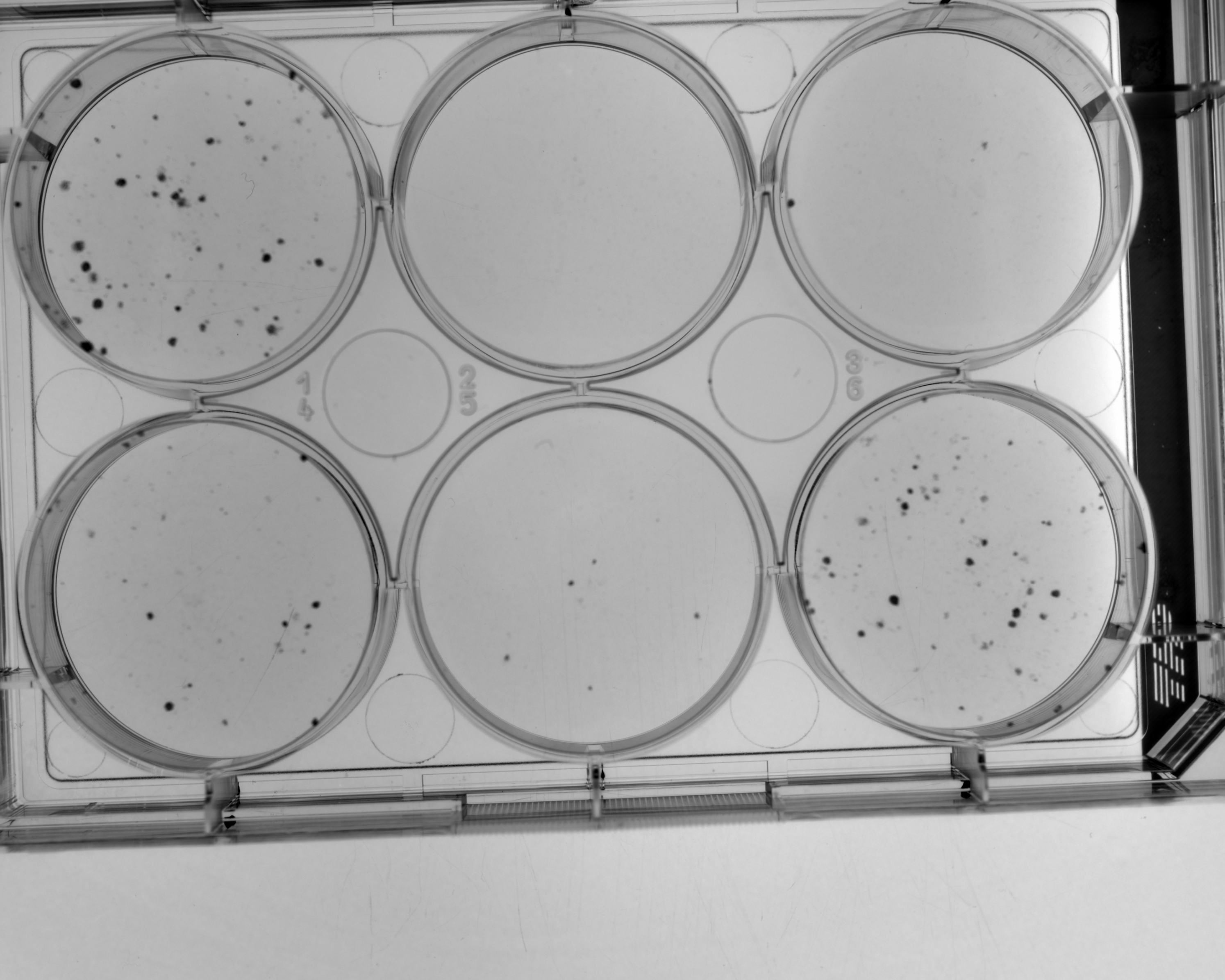

Supplement: Supplementary file 10 — Source data Fig. 3 [file 44318_2026_742_MOESM10_ESM.zip › FIgure 3/3E/PANC-1/PANC-1_2.tif]

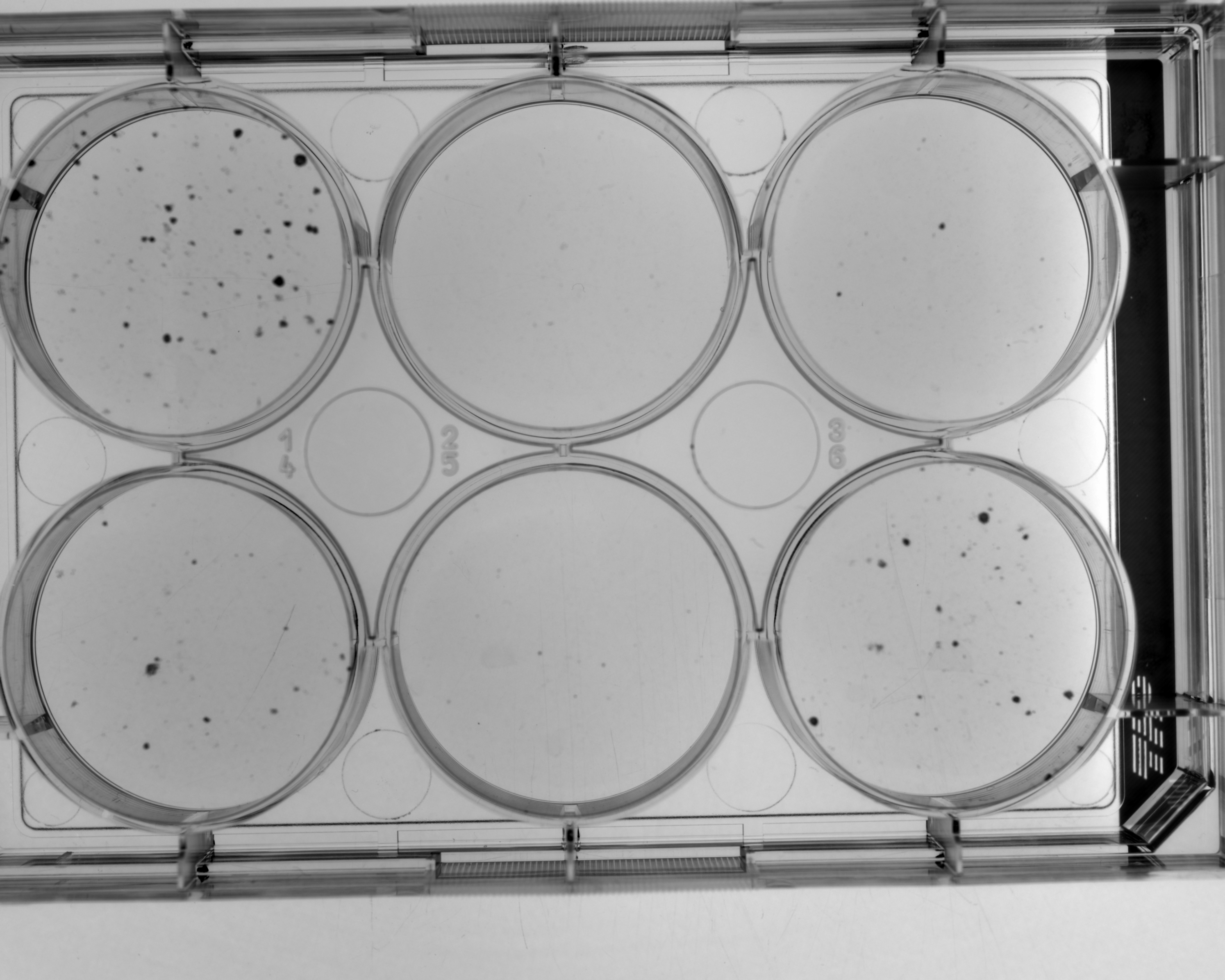

Supplement: Supplementary file 10 — Source data Fig. 3 [file 44318_2026_742_MOESM10_ESM.zip › FIgure 3/3E/PANC-1/PANC-1_1.tif]

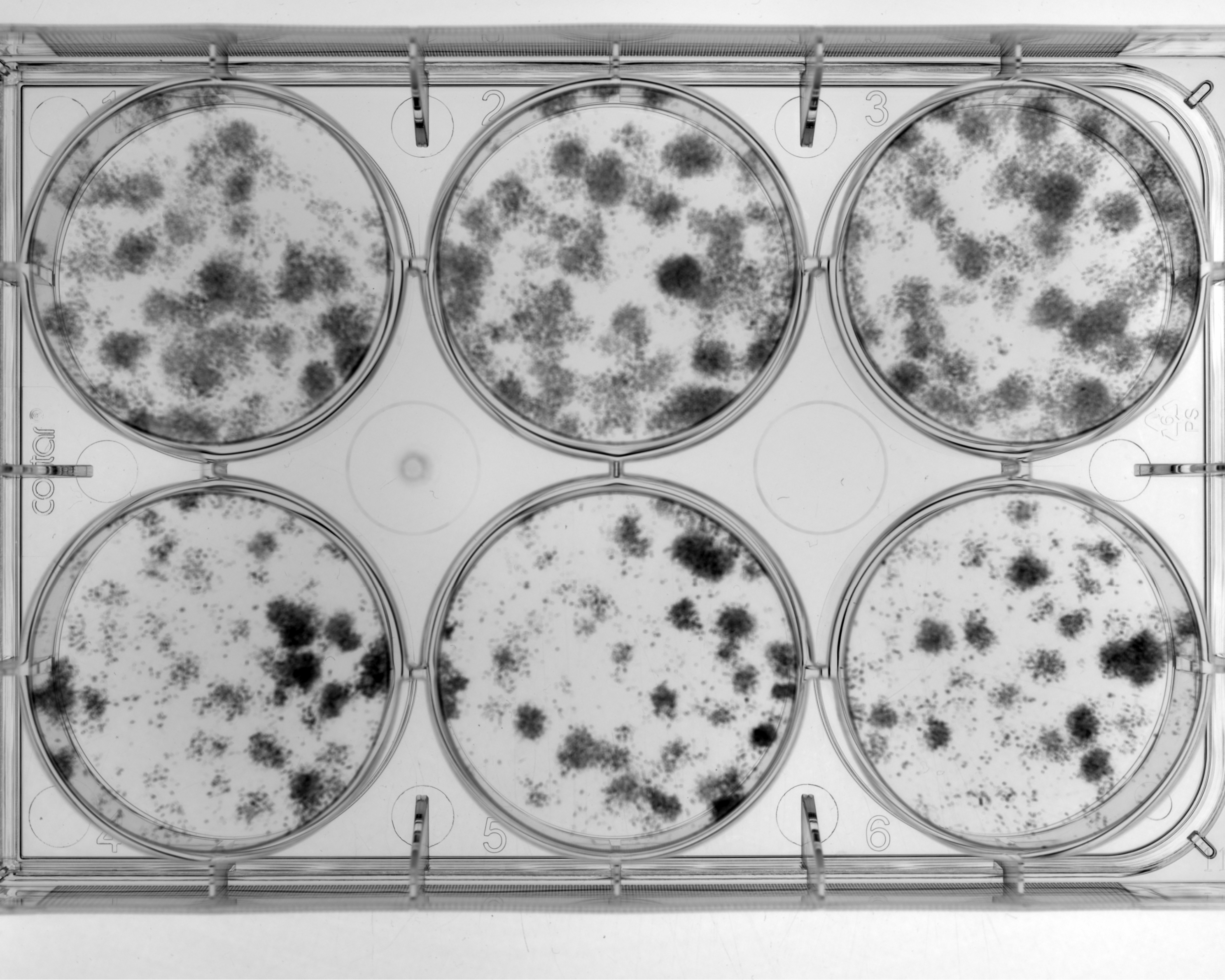

Supplement: Supplementary file 10 — Source data Fig. 3 [file 44318_2026_742_MOESM10_ESM.zip › FIgure 3/3E/CAKI-1/CAKI-1_2.tif]

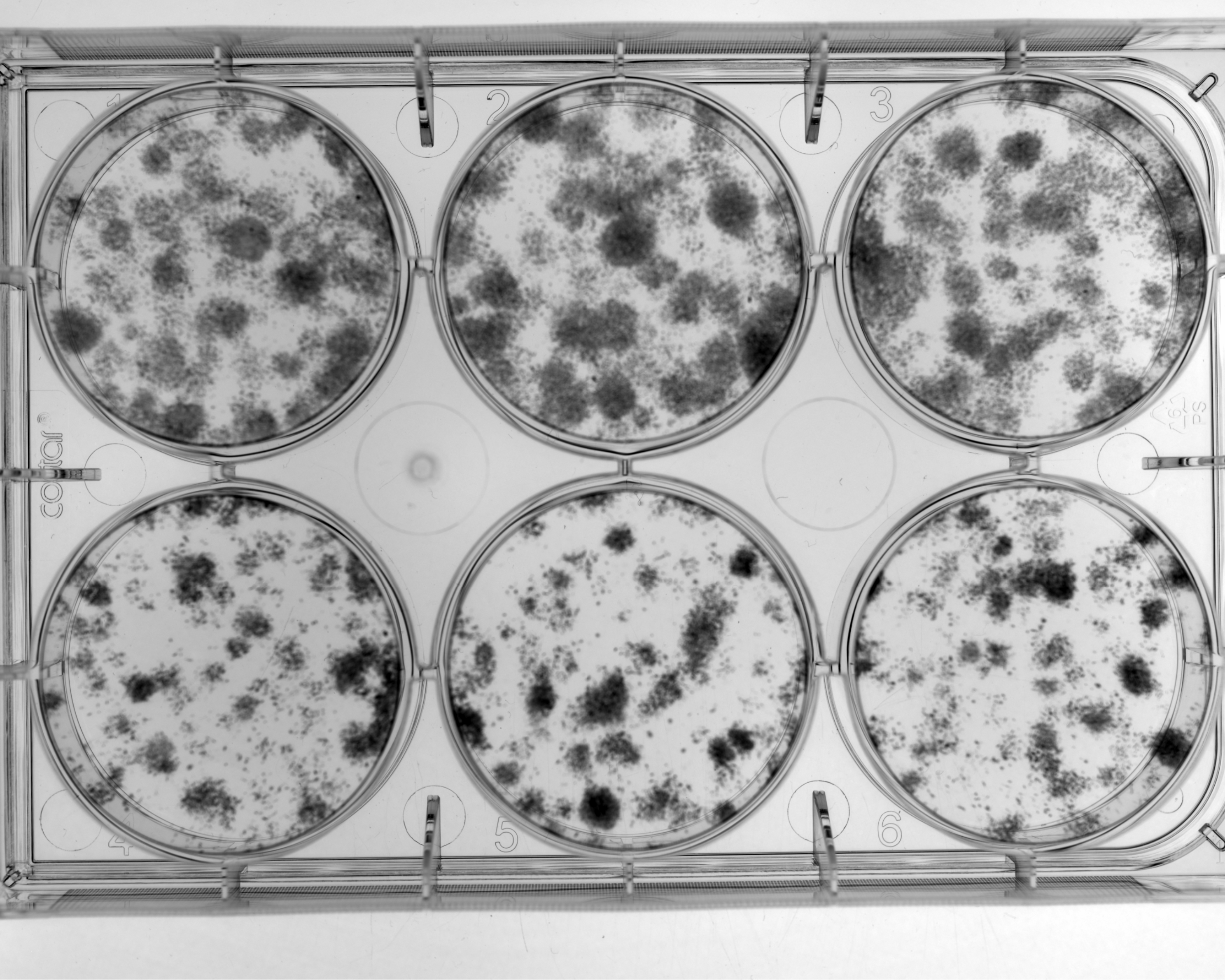

Supplement: Supplementary file 10 — Source data Fig. 3 [file 44318_2026_742_MOESM10_ESM.zip › FIgure 3/3E/CAKI-1/CAKI-1_3.tif]

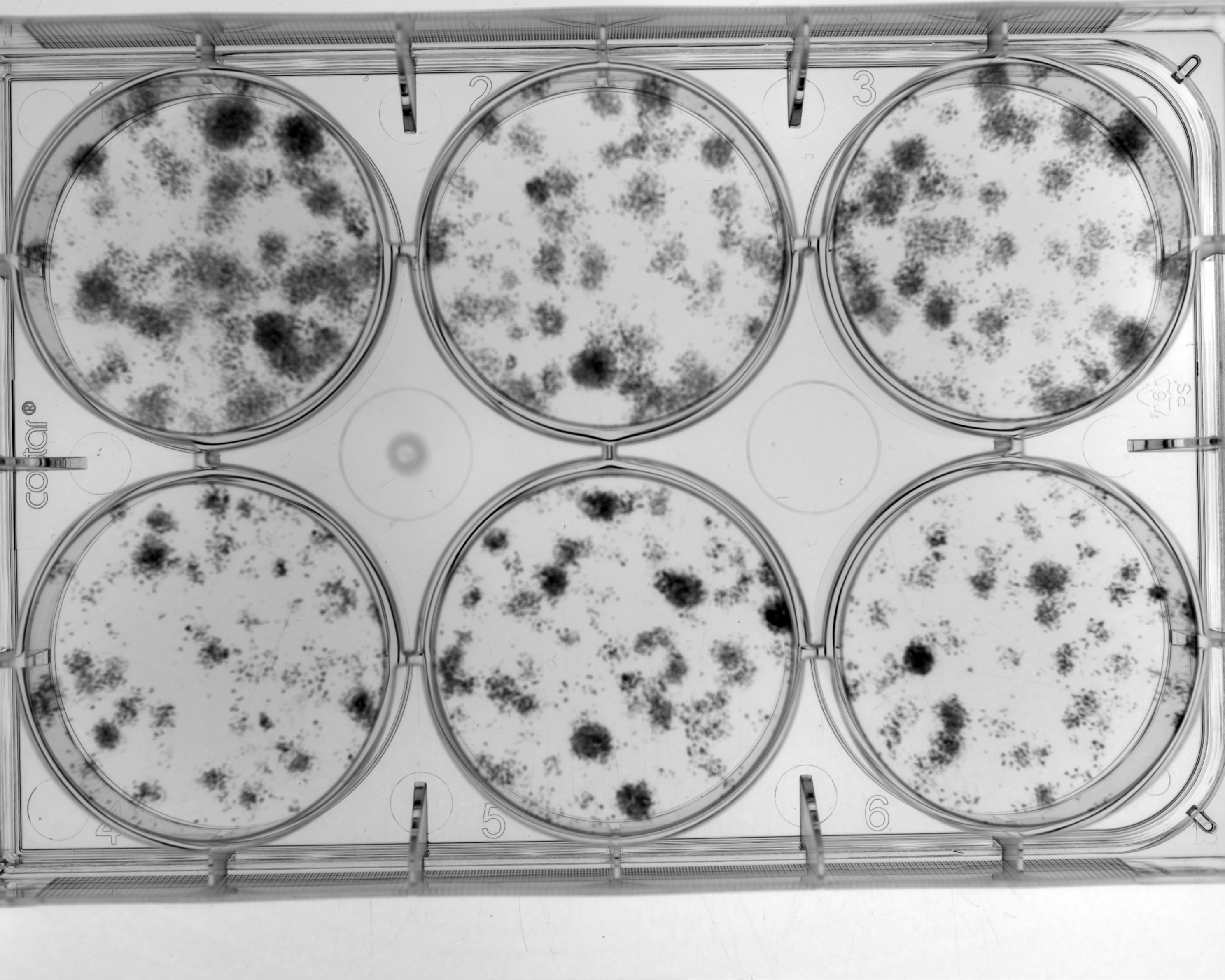

Supplement: Supplementary file 10 — Source data Fig. 3 [file 44318_2026_742_MOESM10_ESM.zip › FIgure 3/3E/CAKI-1/CAKI-1_1.tif]

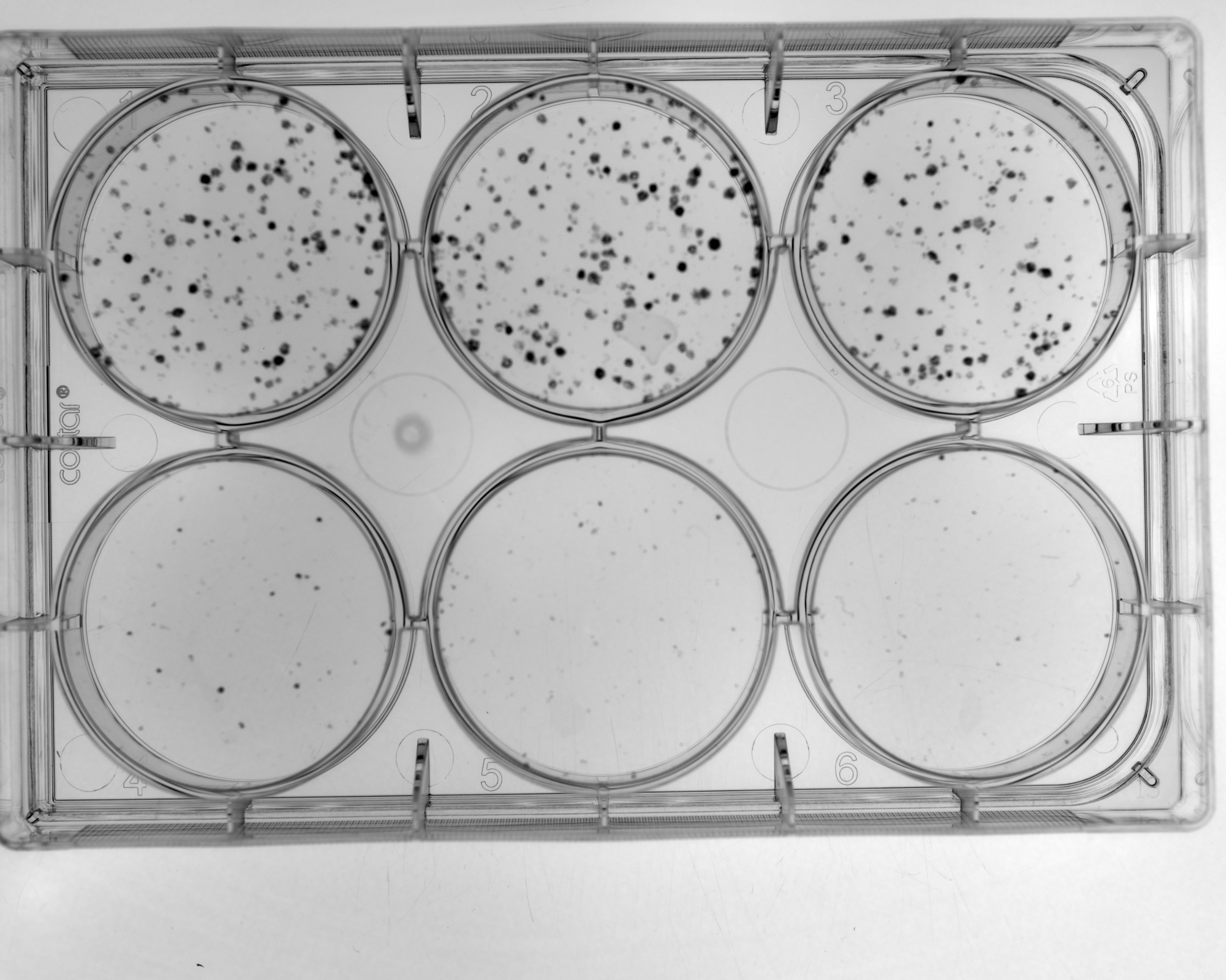

Supplement: Supplementary file 10 — Source data Fig. 3 [file 44318_2026_742_MOESM10_ESM.zip › FIgure 3/3E/SK-MEL-2/SK-MEL-2_3.tif]

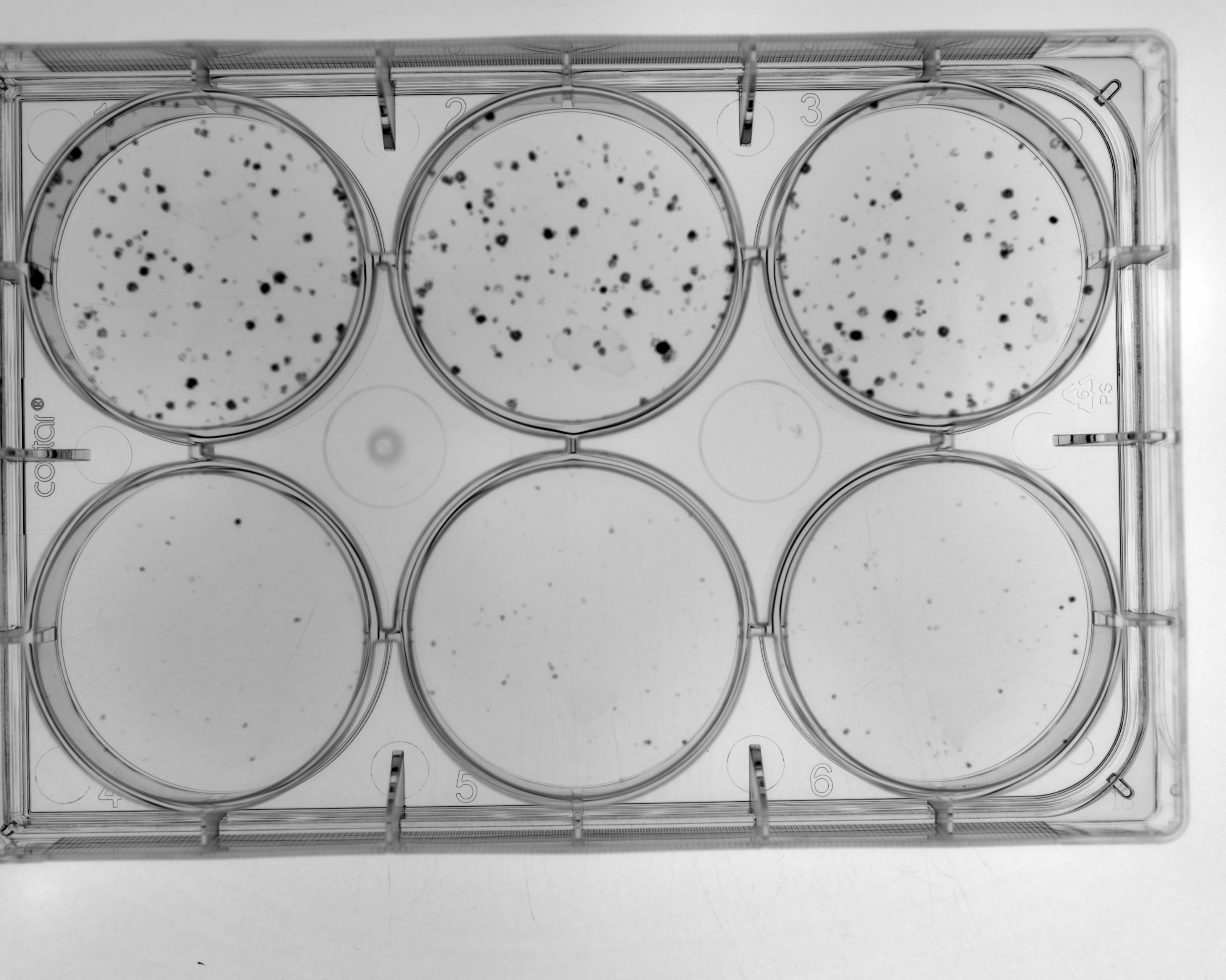

Supplement: Supplementary file 10 — Source data Fig. 3 [file 44318_2026_742_MOESM10_ESM.zip › FIgure 3/3E/SK-MEL-2/SK-MEL-2_2.tif]

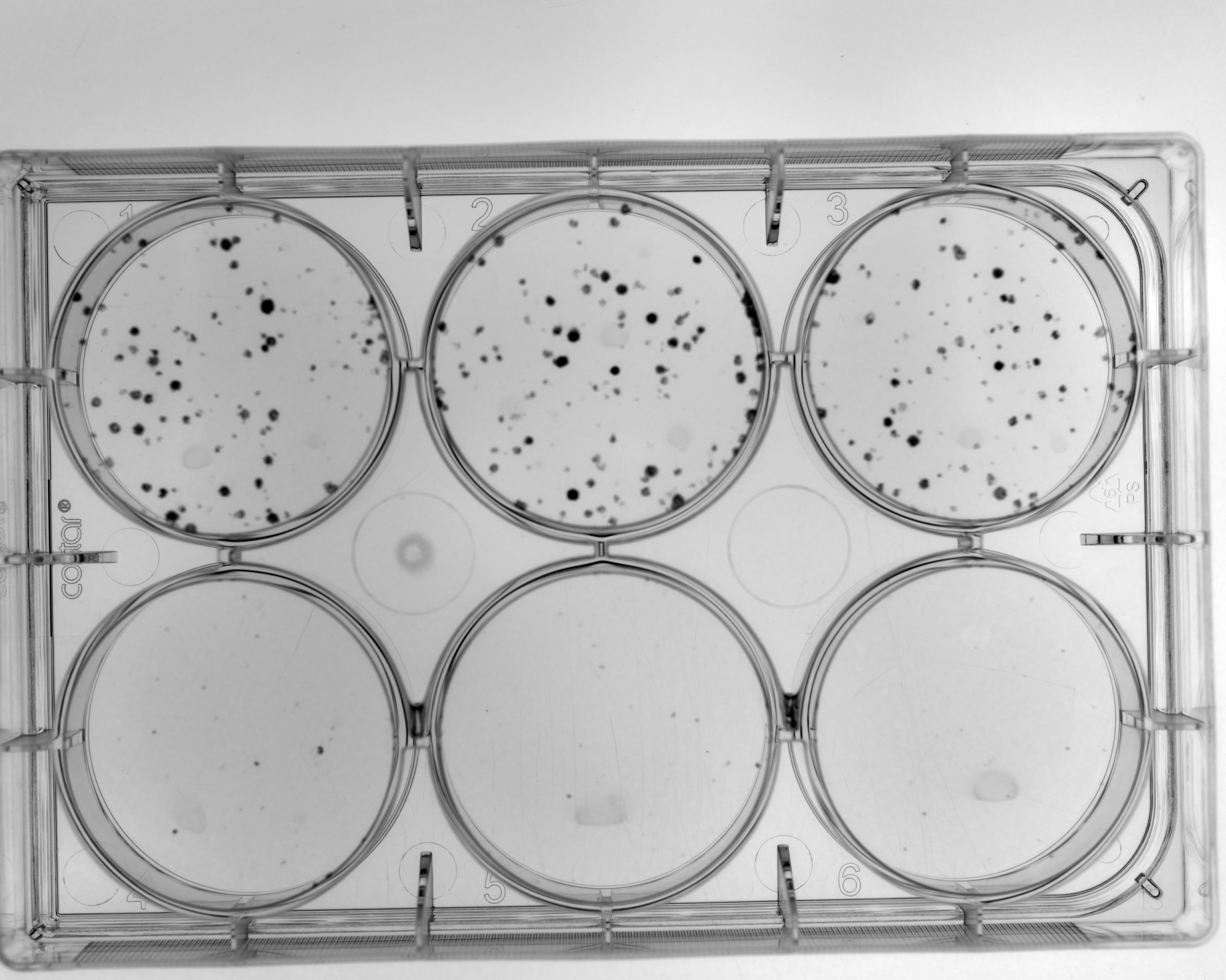

Supplement: Supplementary file 10 — Source data Fig. 3 [file 44318_2026_742_MOESM10_ESM.zip › FIgure 3/3E/SK-MEL-2/SK-MEL-2_1.tif]

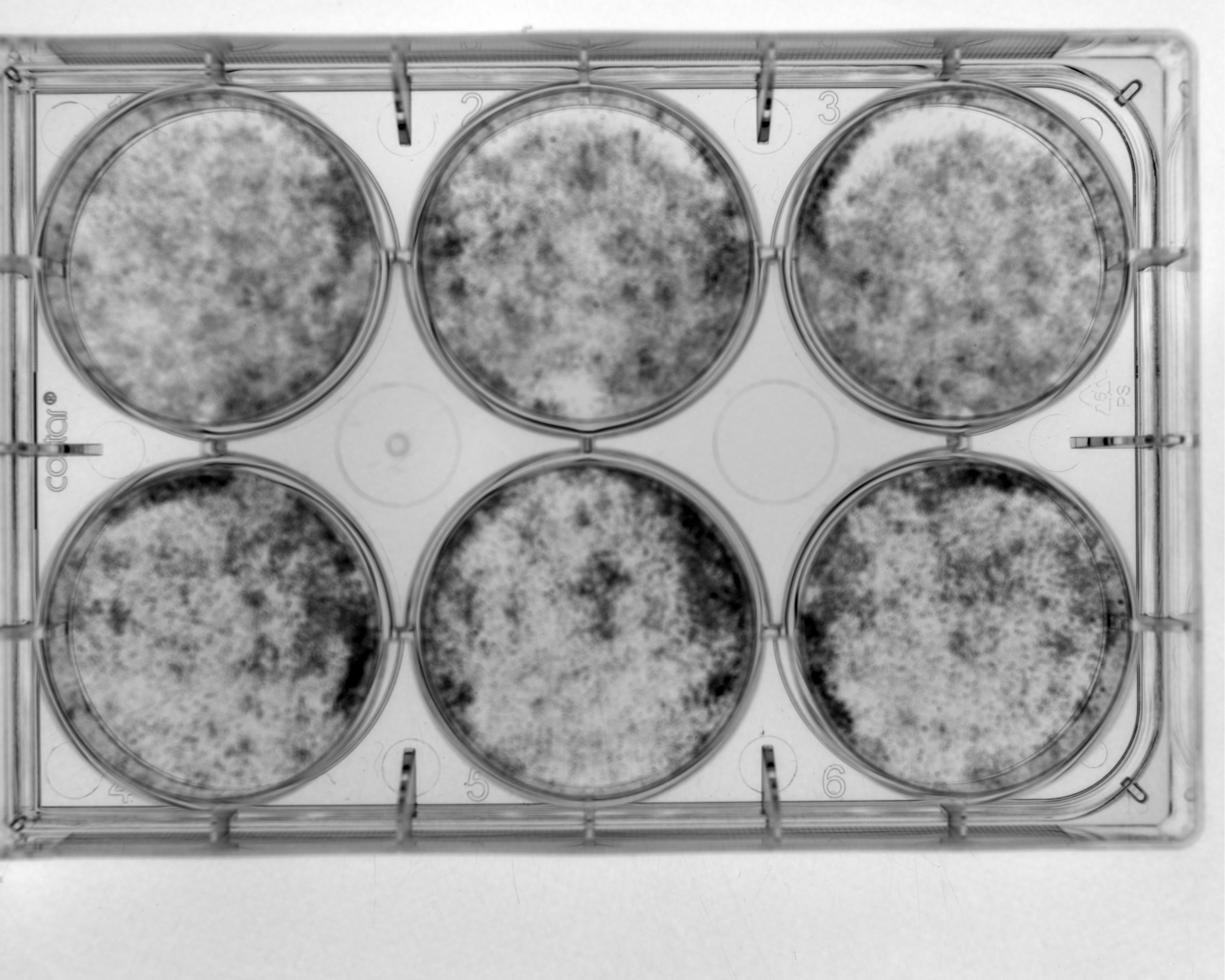

Supplement: Supplementary file 10 — Source data Fig. 3 [file 44318_2026_742_MOESM10_ESM.zip › FIgure 3/3E/HDF/HDF_3.tif]

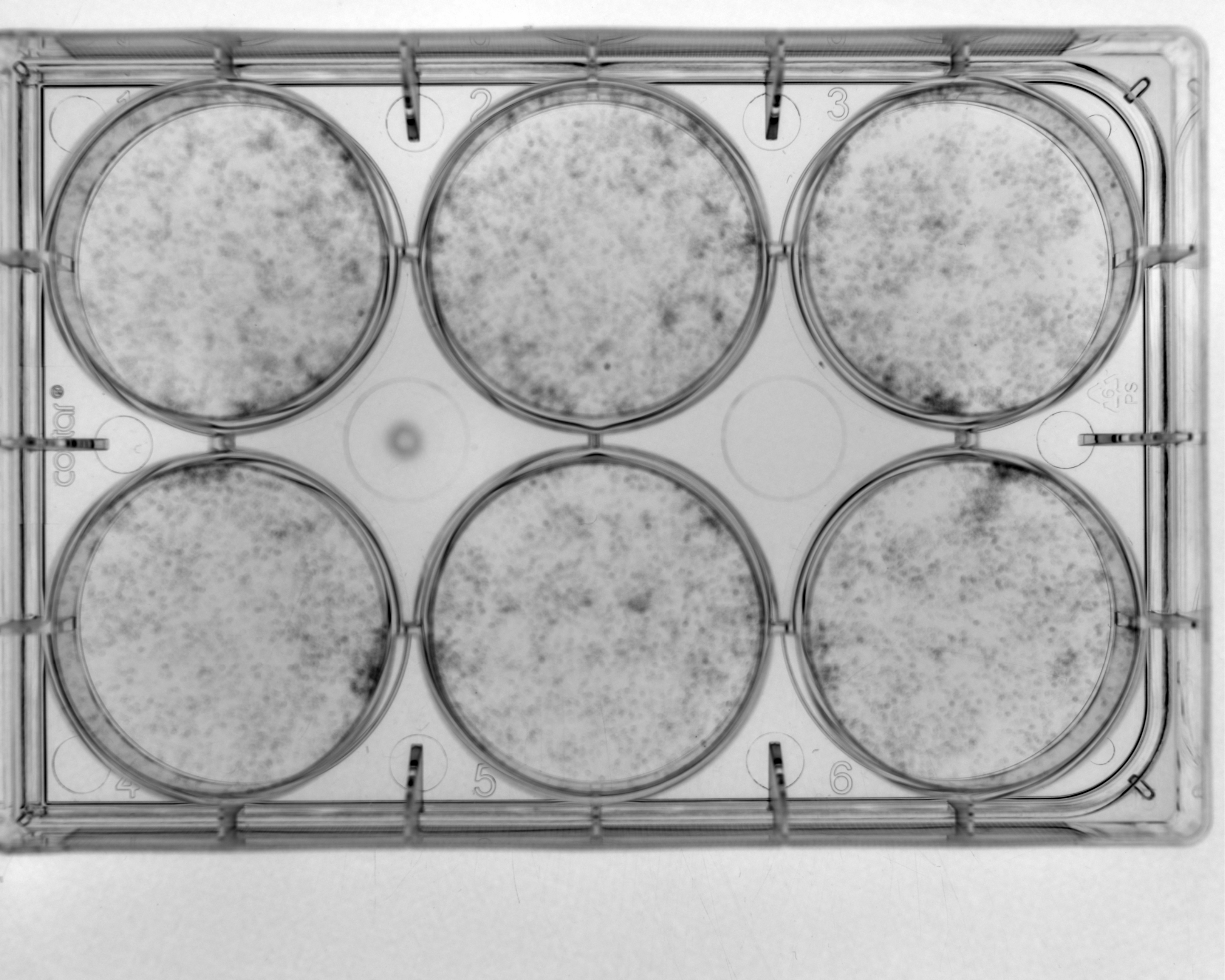

Supplement: Supplementary file 10 — Source data Fig. 3 [file 44318_2026_742_MOESM10_ESM.zip › FIgure 3/3E/HDF/HDF_2.tif]

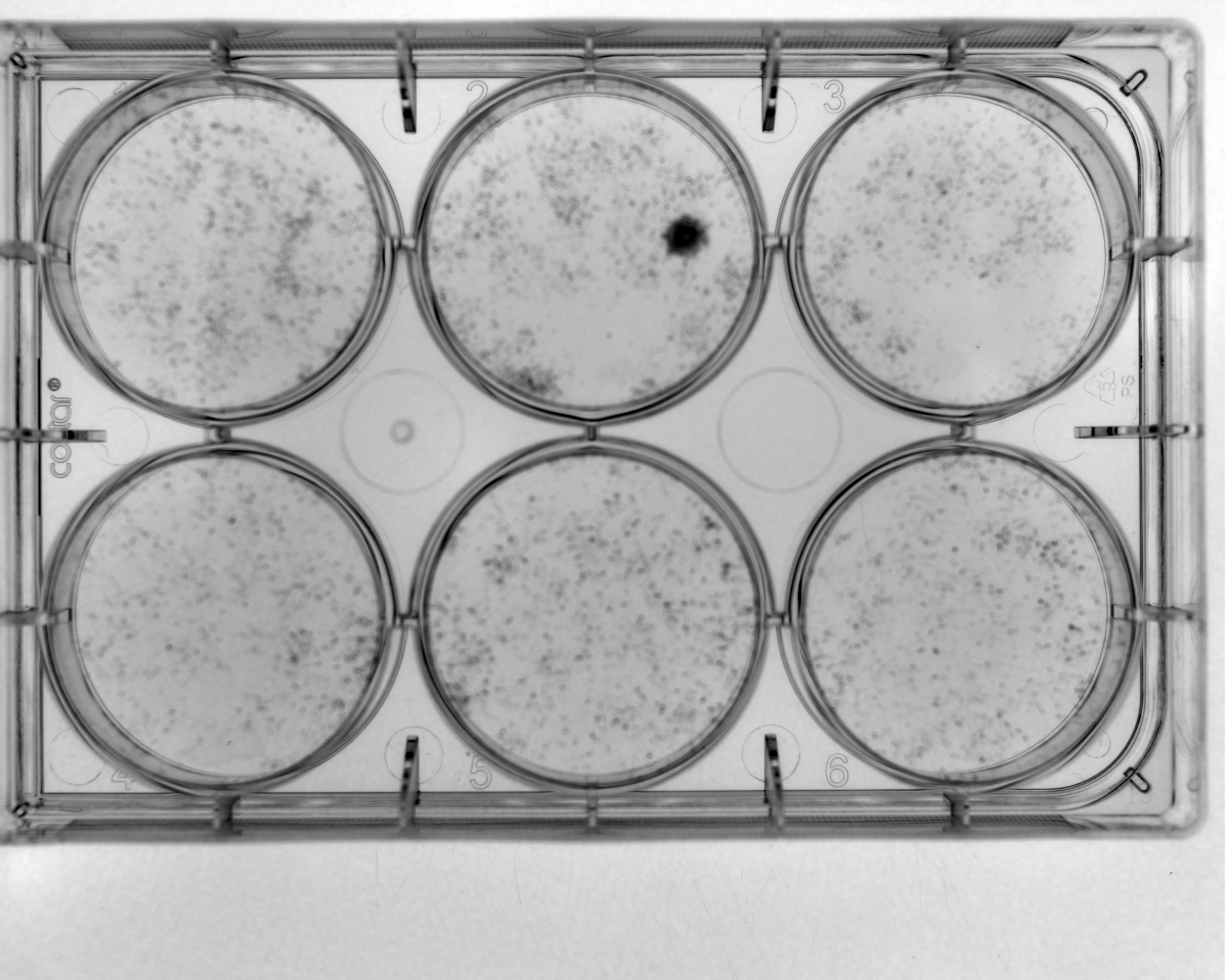

Supplement: Supplementary file 10 — Source data Fig. 3 [file 44318_2026_742_MOESM10_ESM.zip › FIgure 3/3E/HDF/HDF_1.tif]

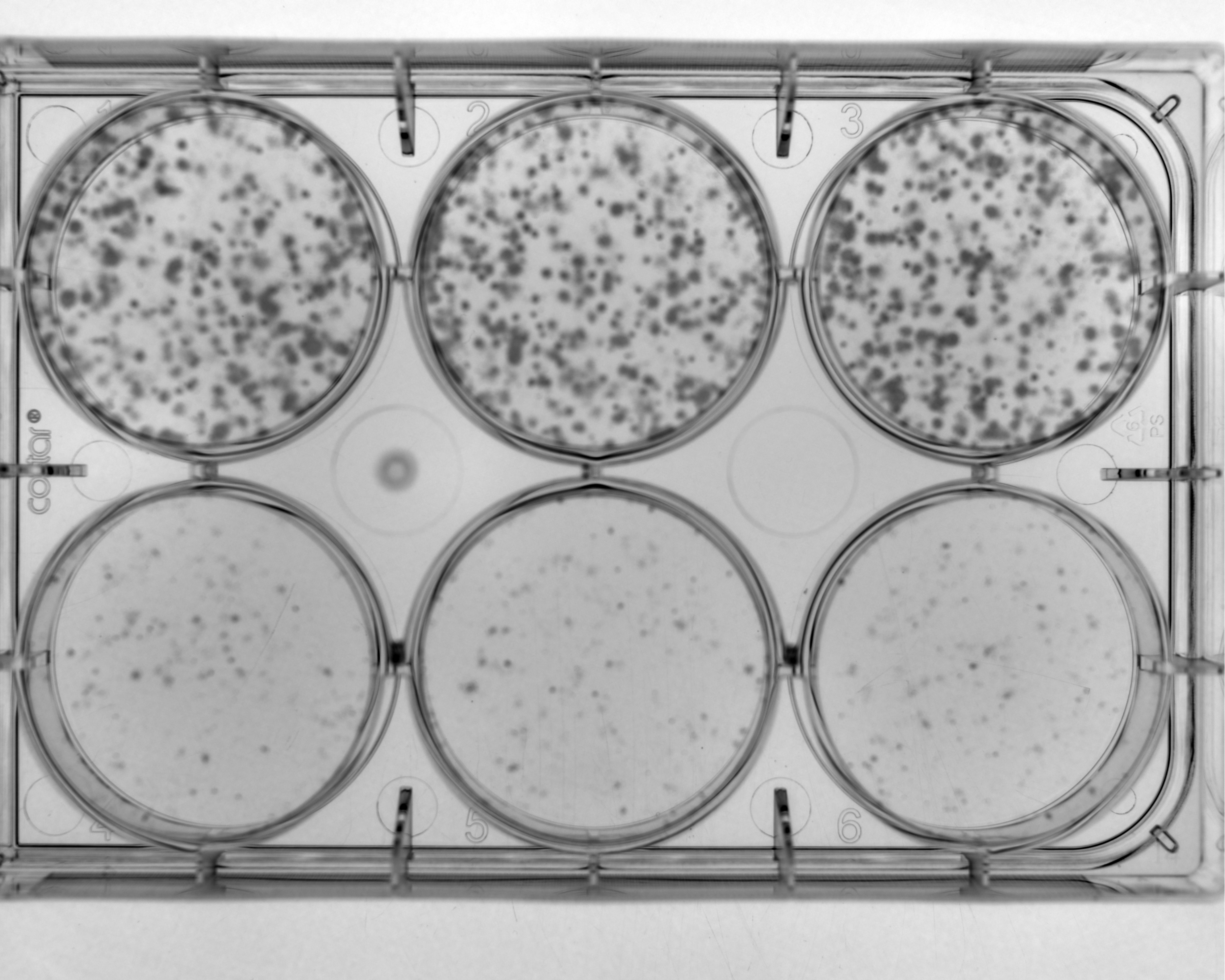

Supplement: Supplementary file 10 — Source data Fig. 3 [file 44318_2026_742_MOESM10_ESM.zip › FIgure 3/3E/MiaPaca2/MiaPaca2_1.tif]

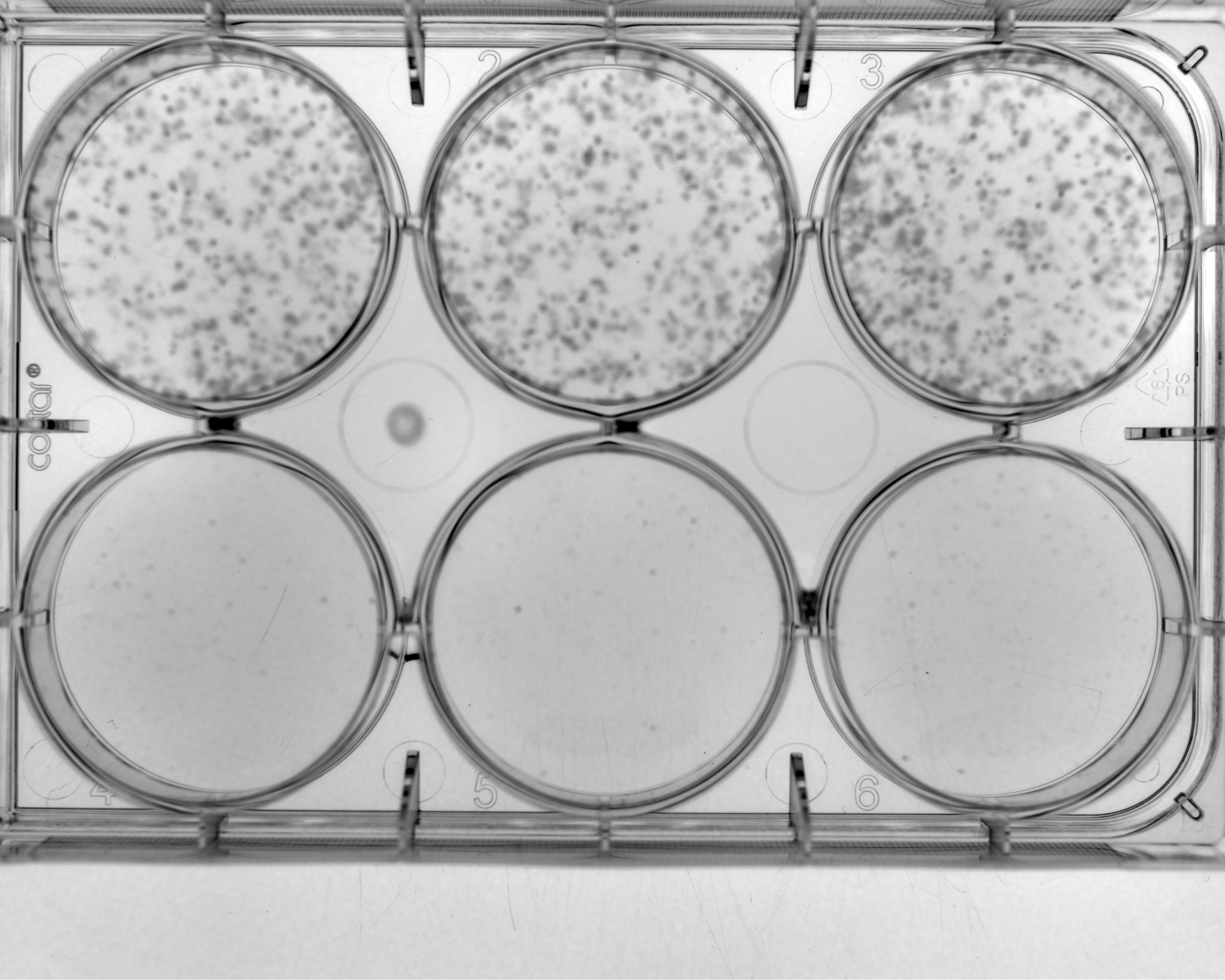

Supplement: Supplementary file 10 — Source data Fig. 3 [file 44318_2026_742_MOESM10_ESM.zip › FIgure 3/3E/MiaPaca2/MiaPaca2_2.tif]

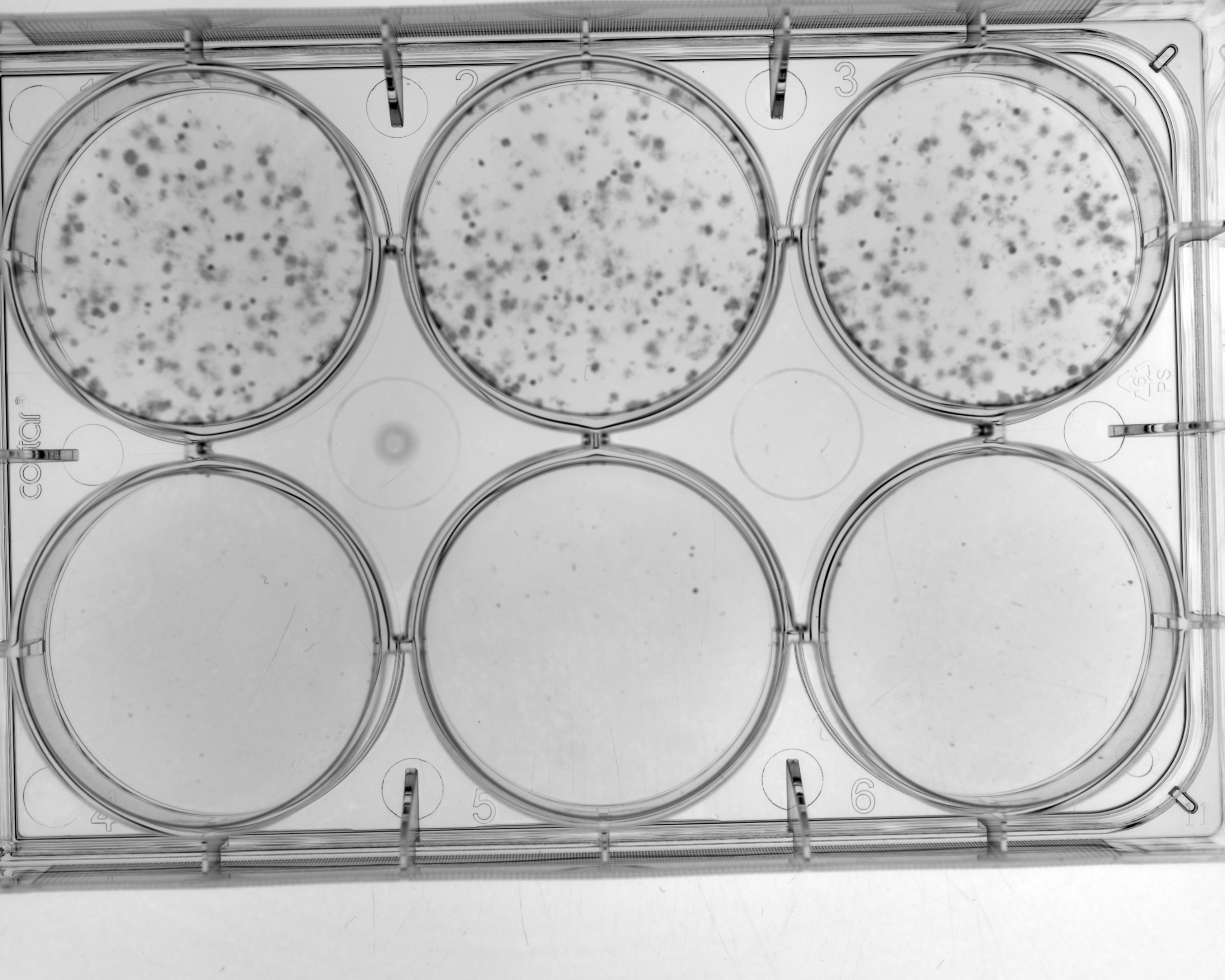

Supplement: Supplementary file 10 — Source data Fig. 3 [file 44318_2026_742_MOESM10_ESM.zip › FIgure 3/3E/MiaPaca2/MiaPaca2_3.tif]

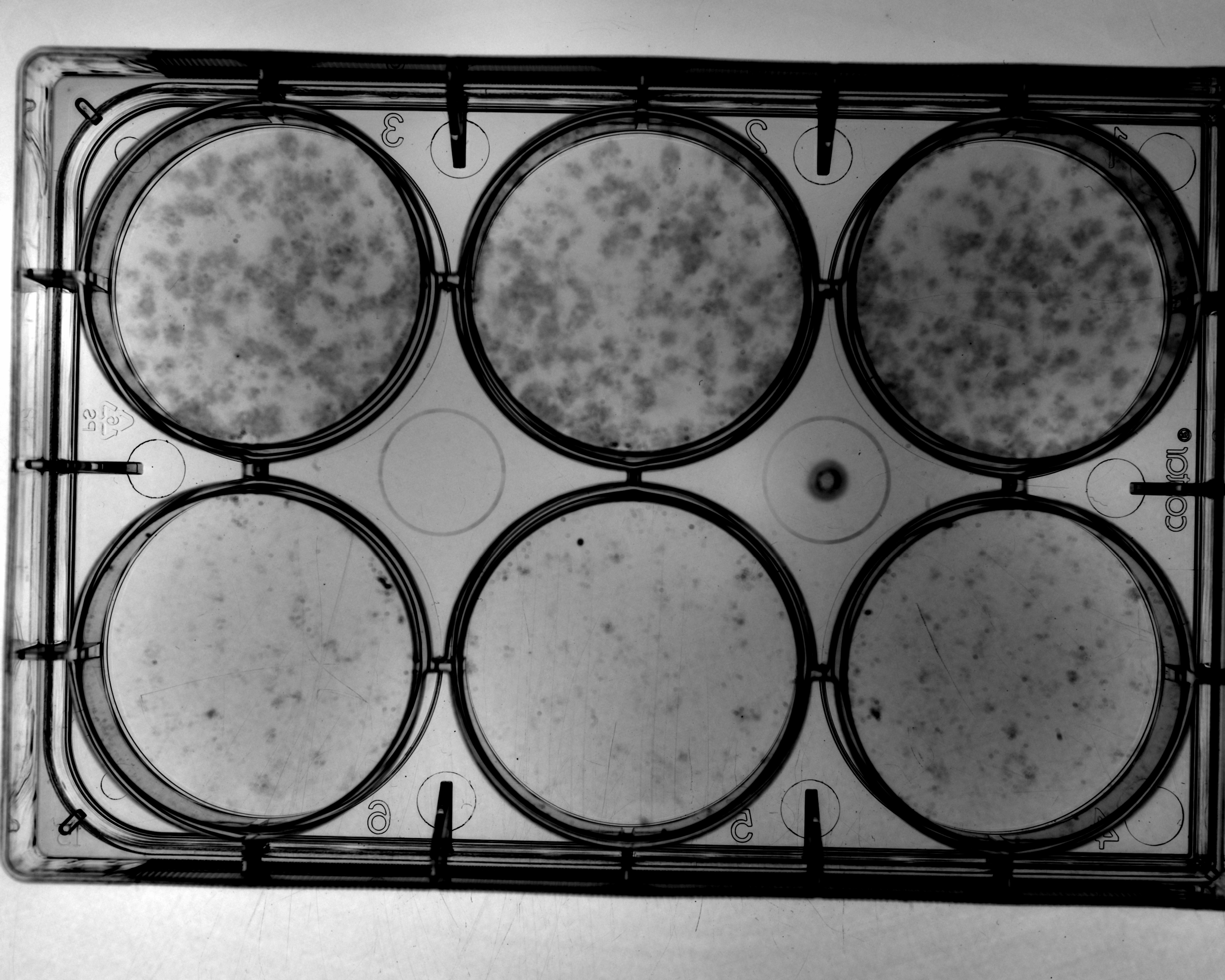

Supplement: Supplementary file 10 — Source data Fig. 3 [file 44318_2026_742_MOESM10_ESM.zip › FIgure 3/3E/UO-31/UO-31_1.tif]

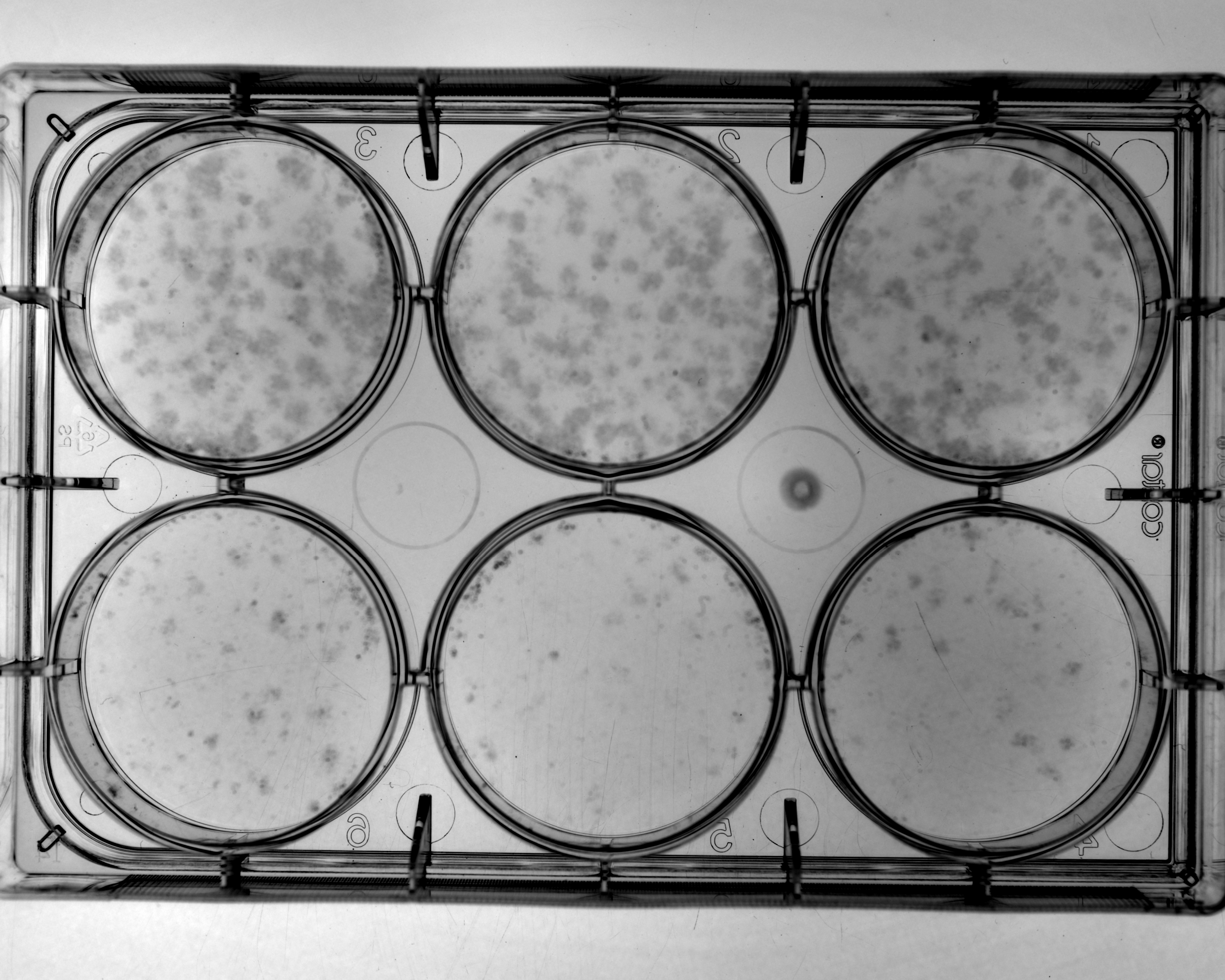

Supplement: Supplementary file 10 — Source data Fig. 3 [file 44318_2026_742_MOESM10_ESM.zip › FIgure 3/3E/UO-31/UO-31_2.tif]

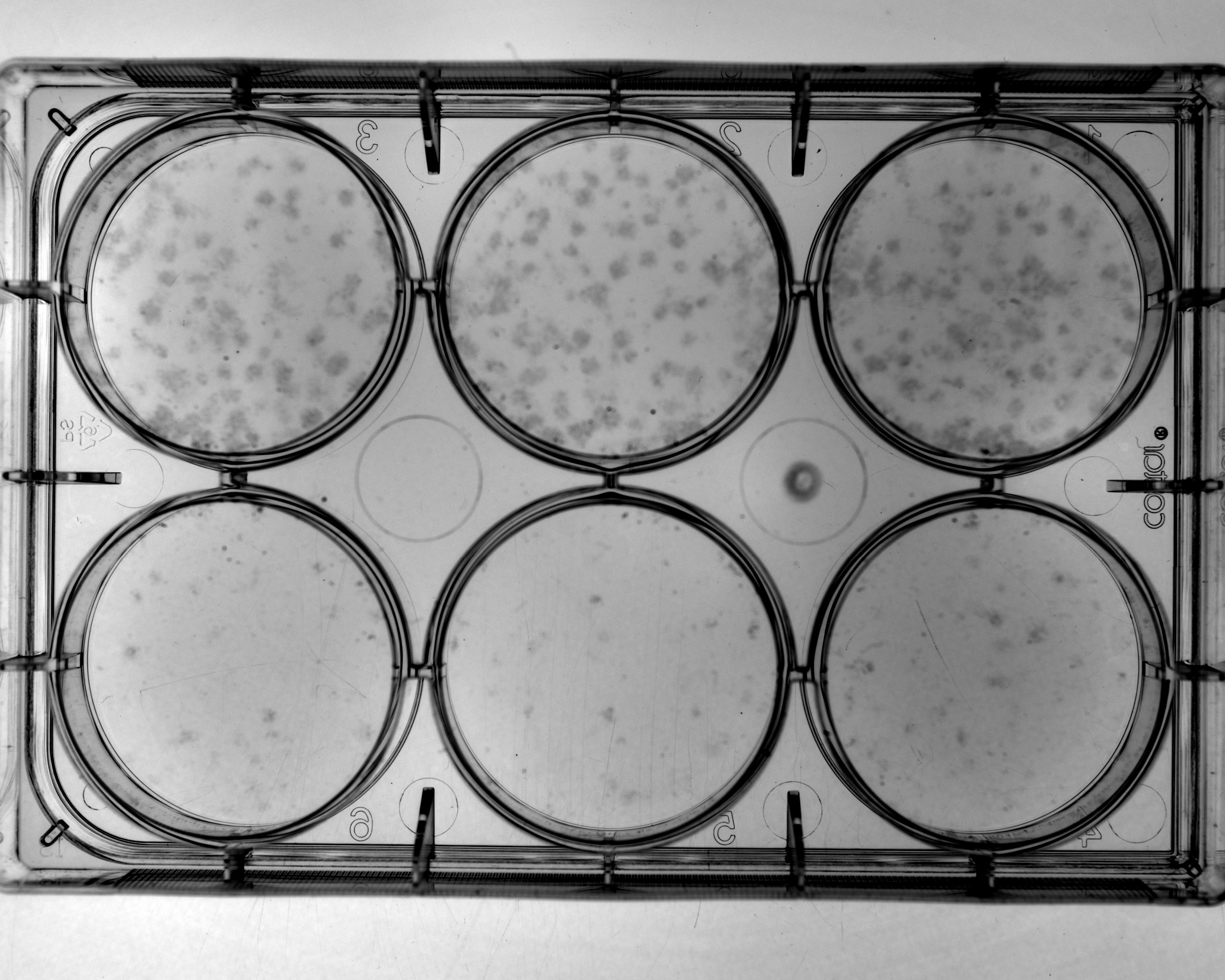

Supplement: Supplementary file 10 — Source data Fig. 3 [file 44318_2026_742_MOESM10_ESM.zip › FIgure 3/3E/UO-31/UO-31_3.tif]

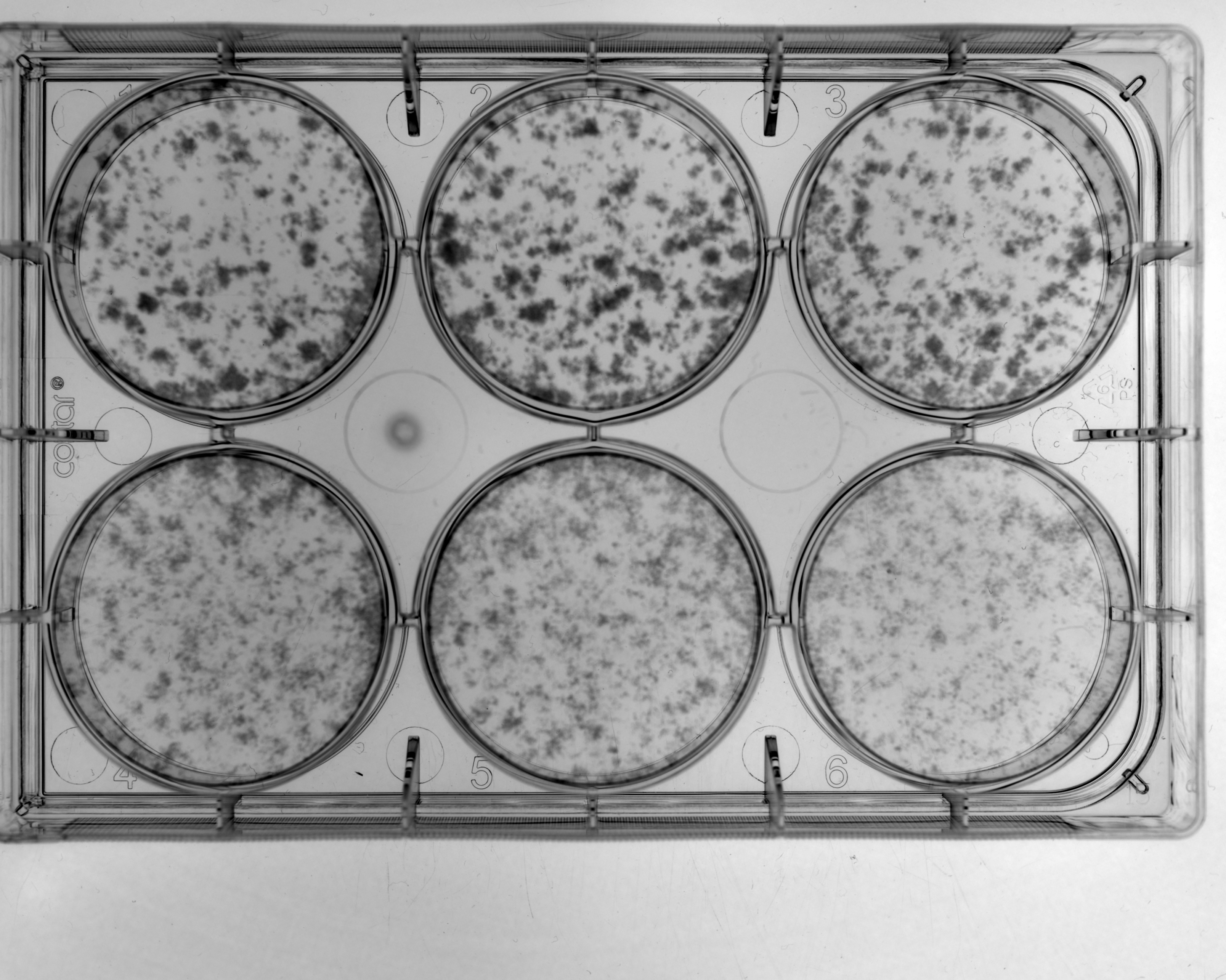

Supplement: Supplementary file 10 — Source data Fig. 3 [file 44318_2026_742_MOESM10_ESM.zip › FIgure 3/3E/HUVEC/HUVEC_2.tif]

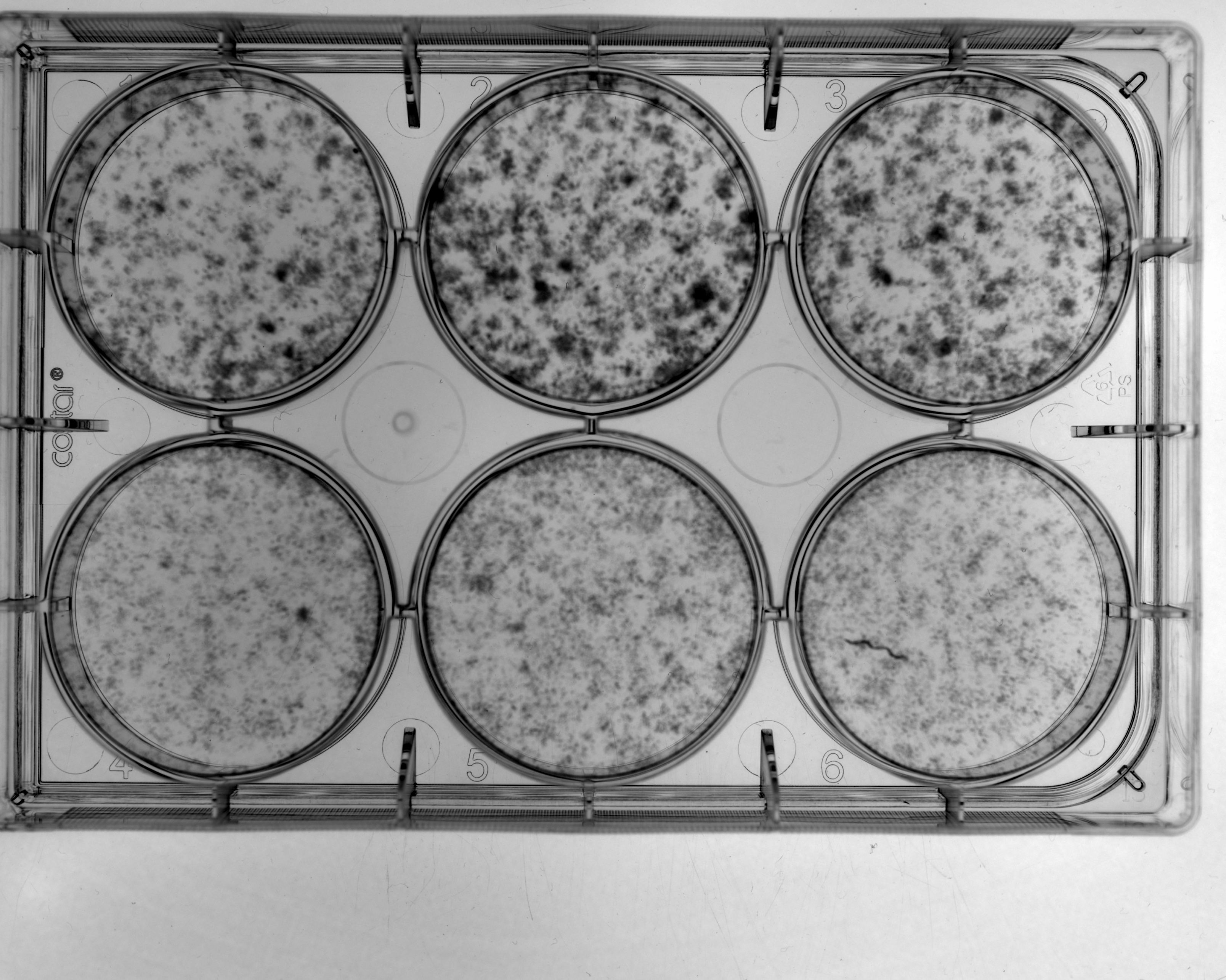

Supplement: Supplementary file 10 — Source data Fig. 3 [file 44318_2026_742_MOESM10_ESM.zip › FIgure 3/3E/HUVEC/HUVEC_3.tif]

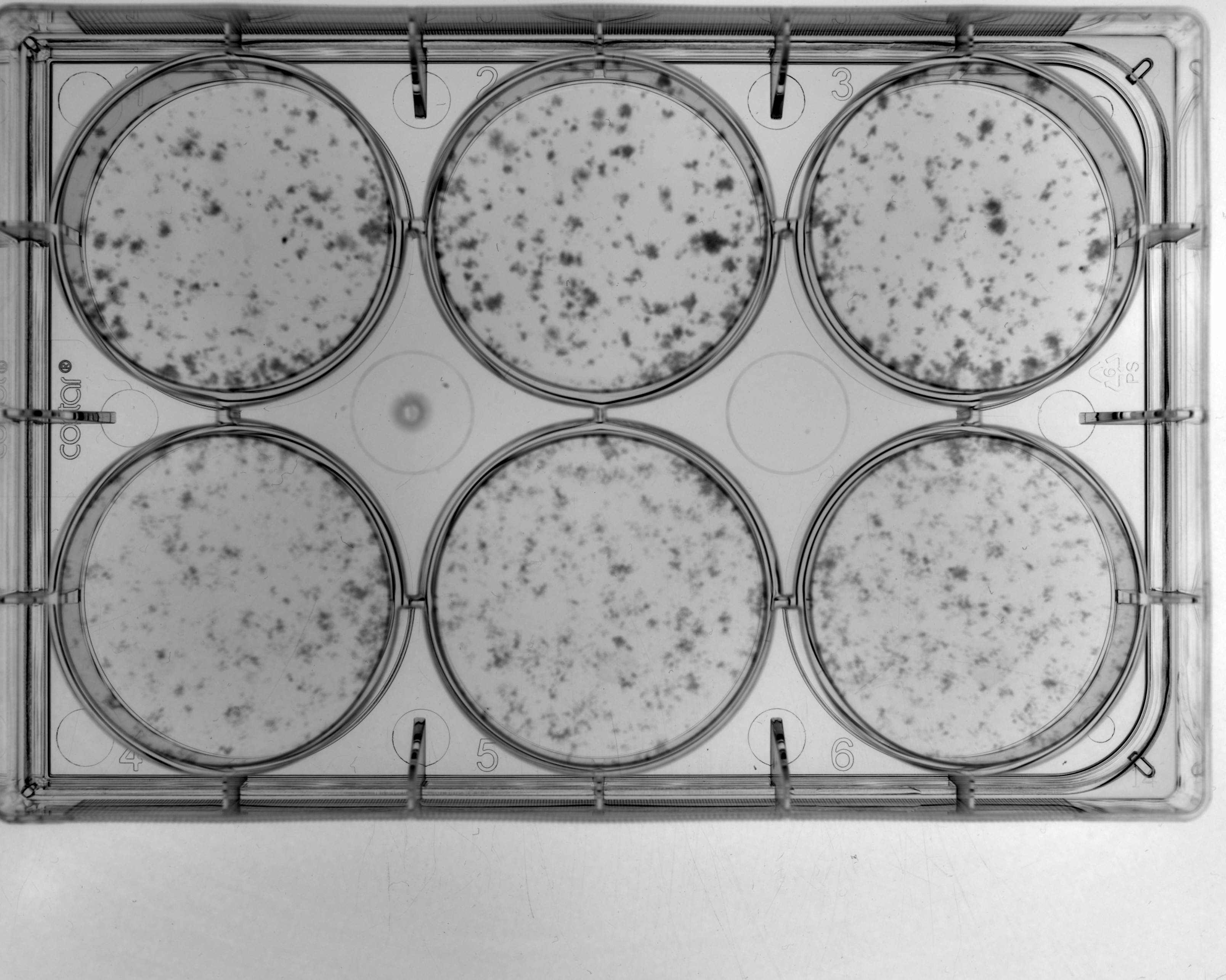

Supplement: Supplementary file 10 — Source data Fig. 3 [file 44318_2026_742_MOESM10_ESM.zip › FIgure 3/3E/HUVEC/HUVEC_1.tif]

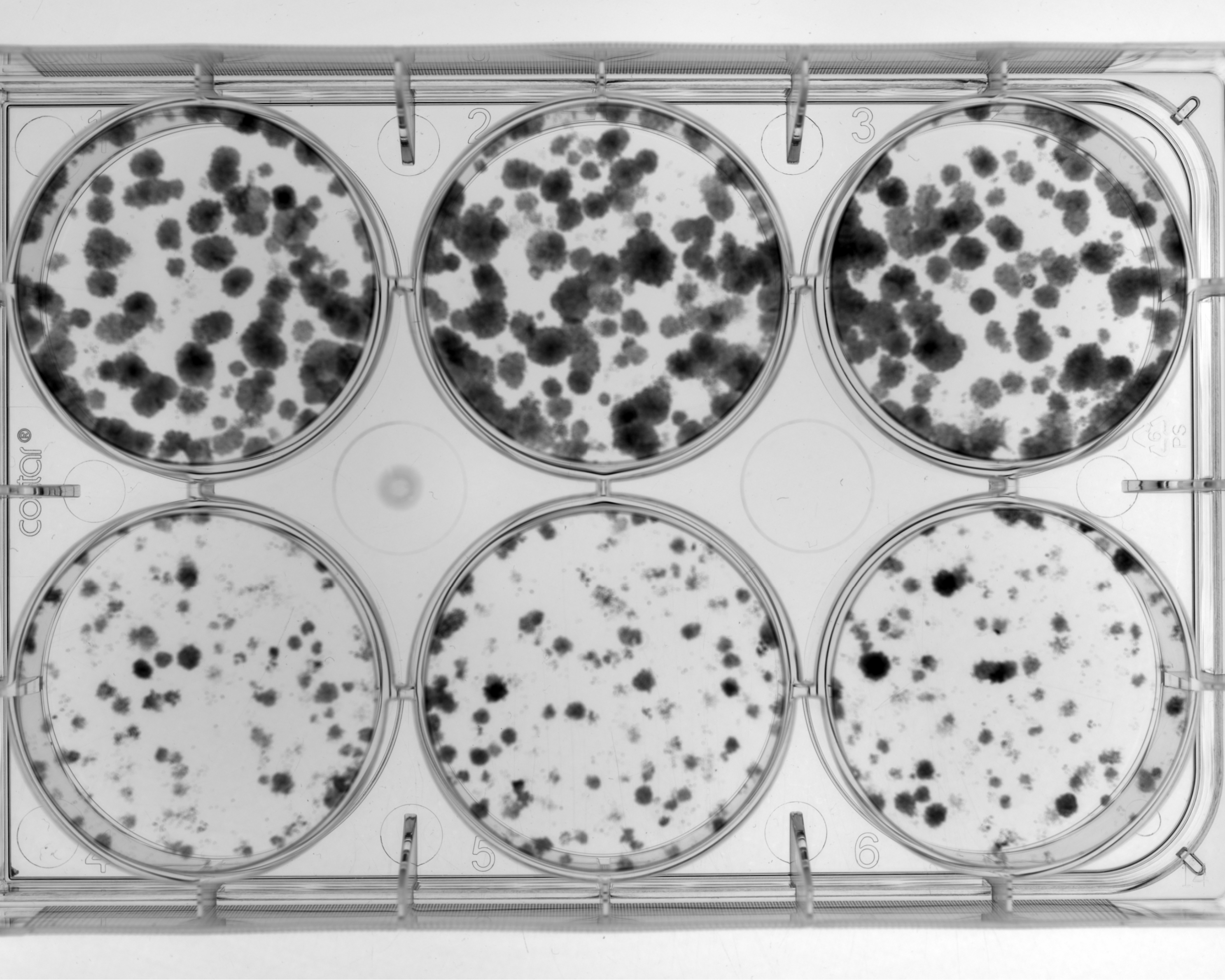

Supplement: Supplementary file 10 — Source data Fig. 3 [file 44318_2026_742_MOESM10_ESM.zip › FIgure 3/3E/A498/A498_2.tif]

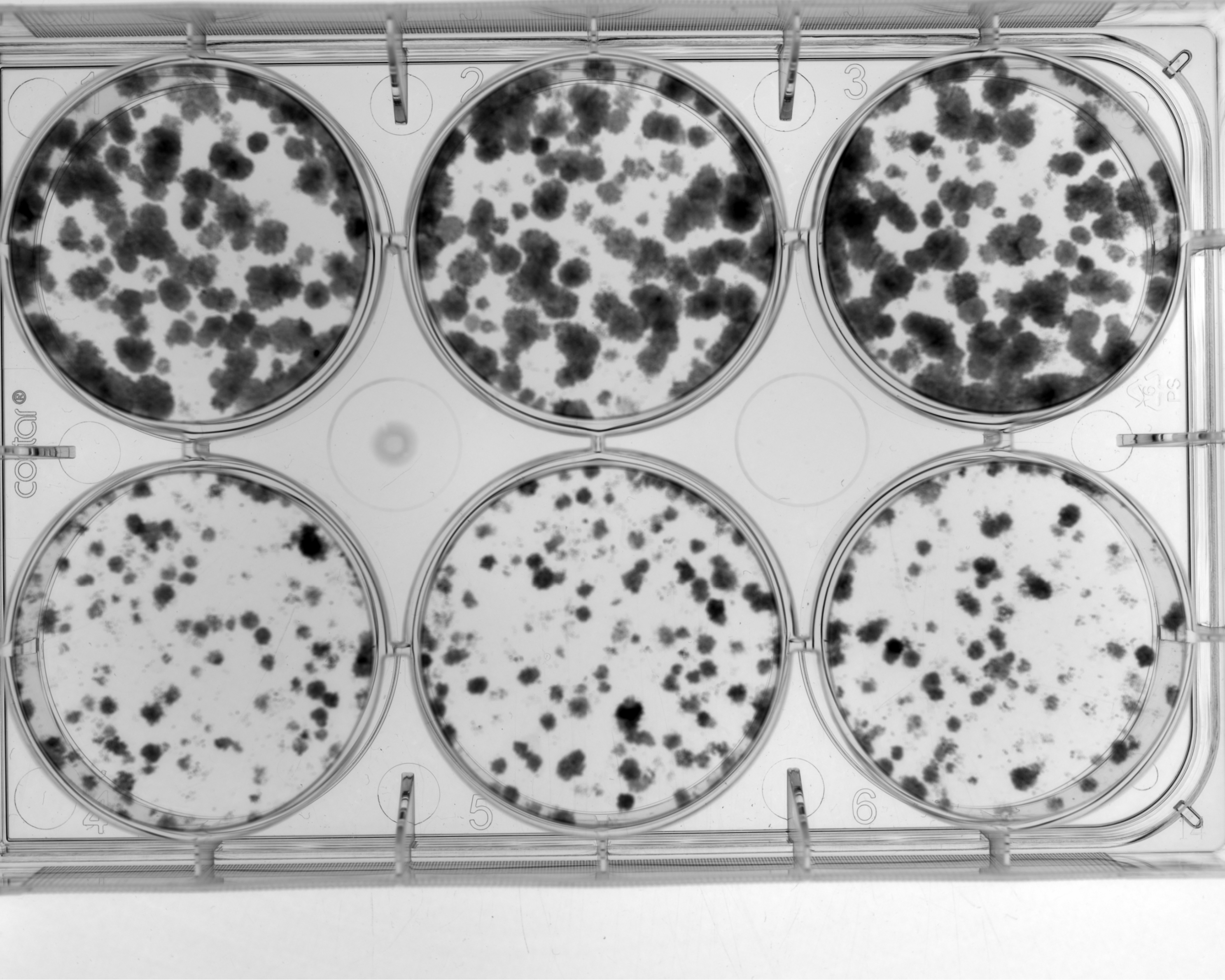

Supplement: Supplementary file 10 — Source data Fig. 3 [file 44318_2026_742_MOESM10_ESM.zip › FIgure 3/3E/A498/A498_3.tif]

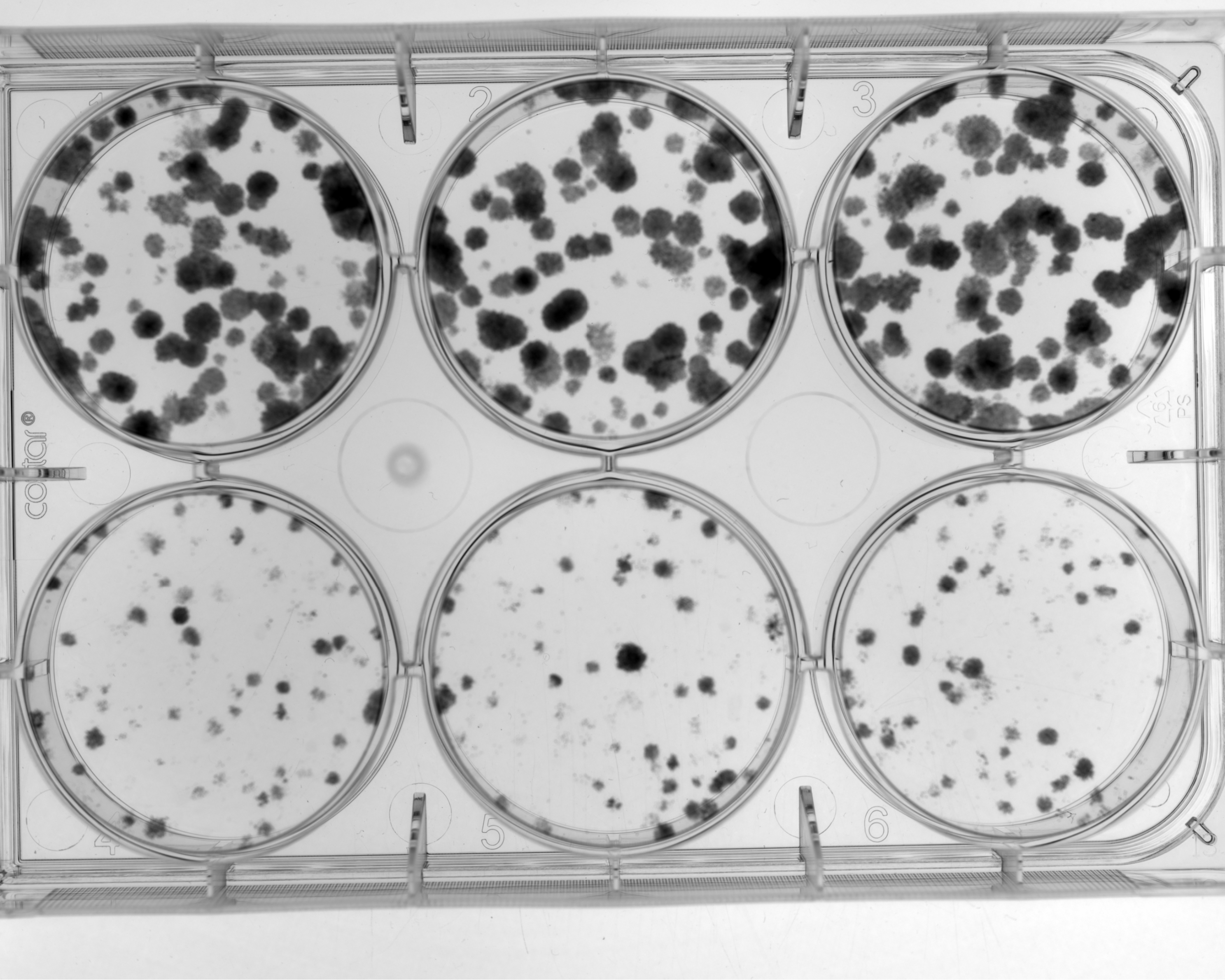

Supplement: Supplementary file 10 — Source data Fig. 3 [file 44318_2026_742_MOESM10_ESM.zip › FIgure 3/3E/A498/A498_1.tif]

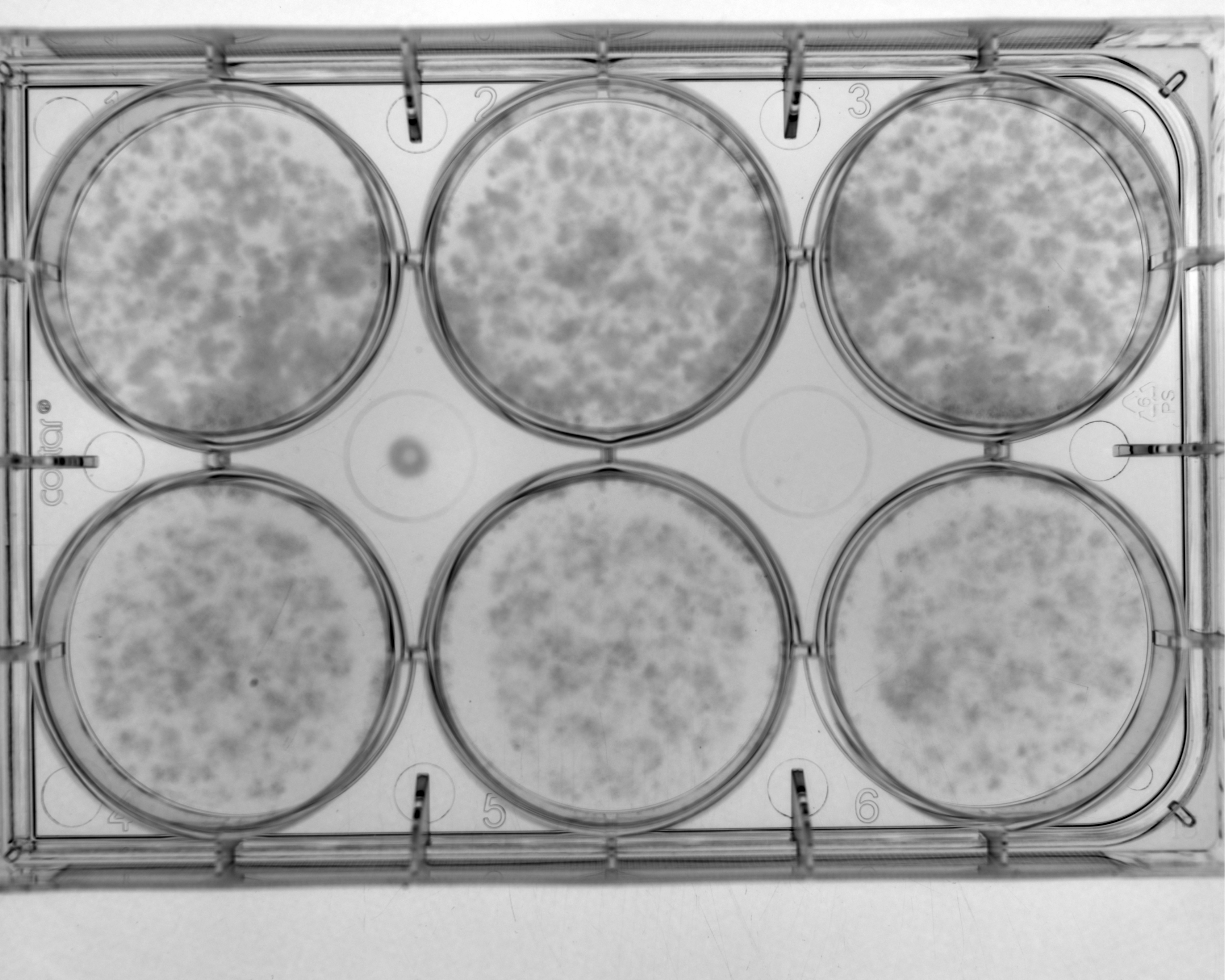

Supplement: Supplementary file 10 — Source data Fig. 3 [file 44318_2026_742_MOESM10_ESM.zip › FIgure 3/3E/HMEC-1/HMEC-1_1.tif]

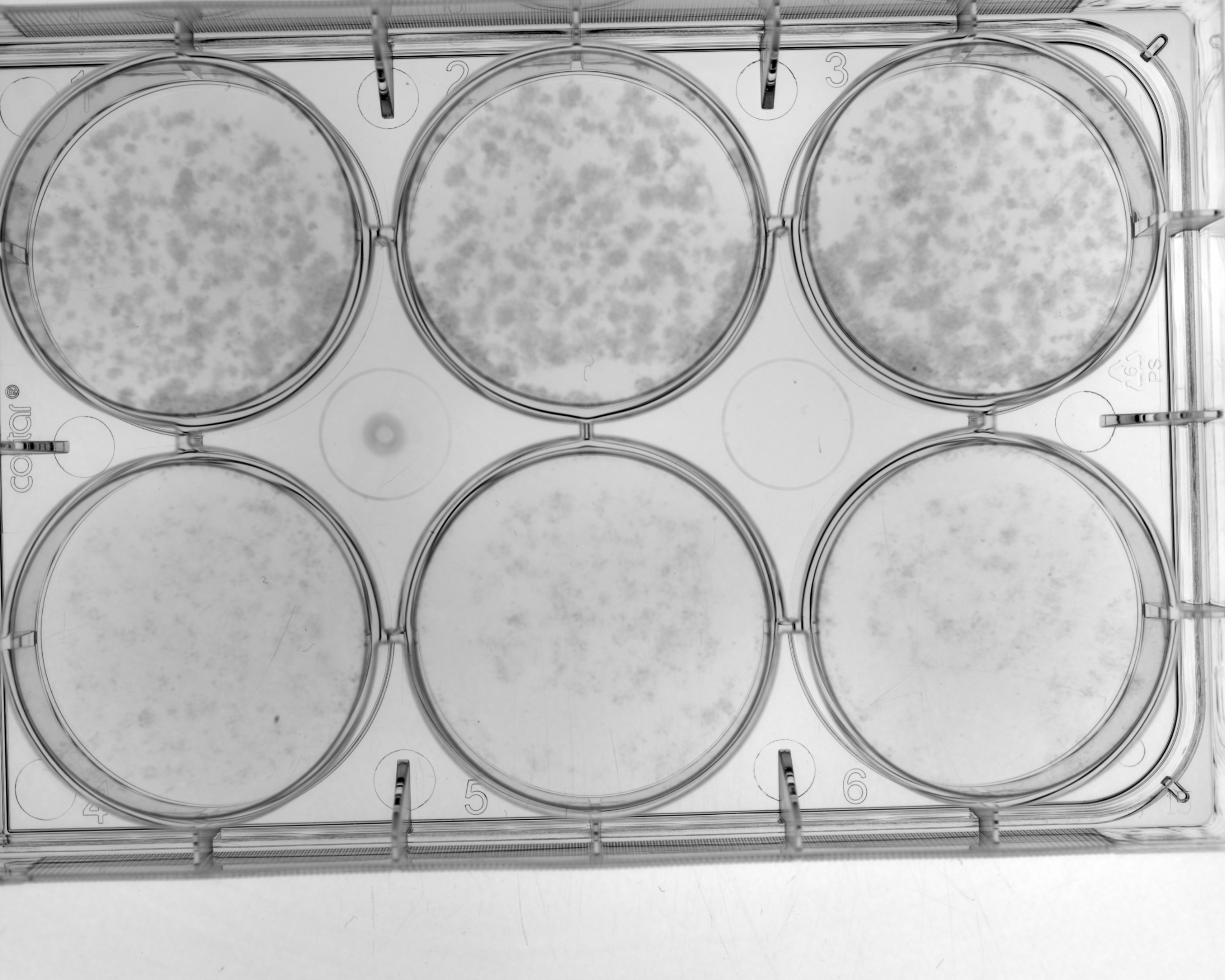

Supplement: Supplementary file 10 — Source data Fig. 3 [file 44318_2026_742_MOESM10_ESM.zip › FIgure 3/3E/HMEC-1/HMEC-1_3.tif]

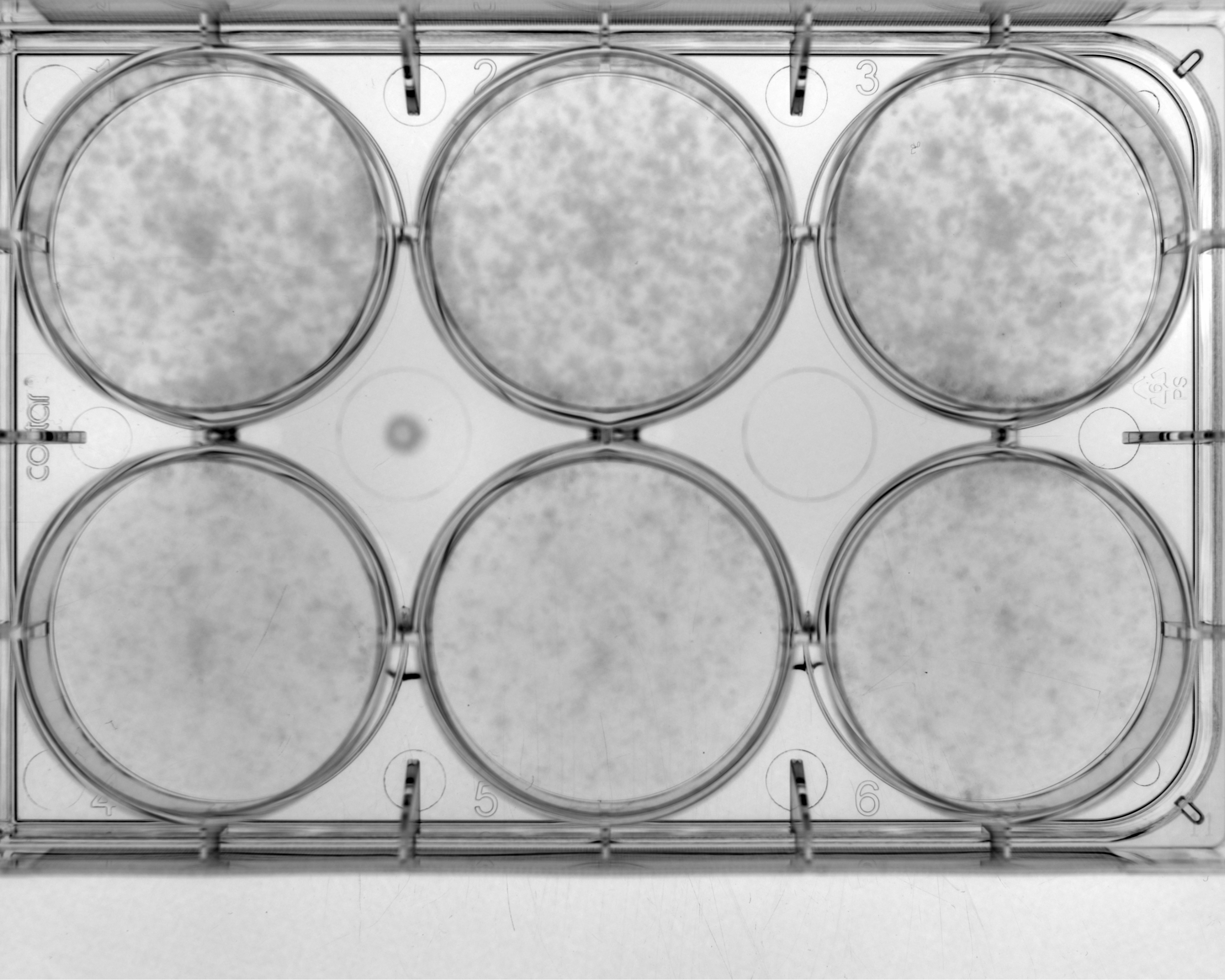

Supplement: Supplementary file 10 — Source data Fig. 3 [file 44318_2026_742_MOESM10_ESM.zip › FIgure 3/3E/HMEC-1/HMEC-1_2.tif]

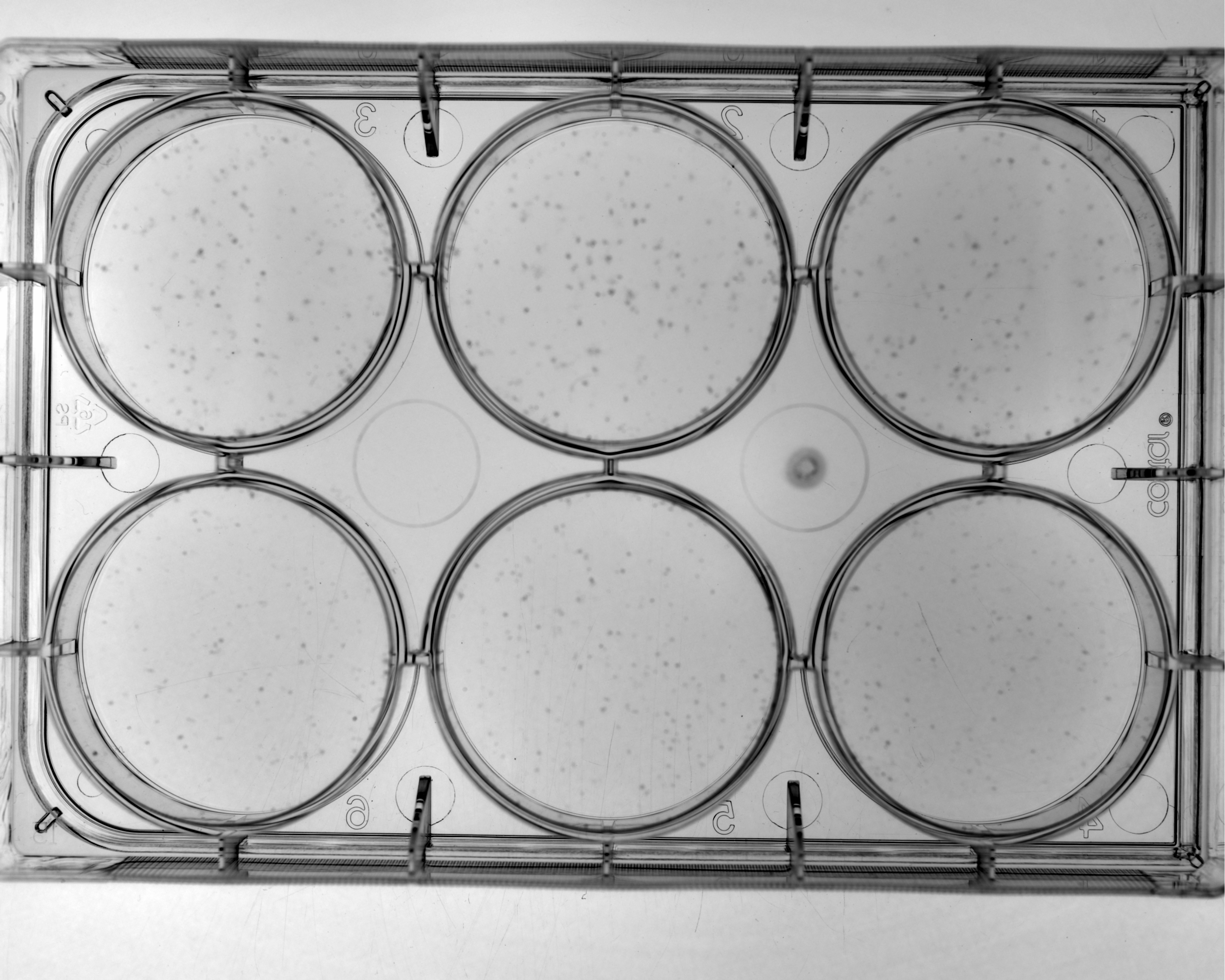

Supplement: Supplementary file 10 — Source data Fig. 3 [file 44318_2026_742_MOESM10_ESM.zip › FIgure 3/3E/A549/A549_1.tif]

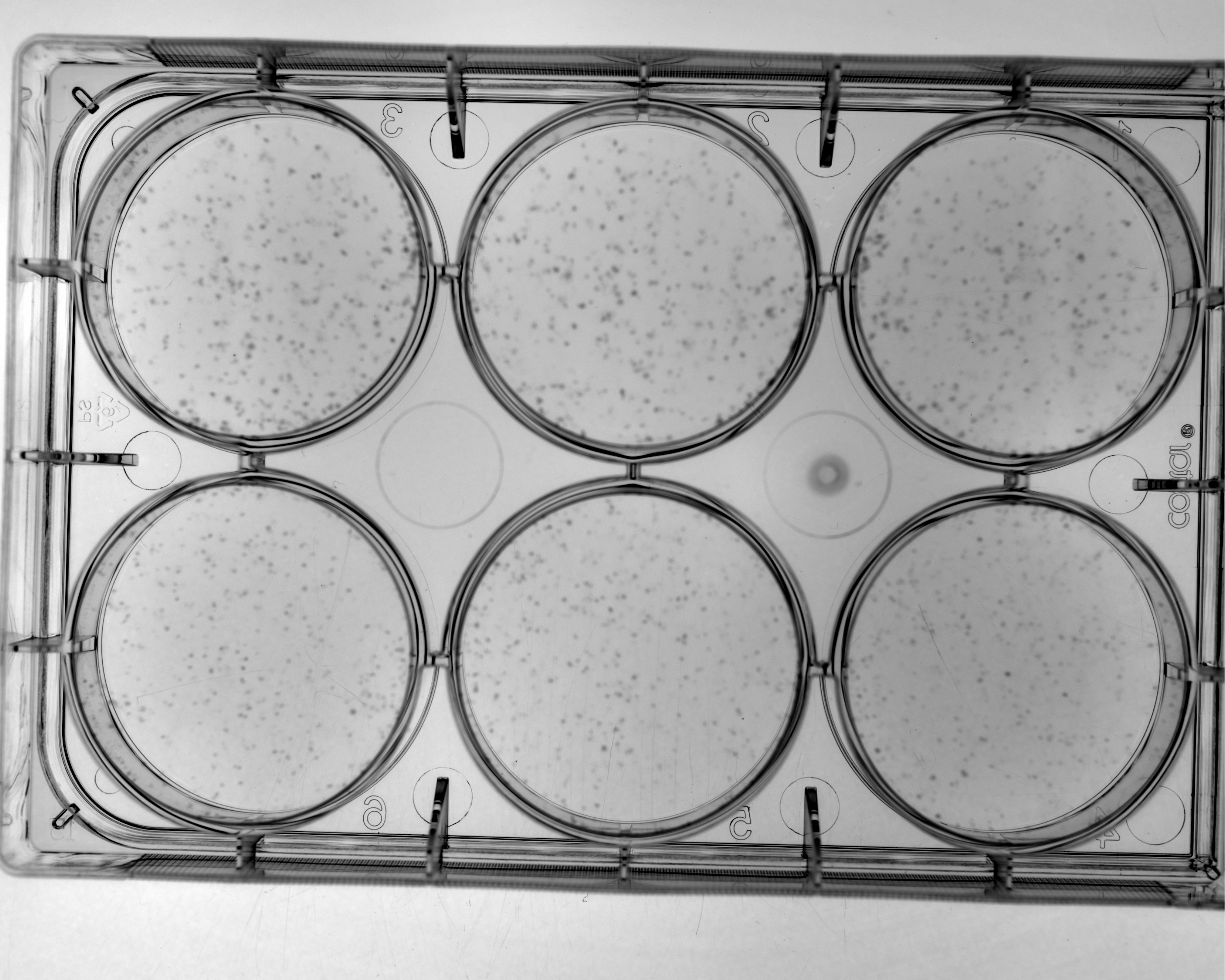

Supplement: Supplementary file 10 — Source data Fig. 3 [file 44318_2026_742_MOESM10_ESM.zip › FIgure 3/3E/A549/A549_3.tif]

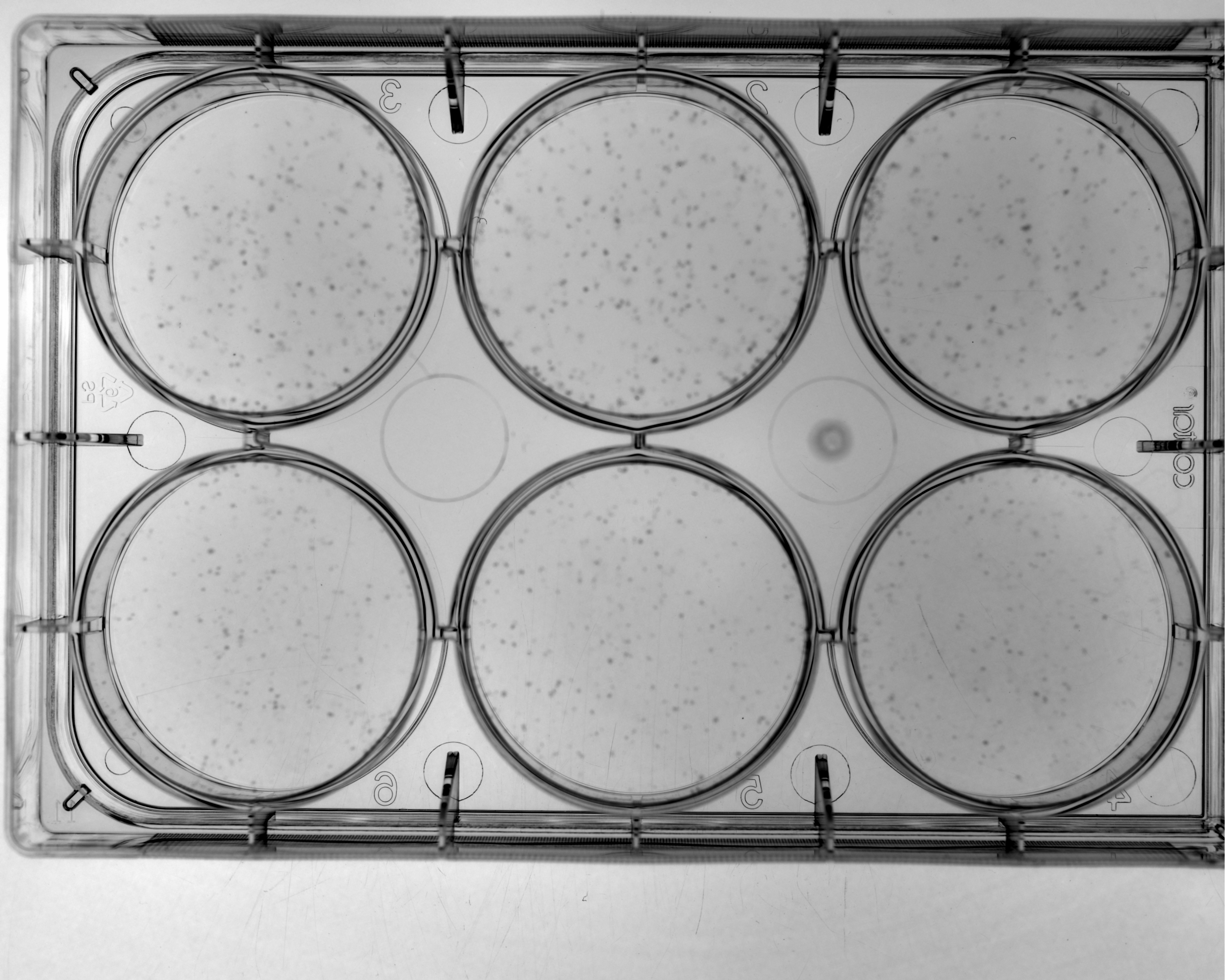

Supplement: Supplementary file 10 — Source data Fig. 3 [file 44318_2026_742_MOESM10_ESM.zip › FIgure 3/3E/A549/A549_2.tif]

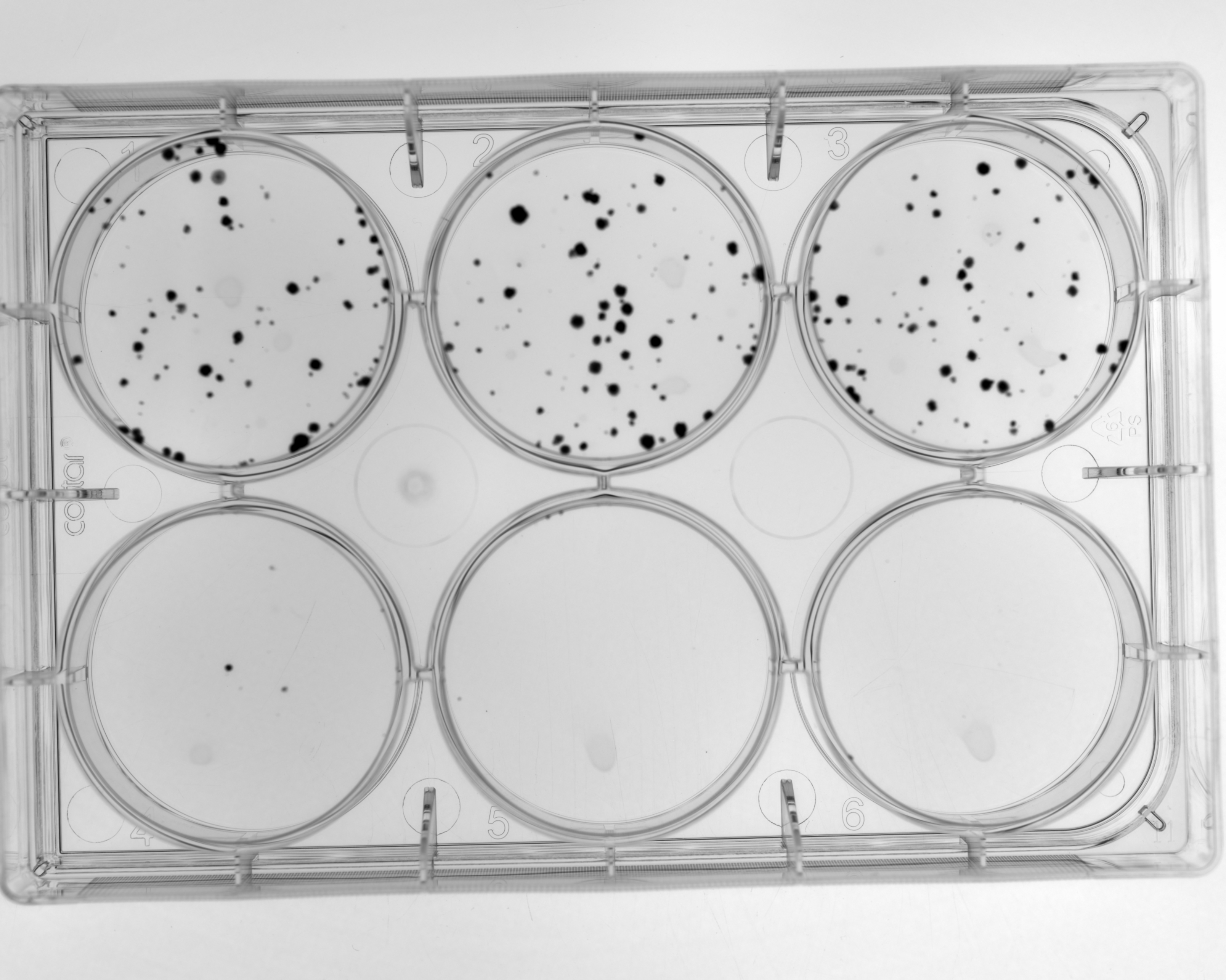

Supplement: Supplementary file 10 — Source data Fig. 3 [file 44318_2026_742_MOESM10_ESM.zip › FIgure 3/3E/DLD-1/DLD-1_1.tif]

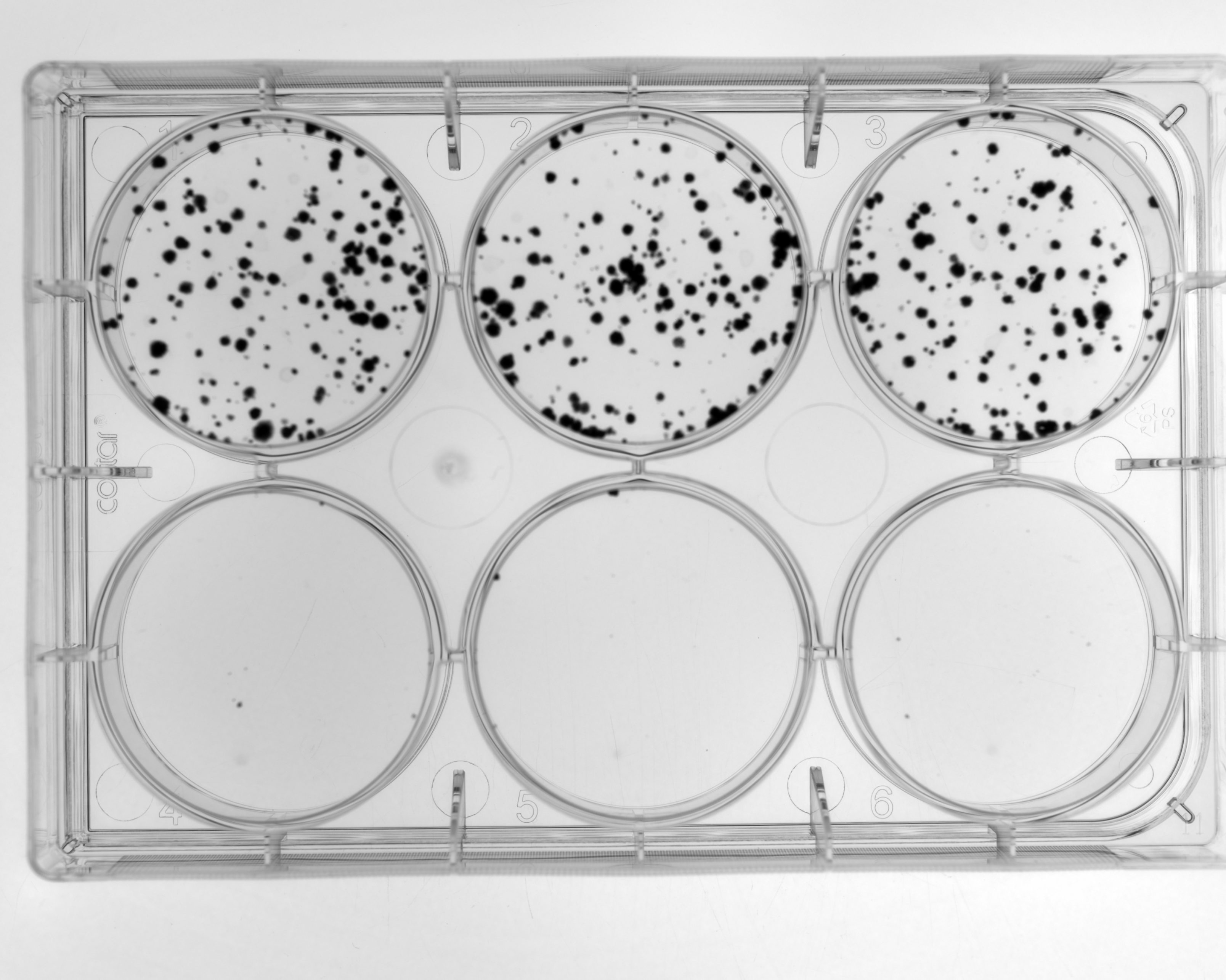

Supplement: Supplementary file 10 — Source data Fig. 3 [file 44318_2026_742_MOESM10_ESM.zip › FIgure 3/3E/DLD-1/DLD-1_3.tif]

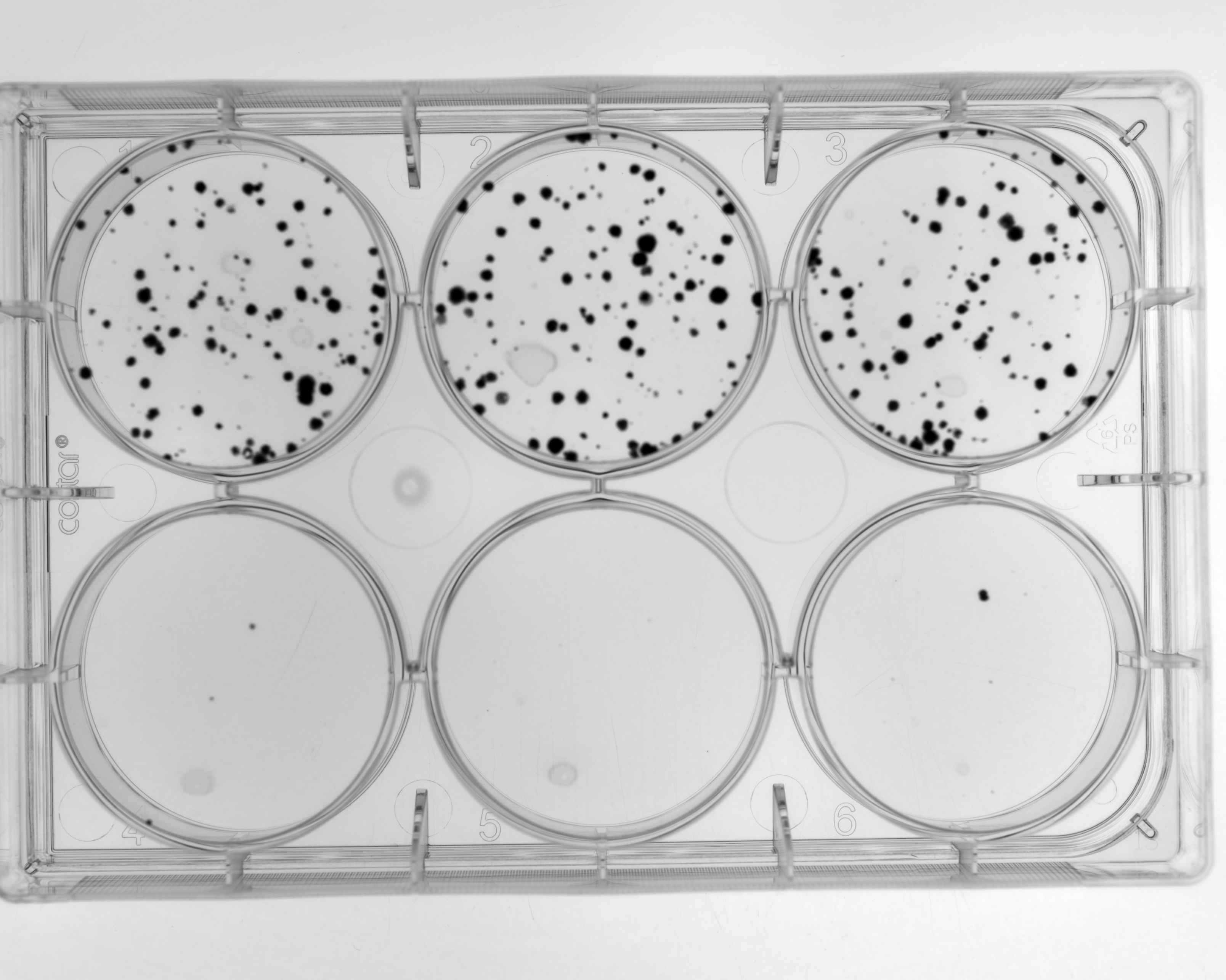

Supplement: Supplementary file 10 — Source data Fig. 3 [file 44318_2026_742_MOESM10_ESM.zip › FIgure 3/3E/DLD-1/DLD-1_2.tif]

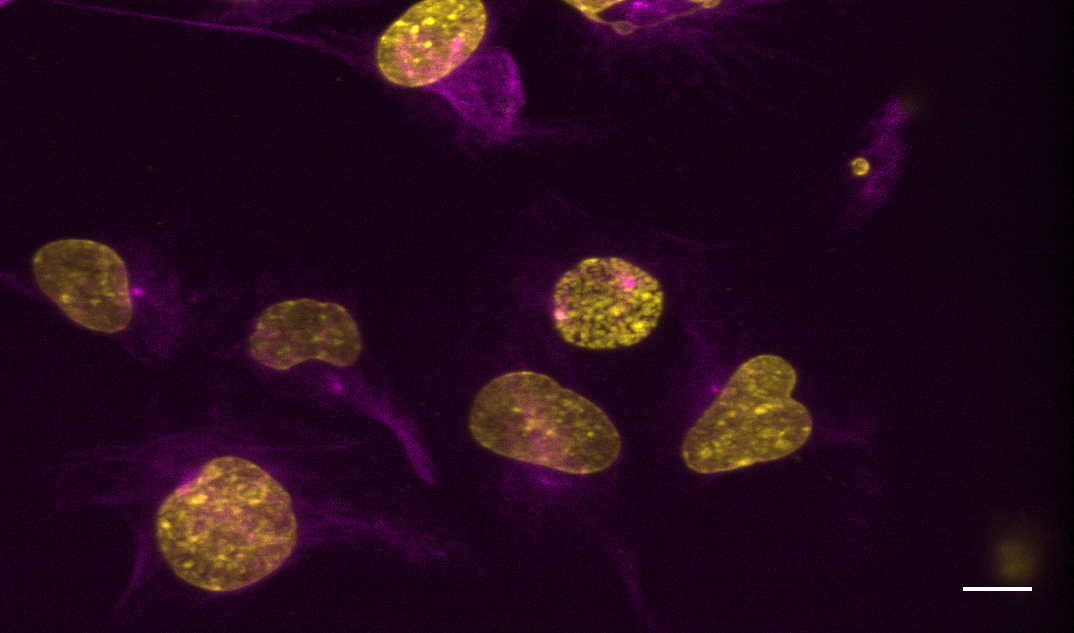

Supplement: Supplementary file 14 — Source data Fig. 7 [file 44318_2026_742_MOESM14_ESM.zip › FIgure 7/7C/HMEC_tr_10.png]

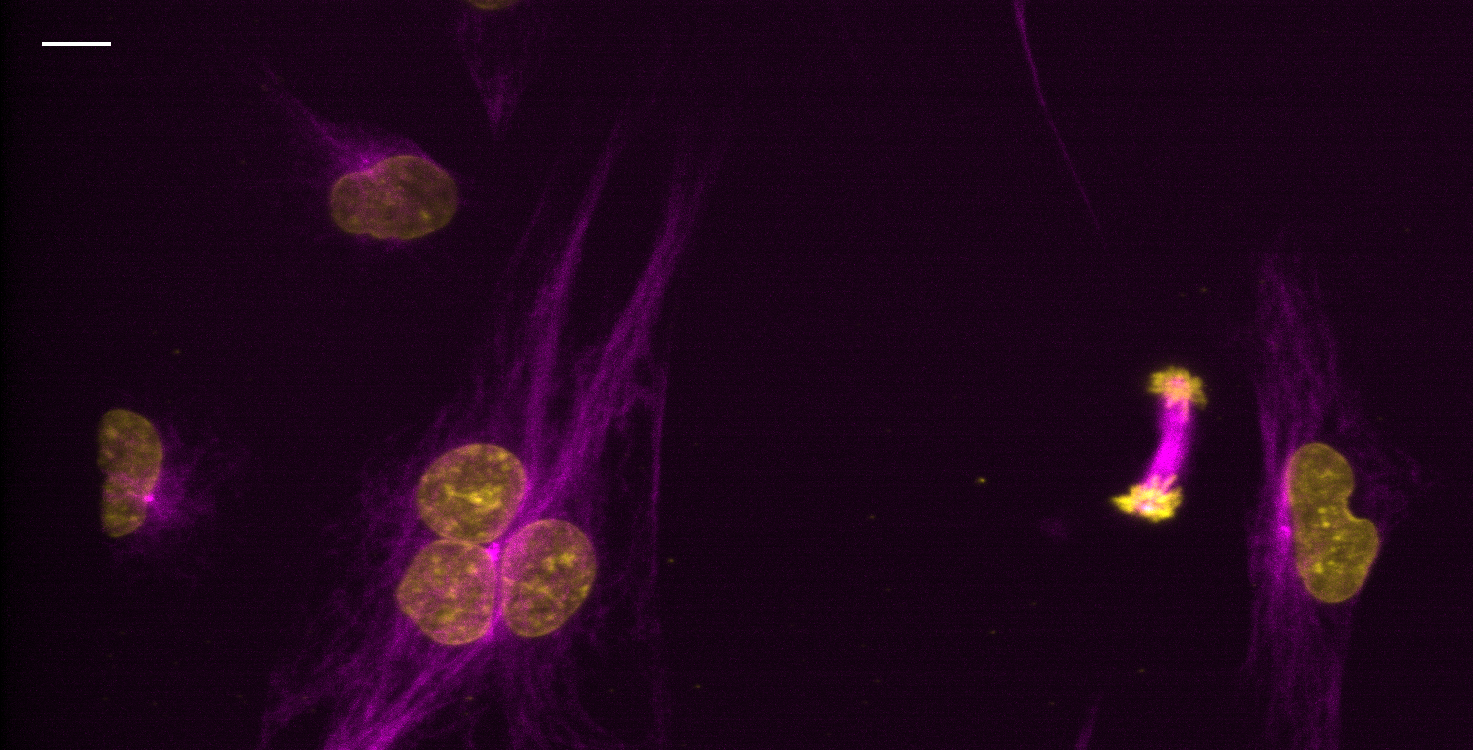

Supplement: Supplementary file 14 — Source data Fig. 7 [file 44318_2026_742_MOESM14_ESM.zip › FIgure 7/7C/HMEC_untr_40.png]

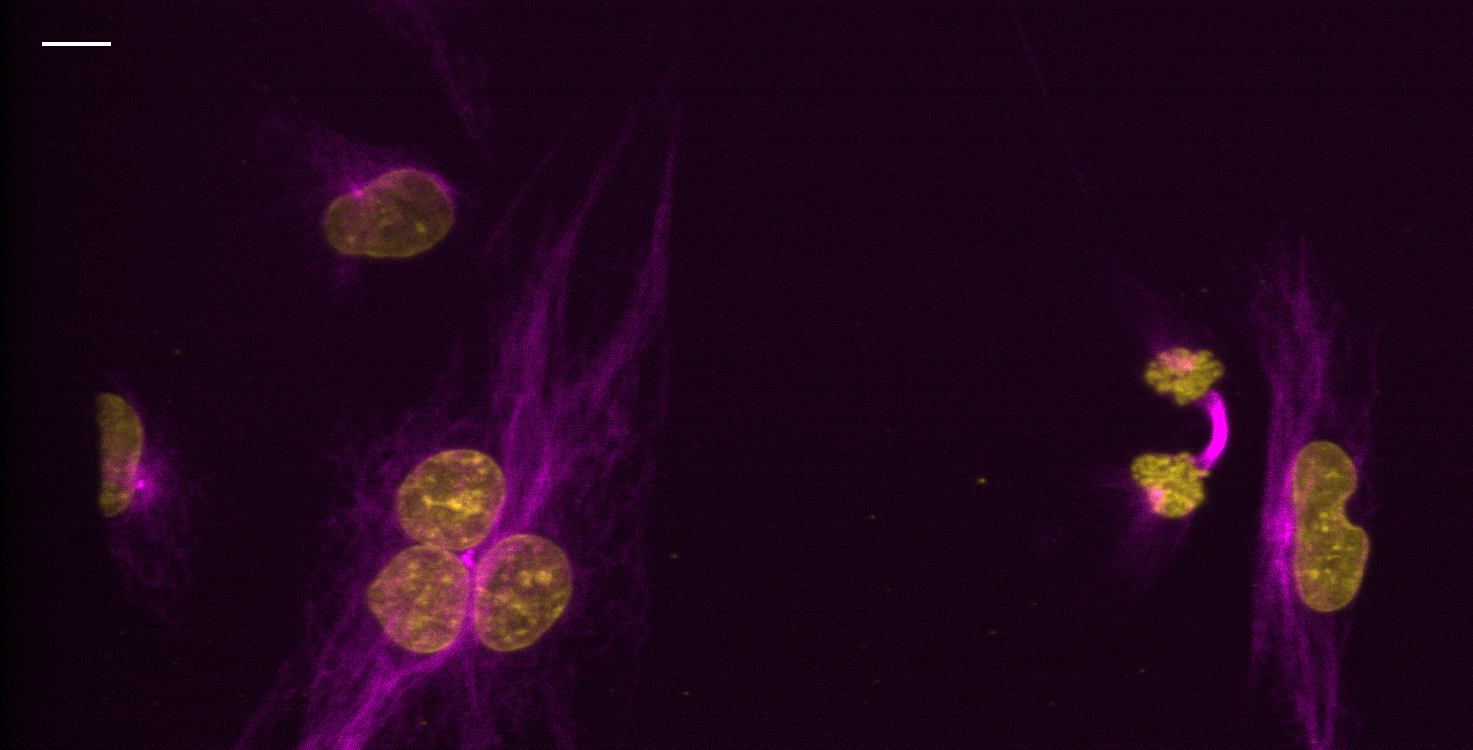

Supplement: Supplementary file 14 — Source data Fig. 7 [file 44318_2026_742_MOESM14_ESM.zip › FIgure 7/7C/HMEC_untr_50.png]

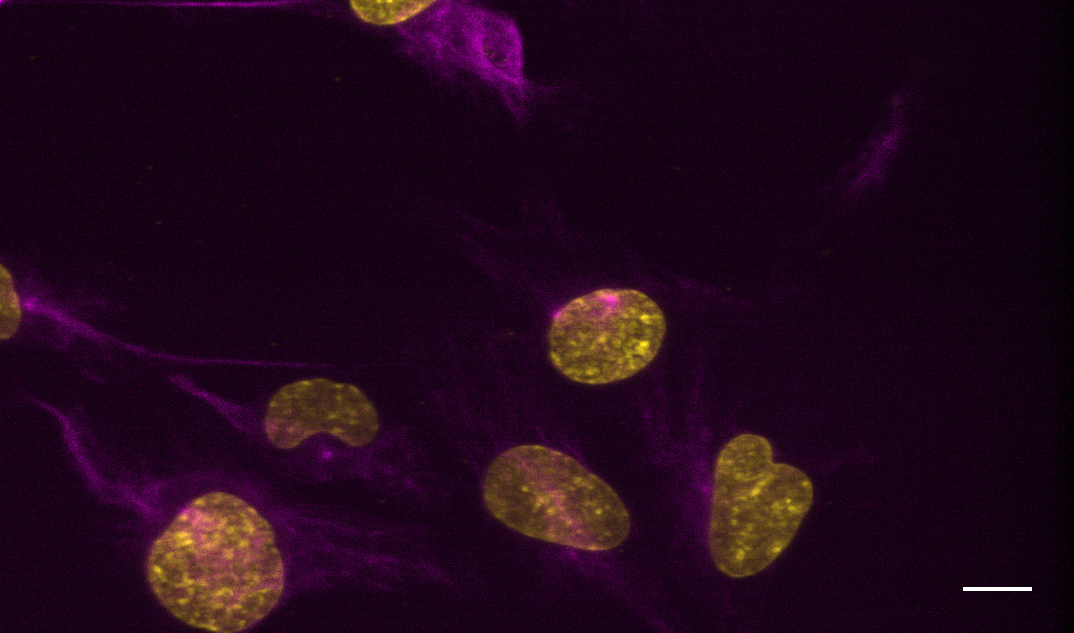

Supplement: Supplementary file 14 — Source data Fig. 7 [file 44318_2026_742_MOESM14_ESM.zip › FIgure 7/7C/HMEC_tr_0.png]

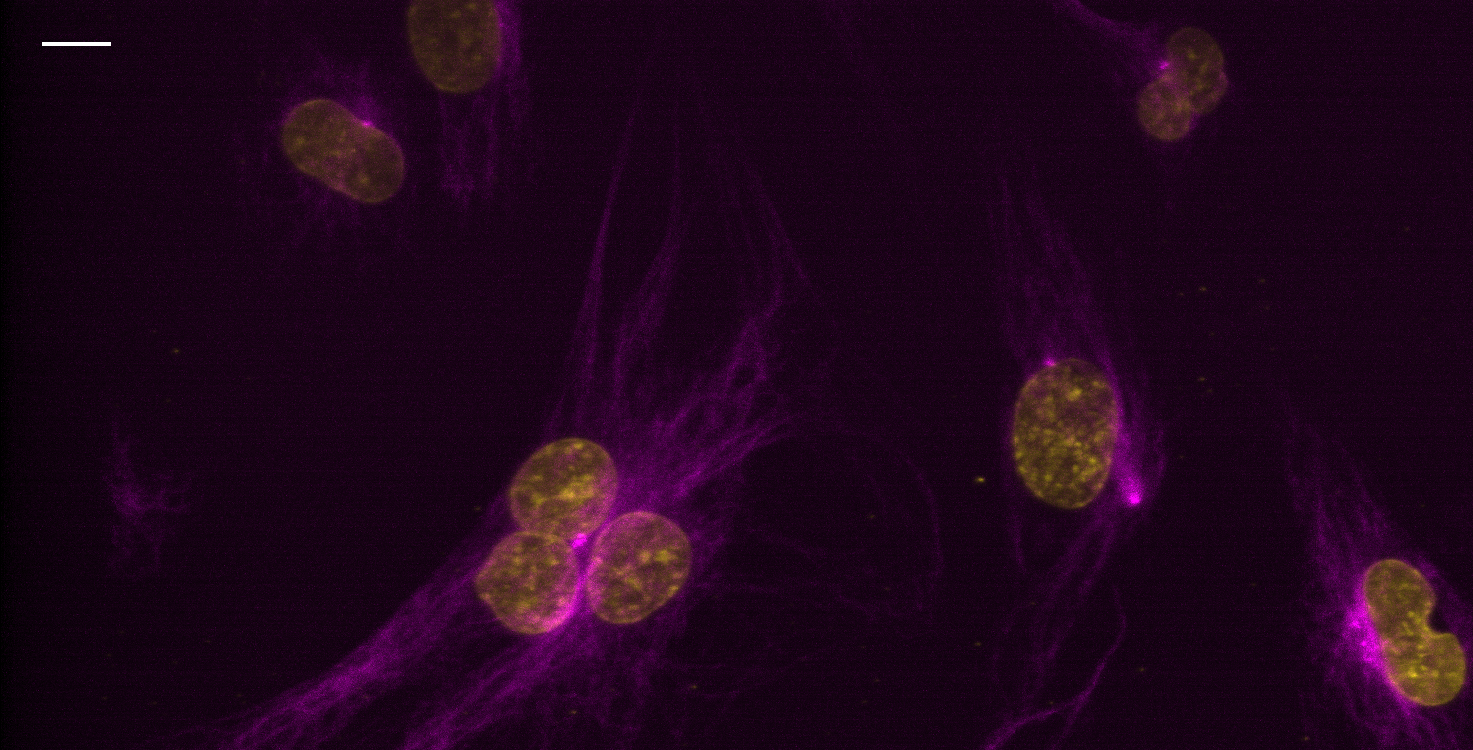

Supplement: Supplementary file 14 — Source data Fig. 7 [file 44318_2026_742_MOESM14_ESM.zip › FIgure 7/7C/HMEC_untr_0.png]

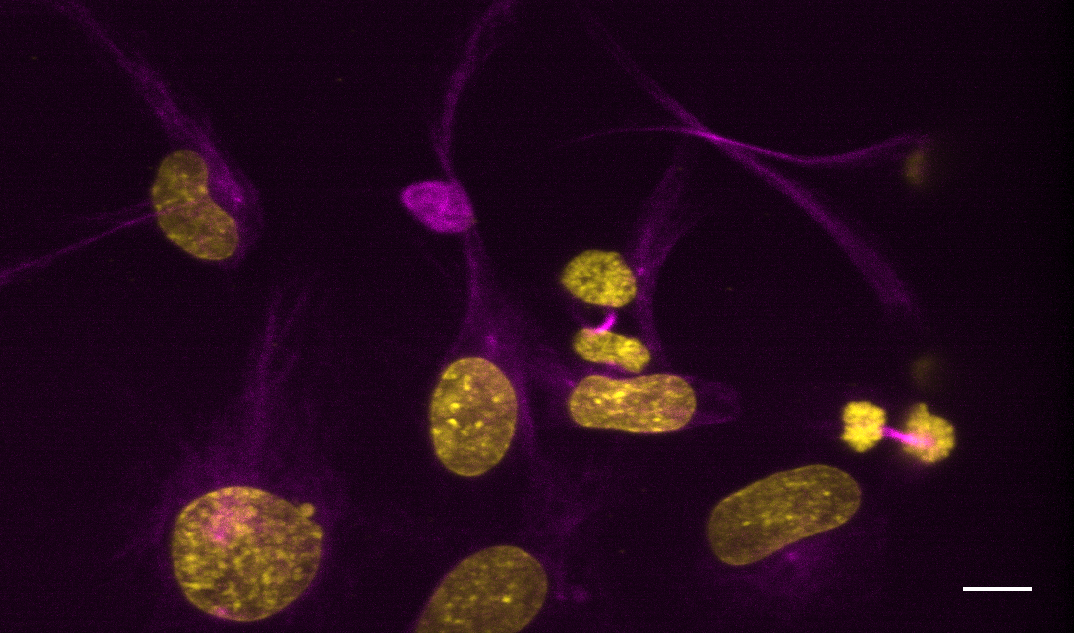

Supplement: Supplementary file 14 — Source data Fig. 7 [file 44318_2026_742_MOESM14_ESM.zip › FIgure 7/7C/HMEC_tr_60.png]

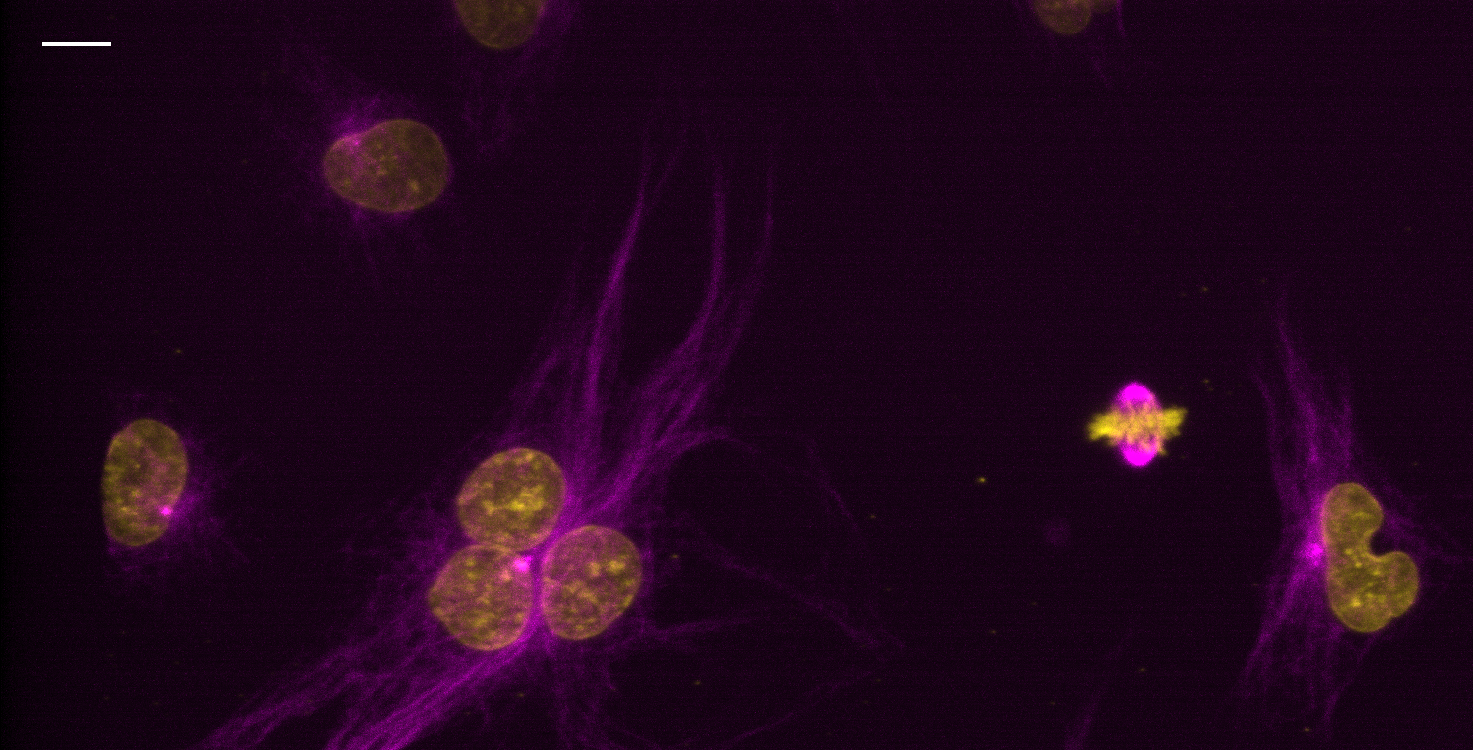

Supplement: Supplementary file 14 — Source data Fig. 7 [file 44318_2026_742_MOESM14_ESM.zip › FIgure 7/7C/HMEC_untr_30.png]

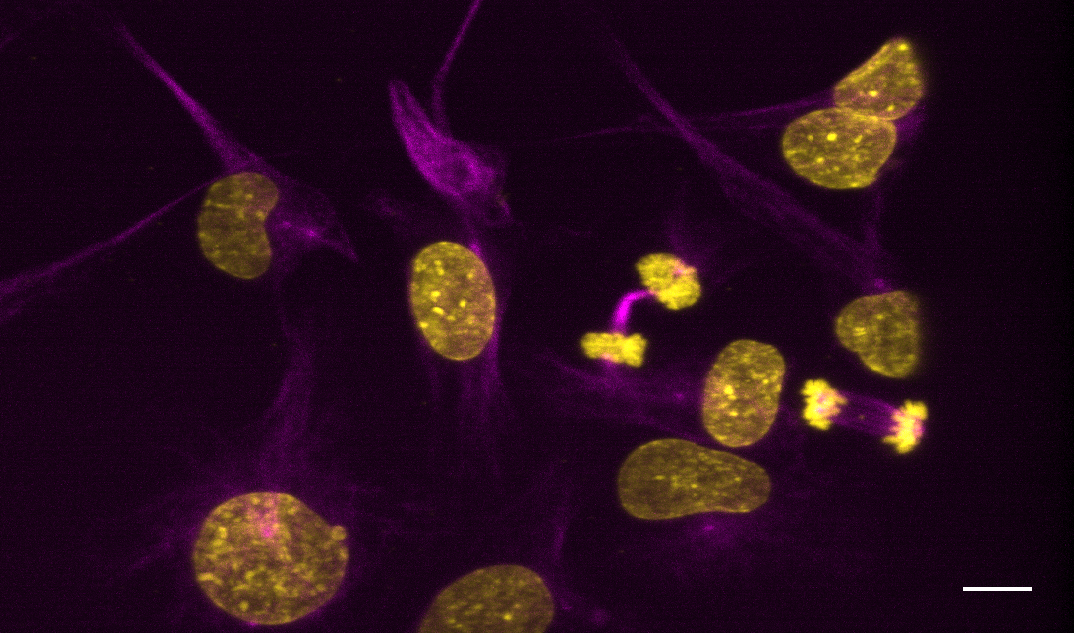

Supplement: Supplementary file 14 — Source data Fig. 7 [file 44318_2026_742_MOESM14_ESM.zip › FIgure 7/7C/HMEC_tr_50.png]

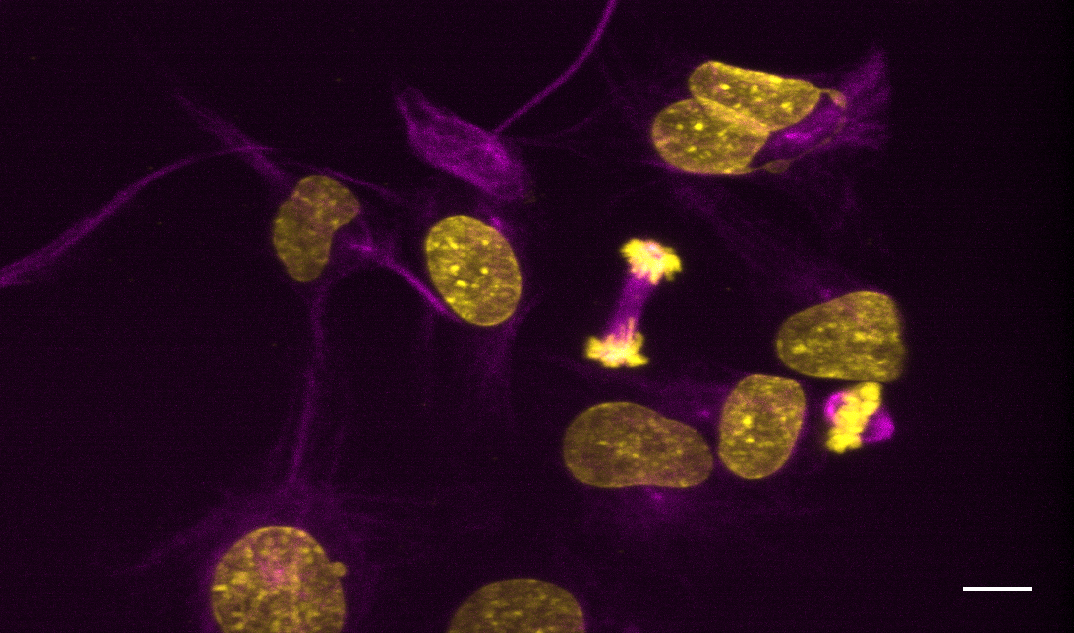

Supplement: Supplementary file 14 — Source data Fig. 7 [file 44318_2026_742_MOESM14_ESM.zip › FIgure 7/7C/HMEC_tr_40.png]

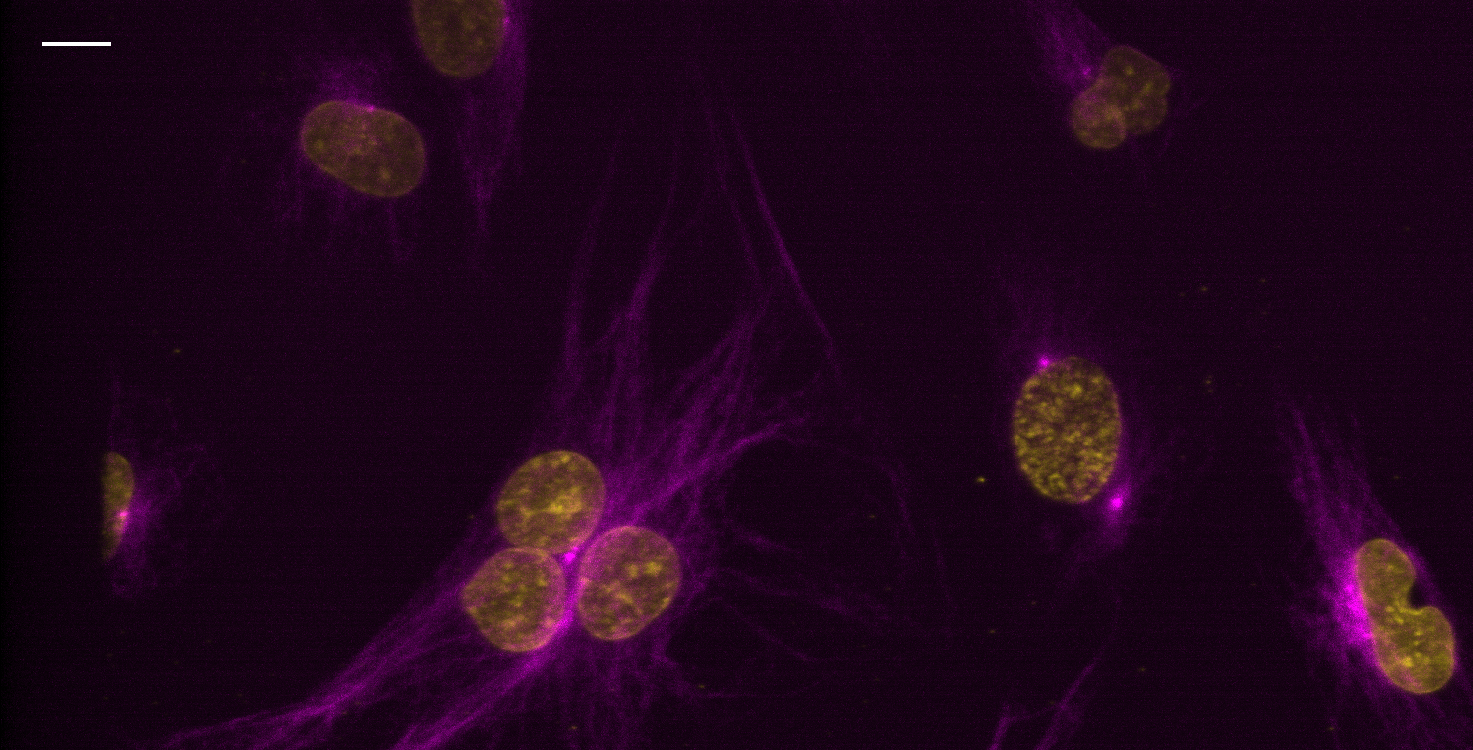

Supplement: Supplementary file 14 — Source data Fig. 7 [file 44318_2026_742_MOESM14_ESM.zip › FIgure 7/7C/HMEC_untr_10.png]

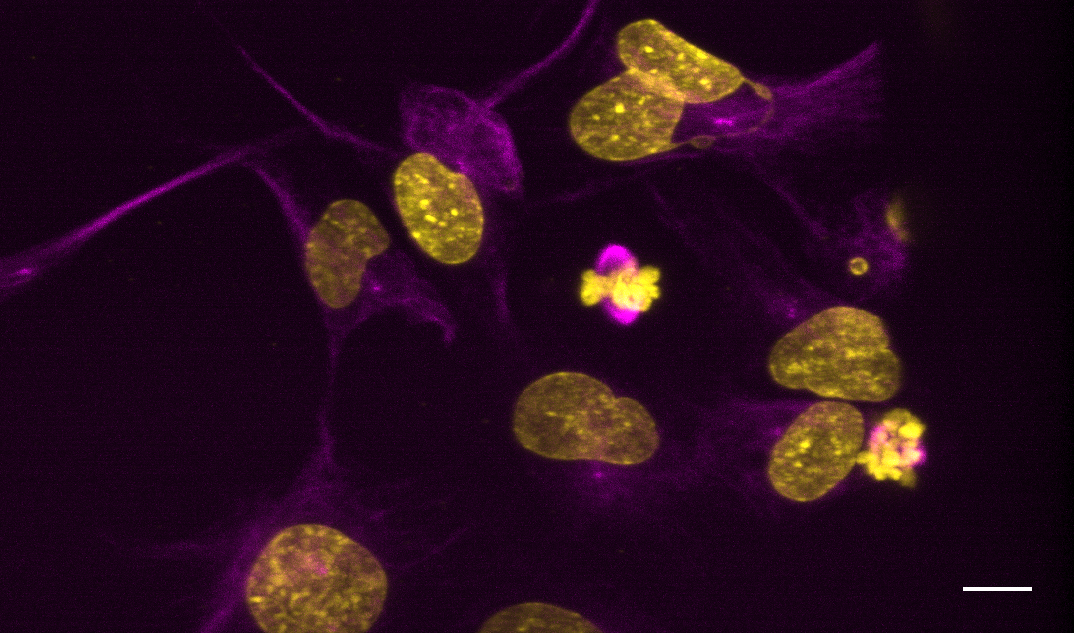

Supplement: Supplementary file 14 — Source data Fig. 7 [file 44318_2026_742_MOESM14_ESM.zip › FIgure 7/7C/HMEC_tr_30.png]

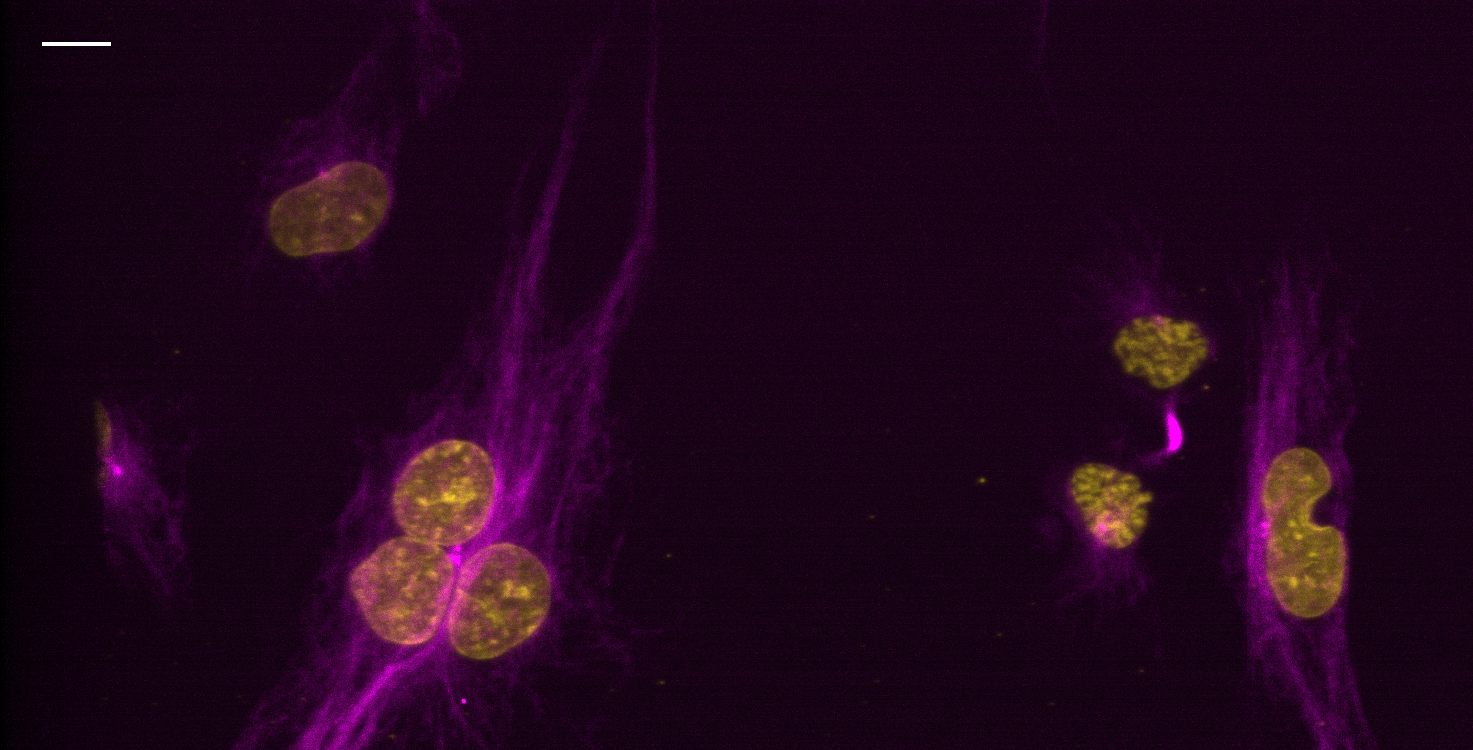

Supplement: Supplementary file 14 — Source data Fig. 7 [file 44318_2026_742_MOESM14_ESM.zip › FIgure 7/7C/HMEC_untr_60.png]

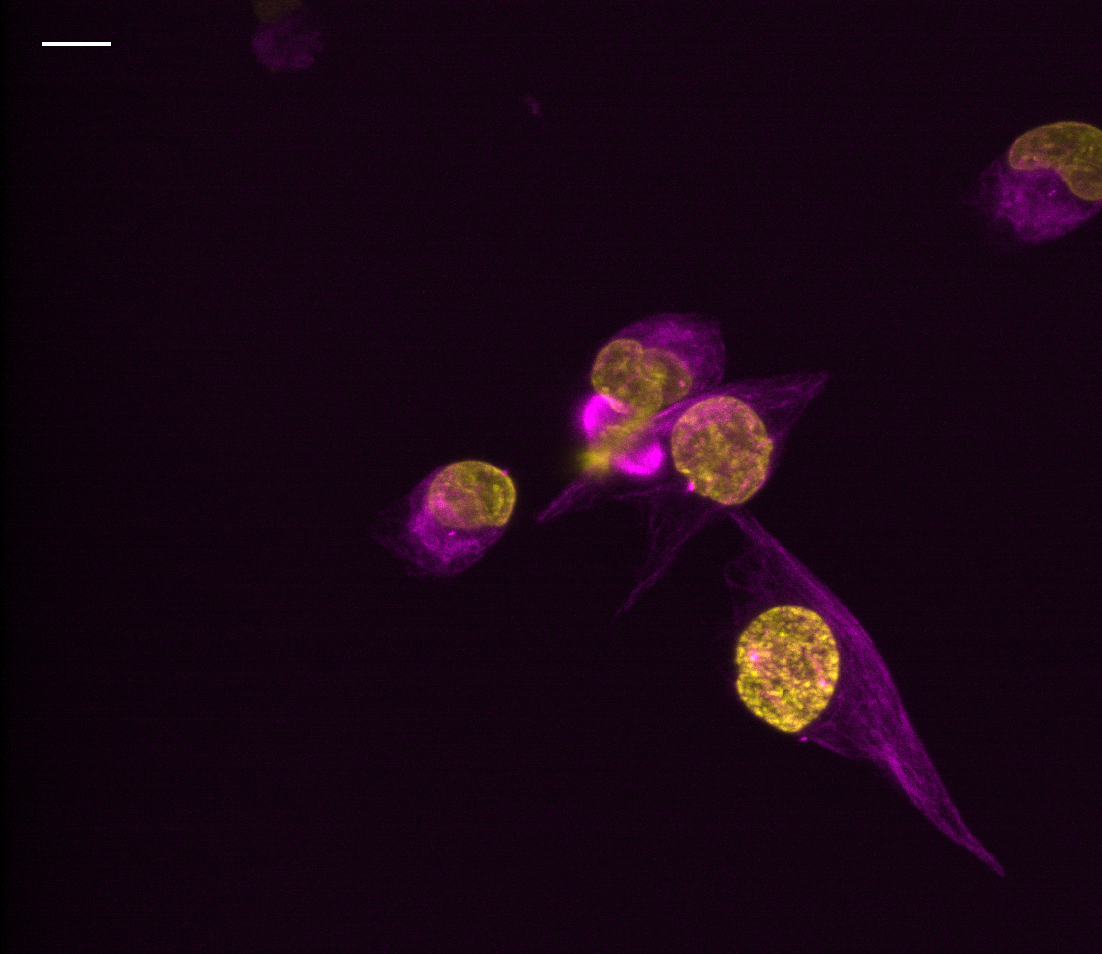

Supplement: Supplementary file 14 — Source data Fig. 7 [file 44318_2026_742_MOESM14_ESM.zip › FIgure 7/7B/Mia_untr_10.png]

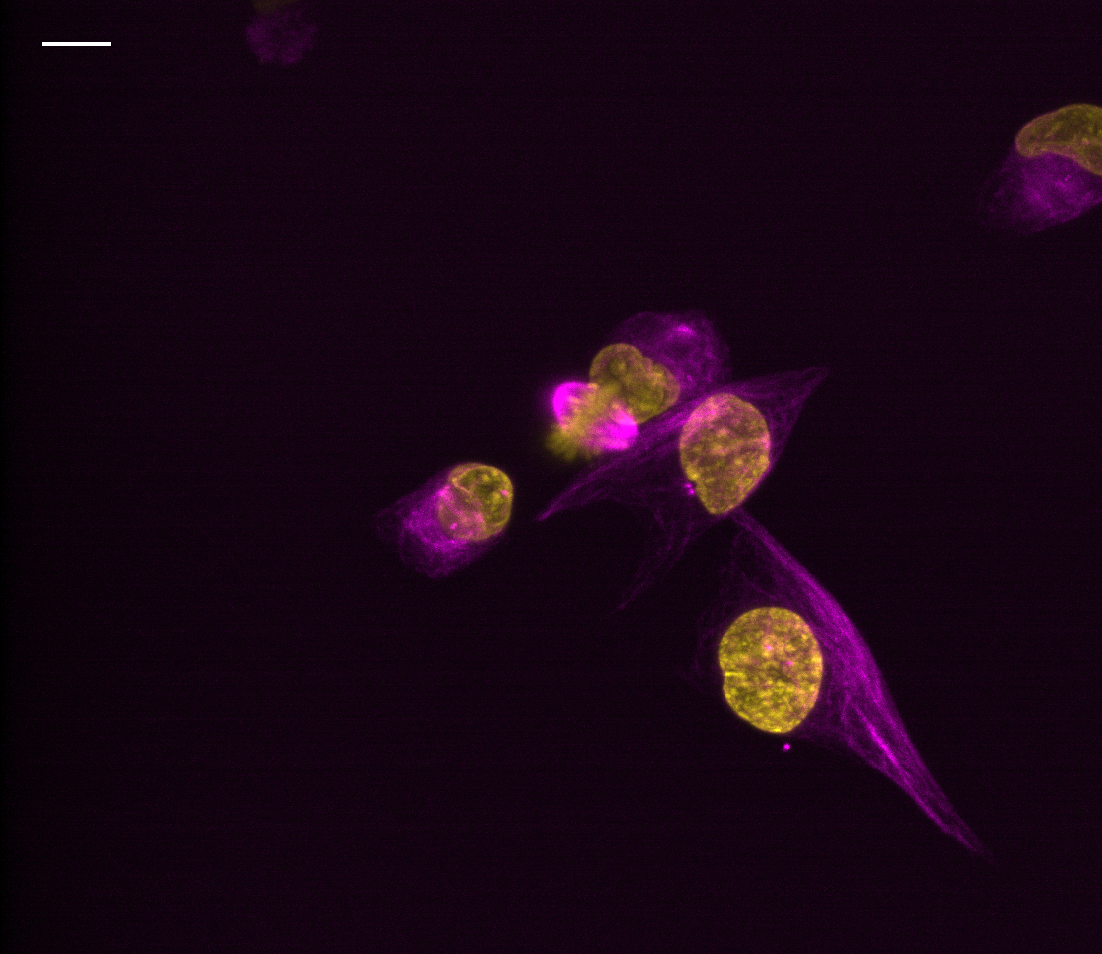

Supplement: Supplementary file 14 — Source data Fig. 7 [file 44318_2026_742_MOESM14_ESM.zip › FIgure 7/7B/Mia_untr_0.png]

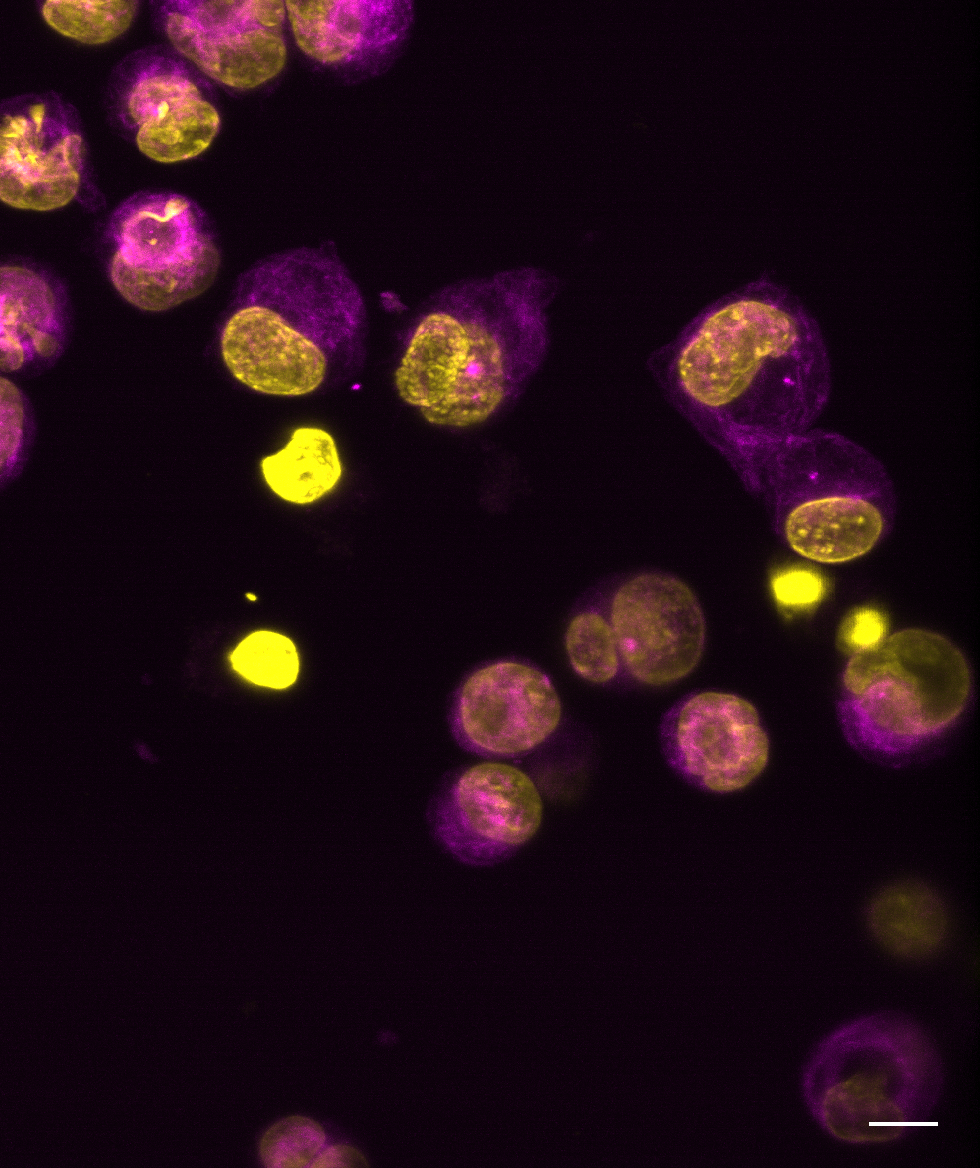

Supplement: Supplementary file 14 — Source data Fig. 7 [file 44318_2026_742_MOESM14_ESM.zip › FIgure 7/7B/Mia_tr_10.png]

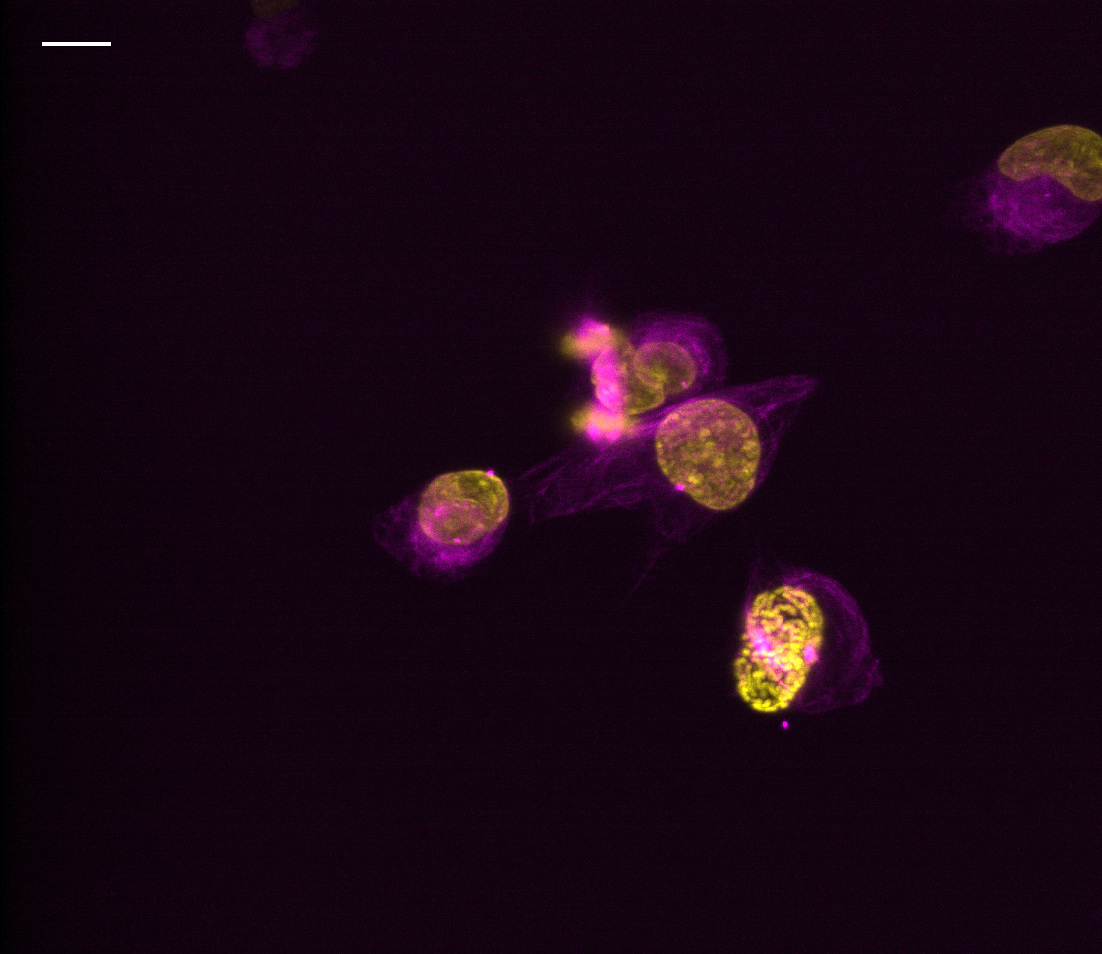

Supplement: Supplementary file 14 — Source data Fig. 7 [file 44318_2026_742_MOESM14_ESM.zip › FIgure 7/7B/Mia_untr_20.png]

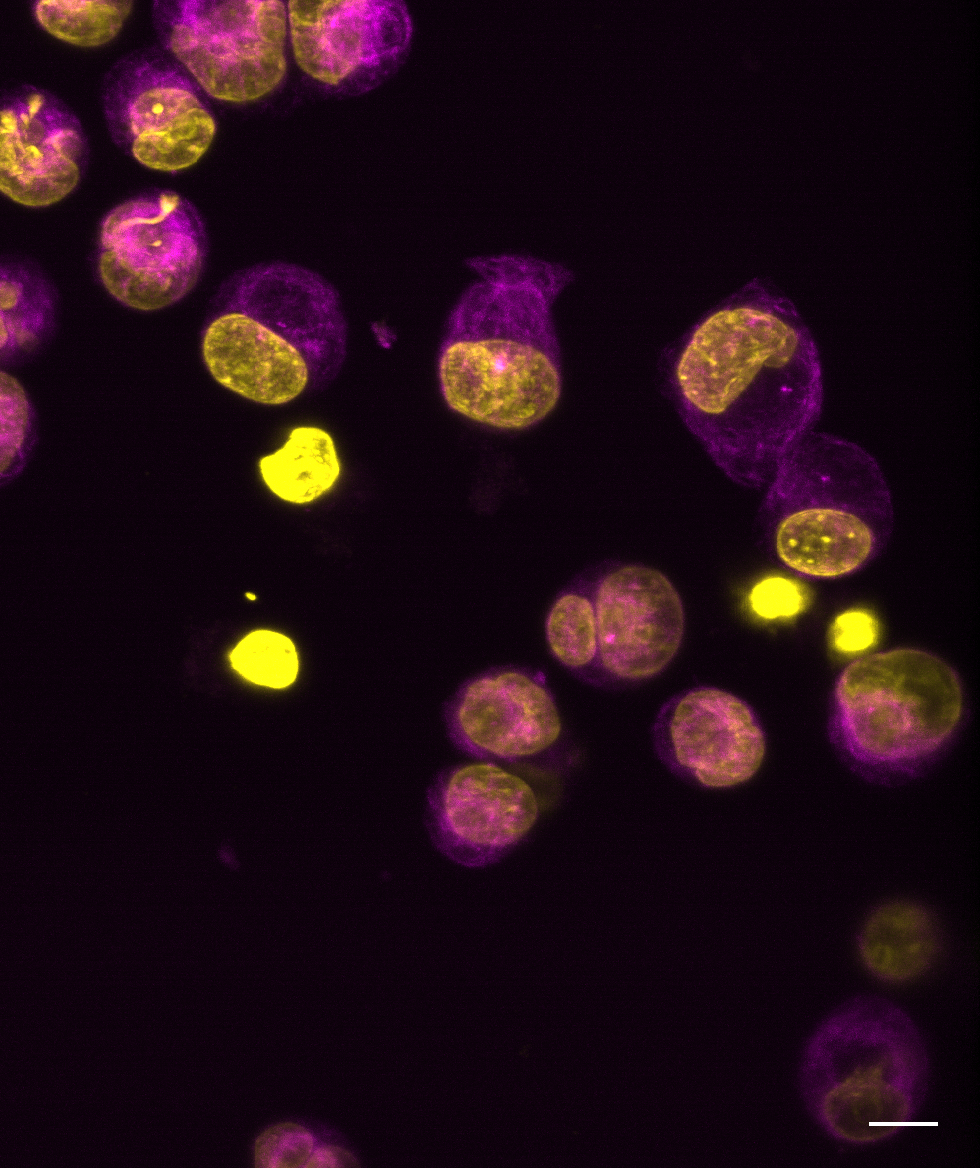

Supplement: Supplementary file 14 — Source data Fig. 7 [file 44318_2026_742_MOESM14_ESM.zip › FIgure 7/7B/Mia_tr_0.png]

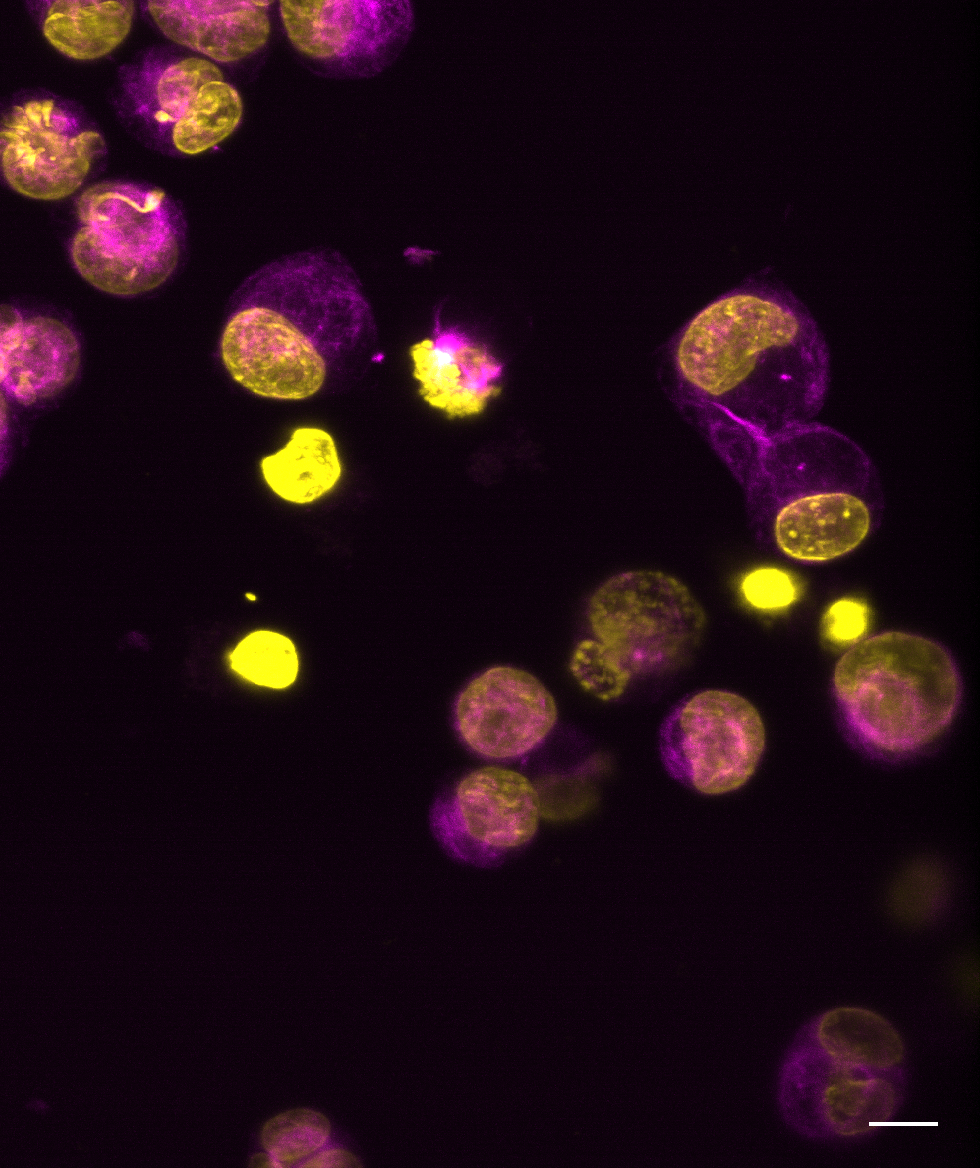

Supplement: Supplementary file 14 — Source data Fig. 7 [file 44318_2026_742_MOESM14_ESM.zip › FIgure 7/7B/Mia_tr_20.png]

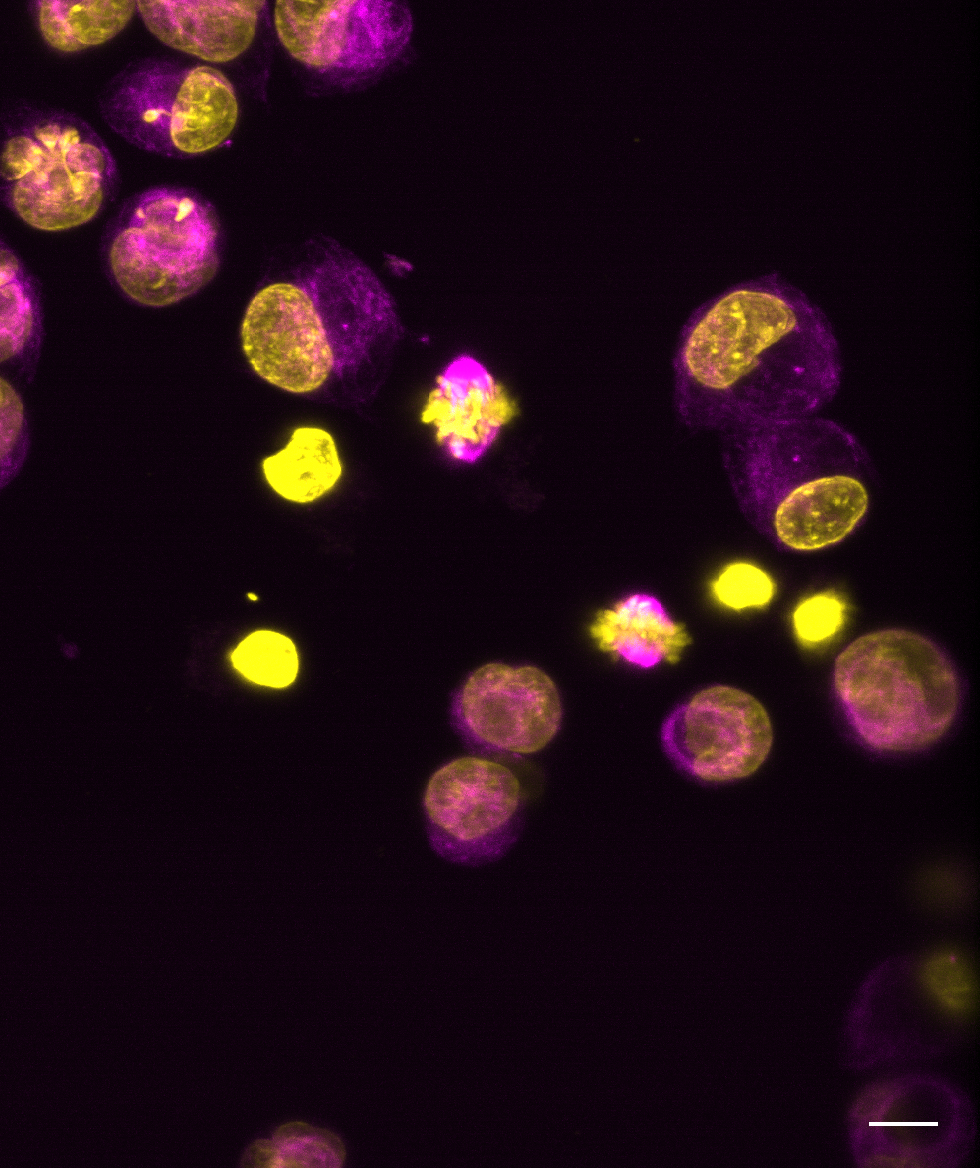

Supplement: Supplementary file 14 — Source data Fig. 7 [file 44318_2026_742_MOESM14_ESM.zip › FIgure 7/7B/Mia_tr_50.png]

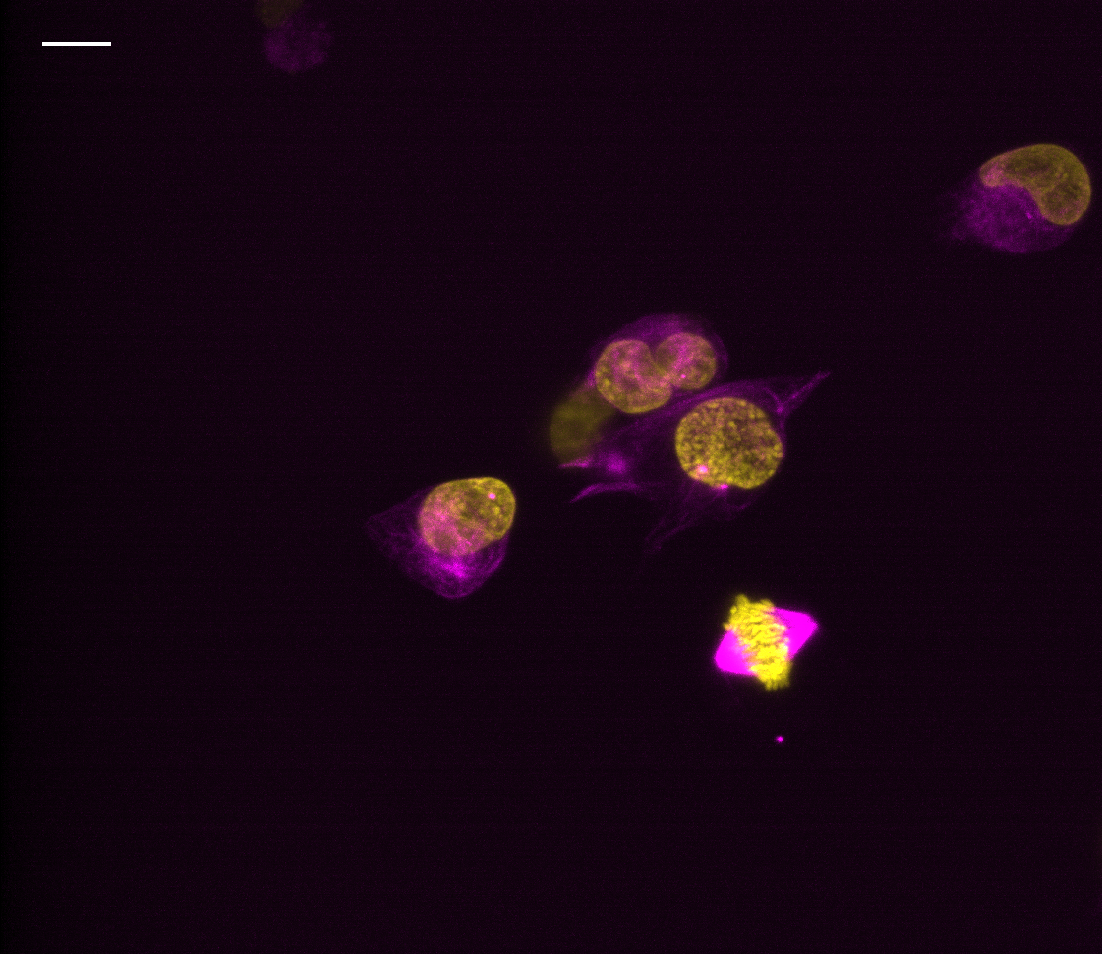

Supplement: Supplementary file 14 — Source data Fig. 7 [file 44318_2026_742_MOESM14_ESM.zip › FIgure 7/7B/Mia_untr_50.png]

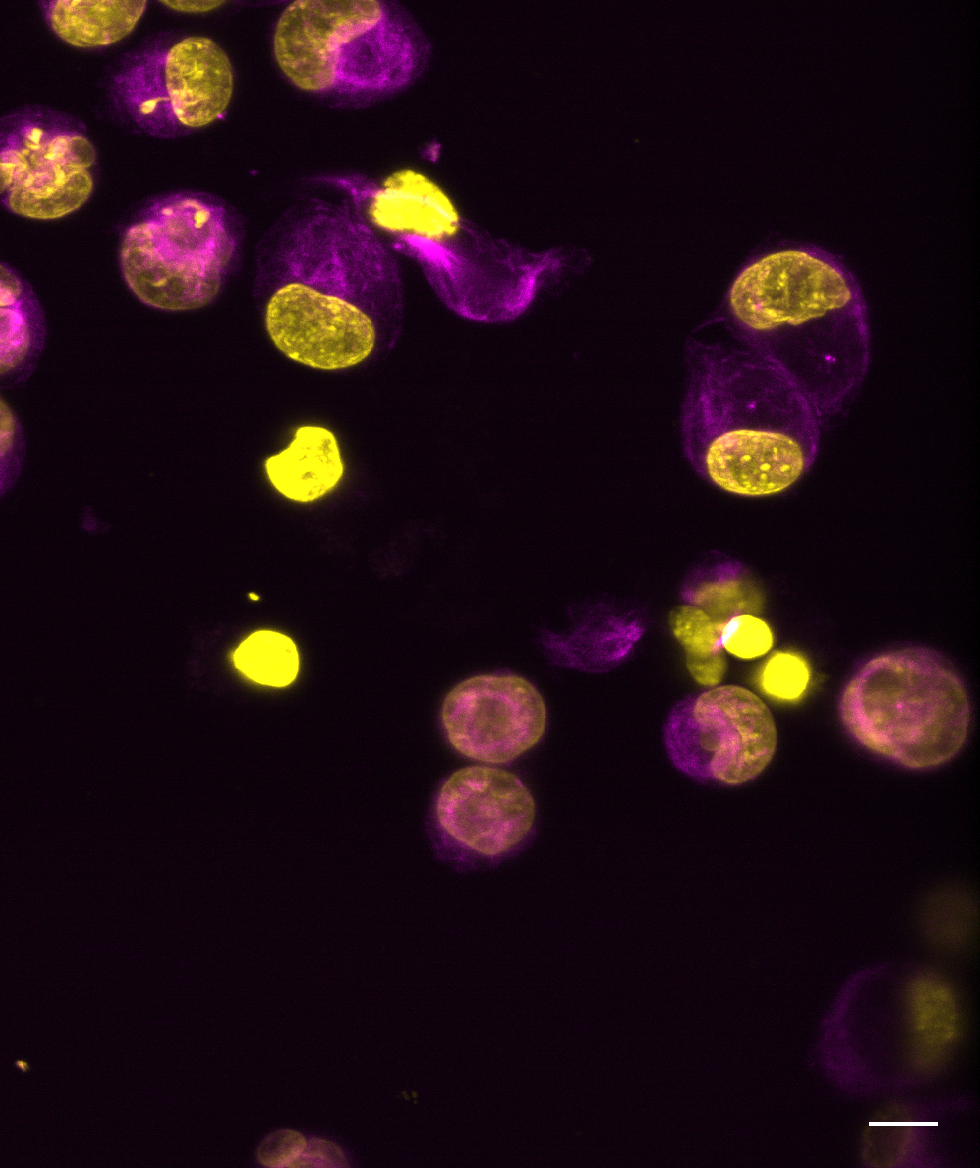

Supplement: Supplementary file 14 — Source data Fig. 7 [file 44318_2026_742_MOESM14_ESM.zip › FIgure 7/7B/Mia_tr_90.png]

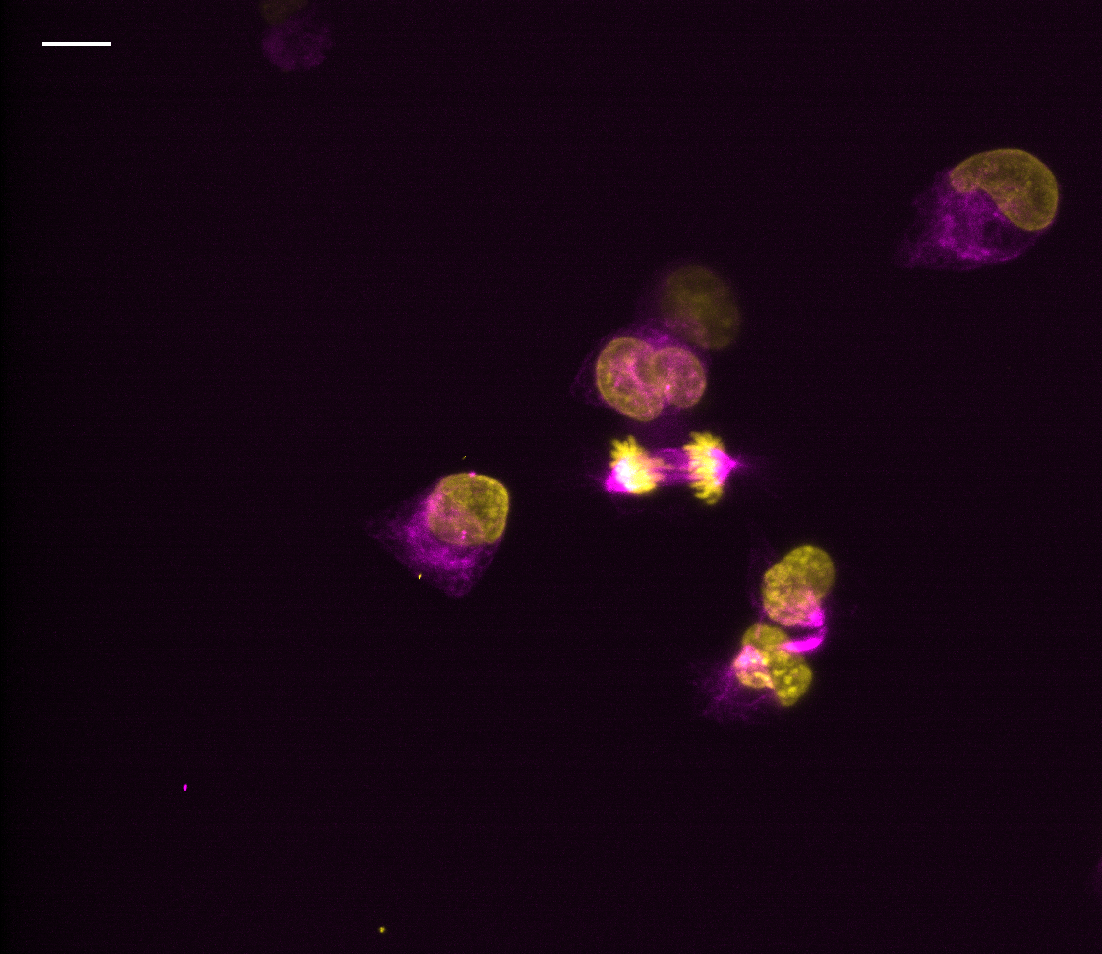

Supplement: Supplementary file 14 — Source data Fig. 7 [file 44318_2026_742_MOESM14_ESM.zip › FIgure 7/7B/Mia_untr_90.png]

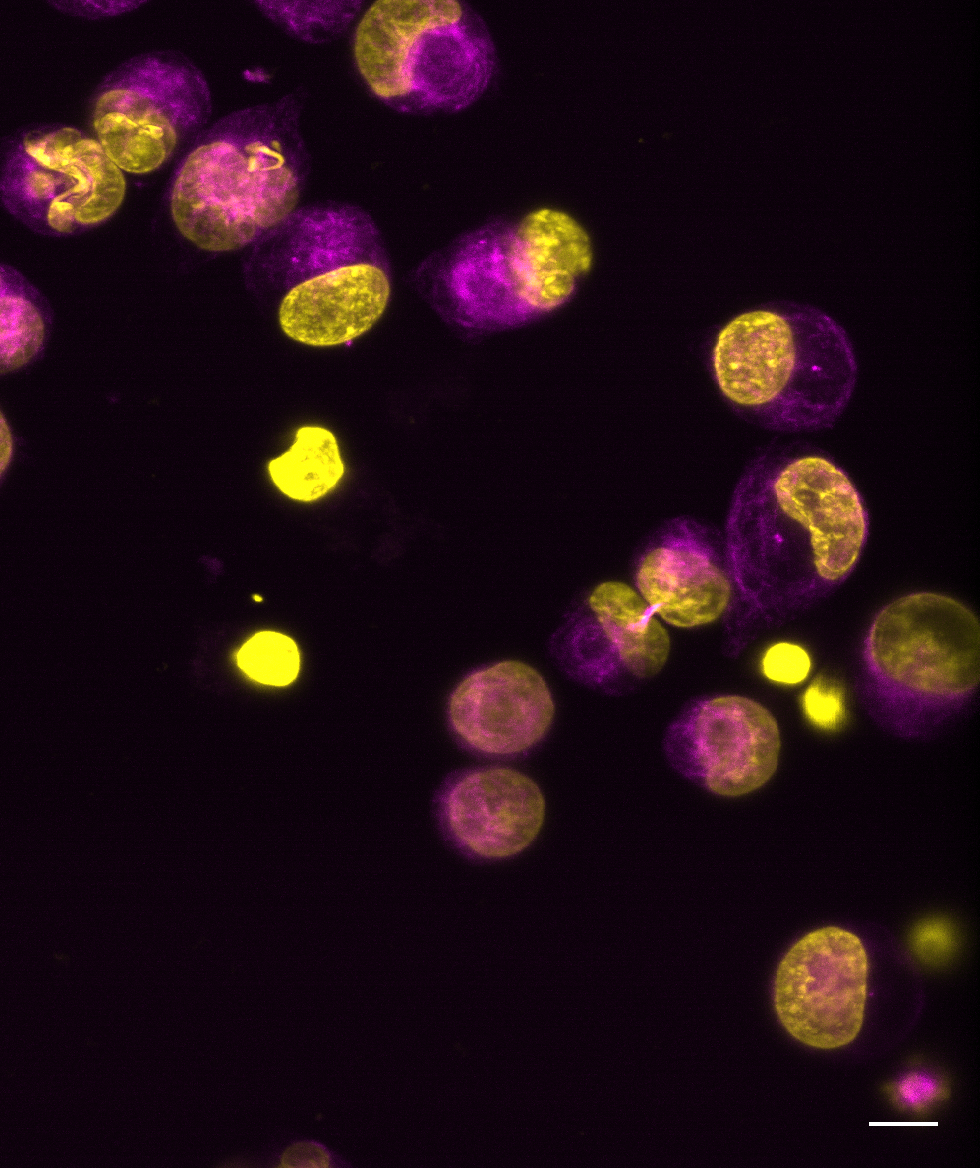

Supplement: Supplementary file 14 — Source data Fig. 7 [file 44318_2026_742_MOESM14_ESM.zip › FIgure 7/7B/Mia_tr_180.png]

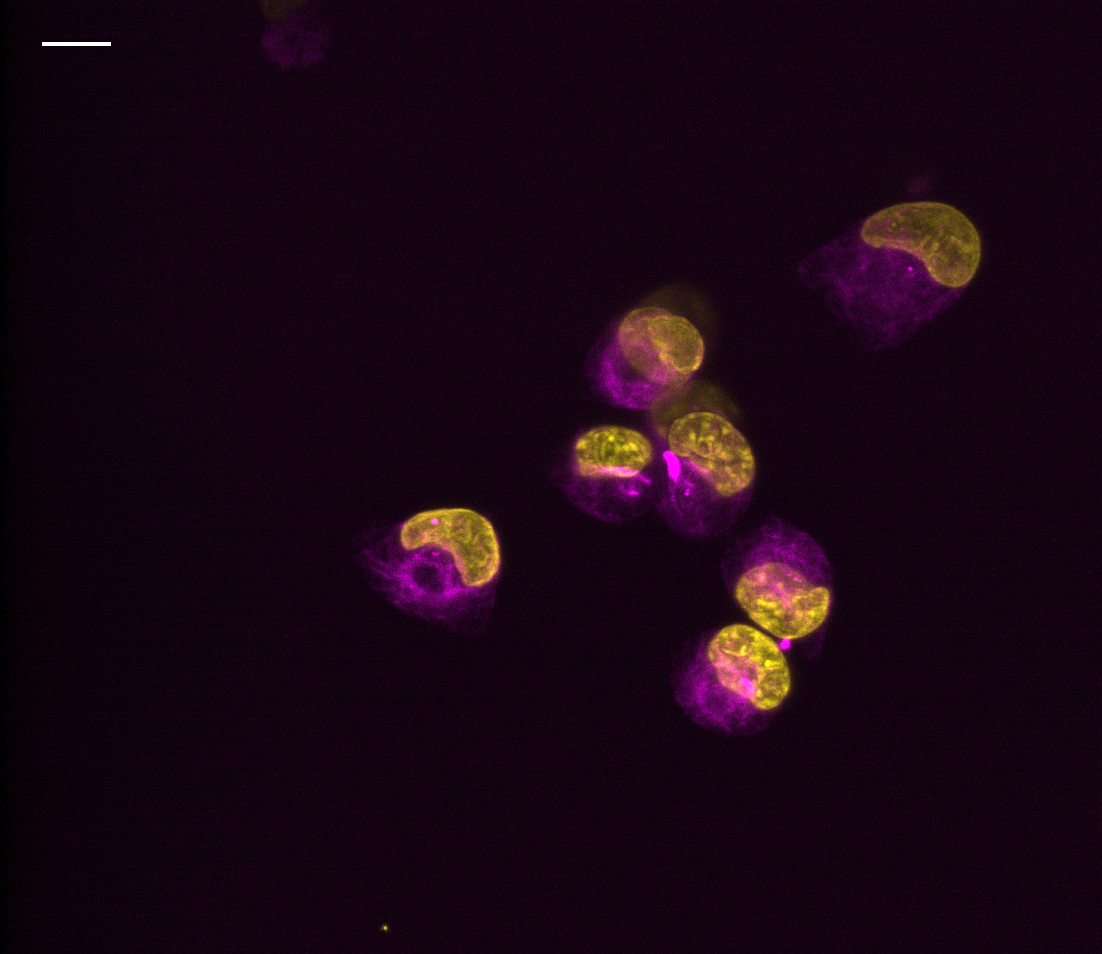

Supplement: Supplementary file 14 — Source data Fig. 7 [file 44318_2026_742_MOESM14_ESM.zip › FIgure 7/7B/Mia_untr_180.png]

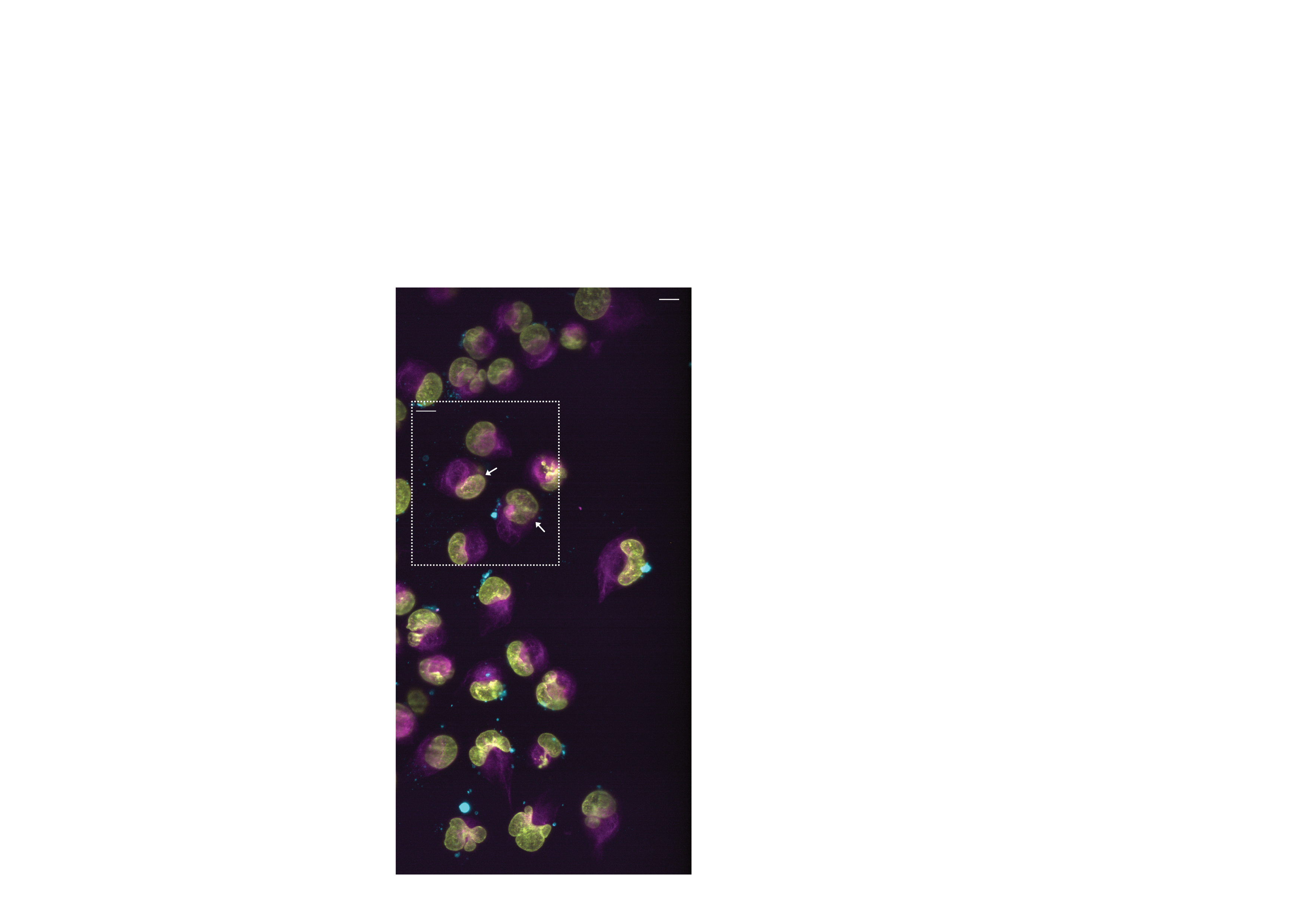

Supplement: Supplementary file 14 — Source data Fig. 7 [file 44318_2026_742_MOESM14_ESM.zip › FIgure 7/7E/Mia_pre.tiff]

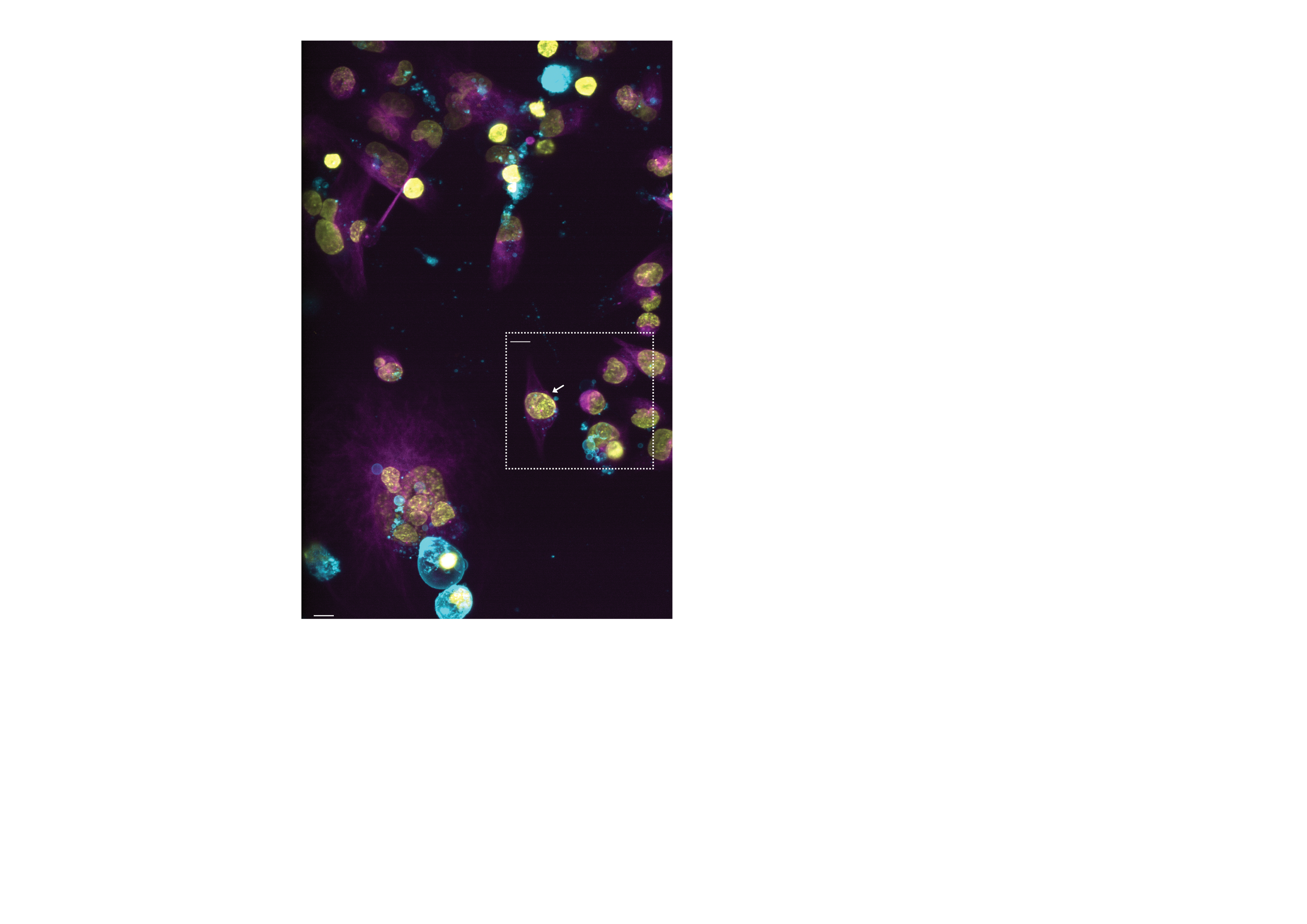

Supplement: Supplementary file 14 — Source data Fig. 7 [file 44318_2026_742_MOESM14_ESM.zip › FIgure 7/7E/MDA_pre.tiff]

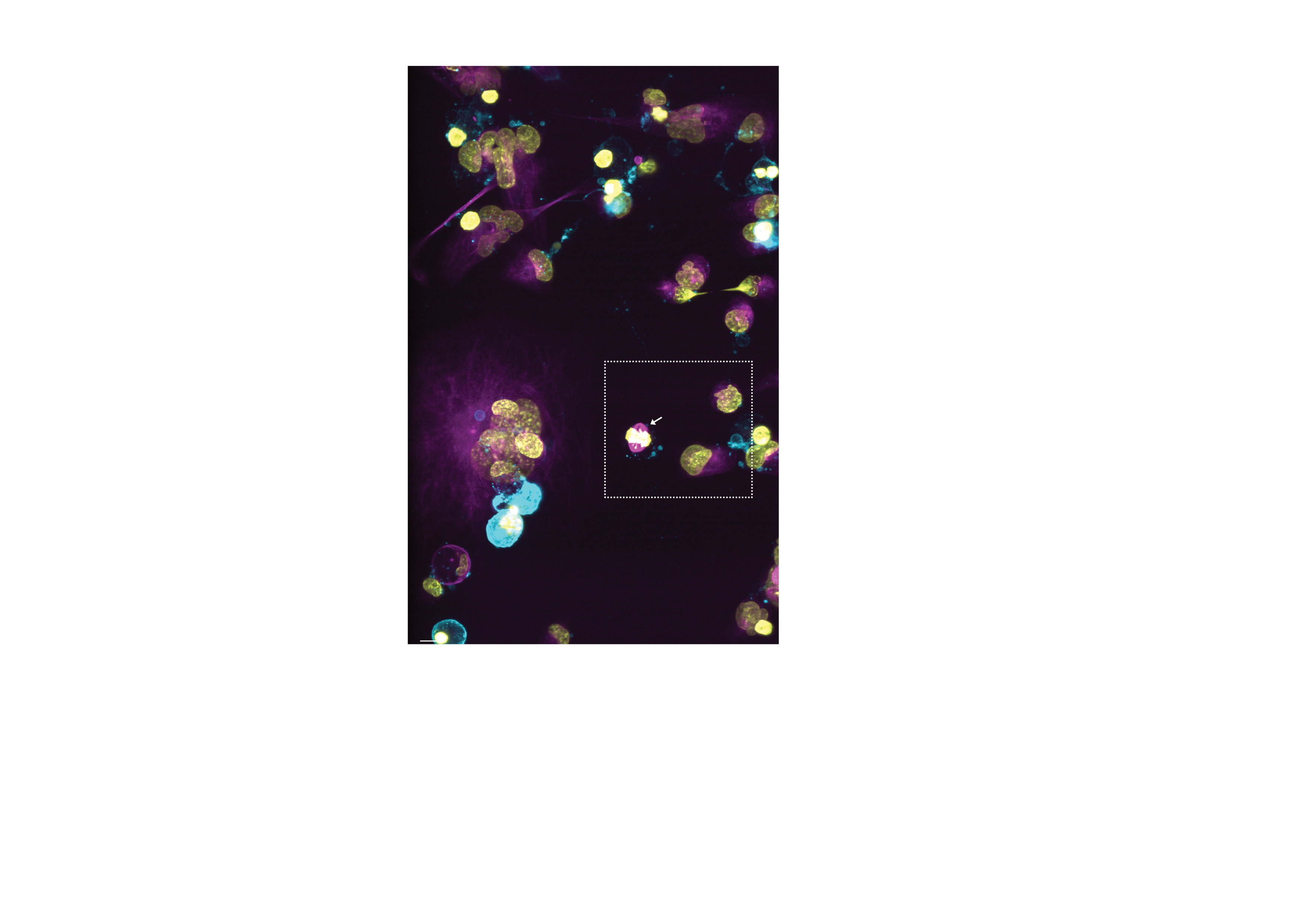

Supplement: Supplementary file 14 — Source data Fig. 7 [file 44318_2026_742_MOESM14_ESM.zip › FIgure 7/7E/MDA_arr.tiff]

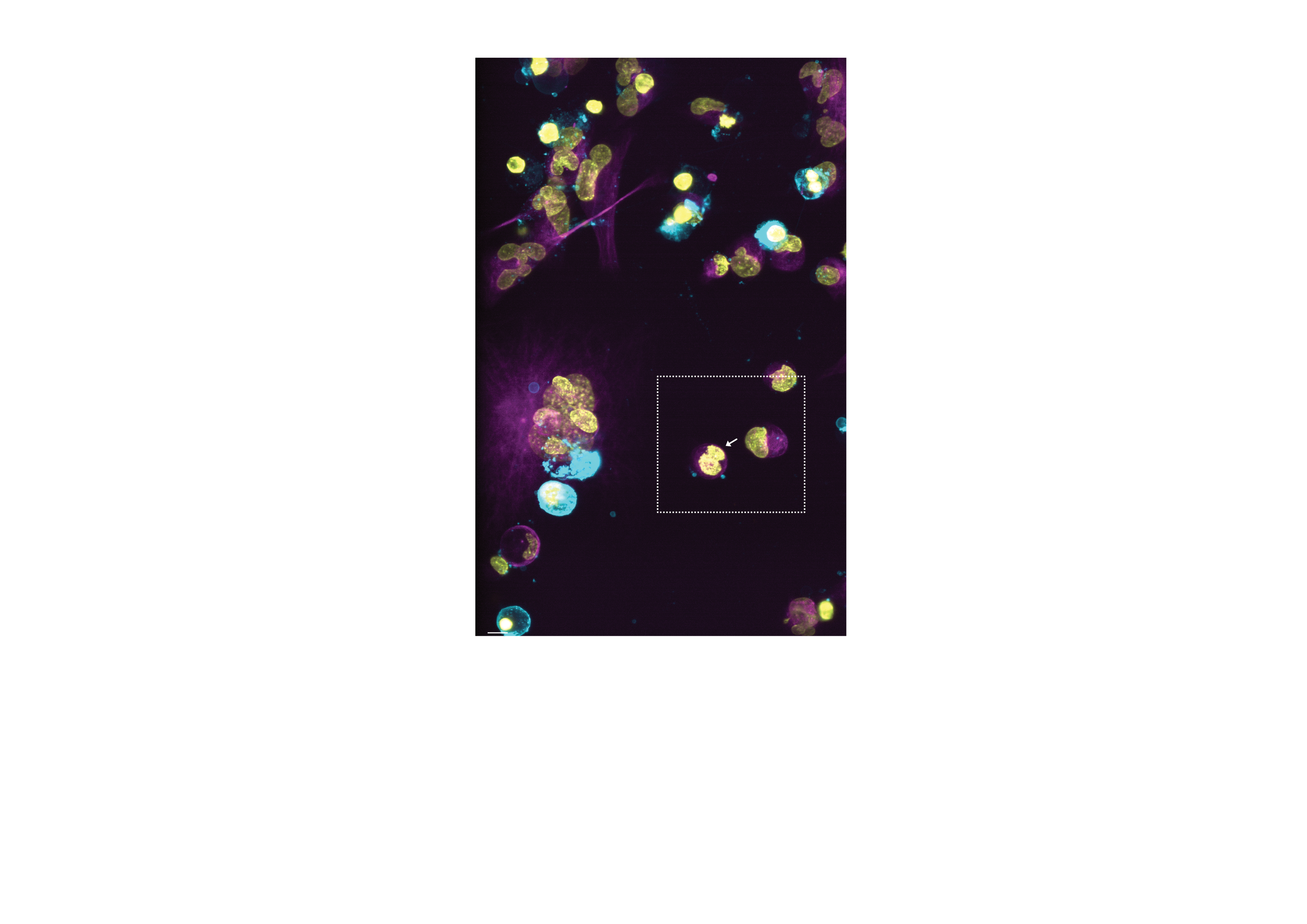

Supplement: Supplementary file 14 — Source data Fig. 7 [file 44318_2026_742_MOESM14_ESM.zip › FIgure 7/7E/MDA_post.tiff]

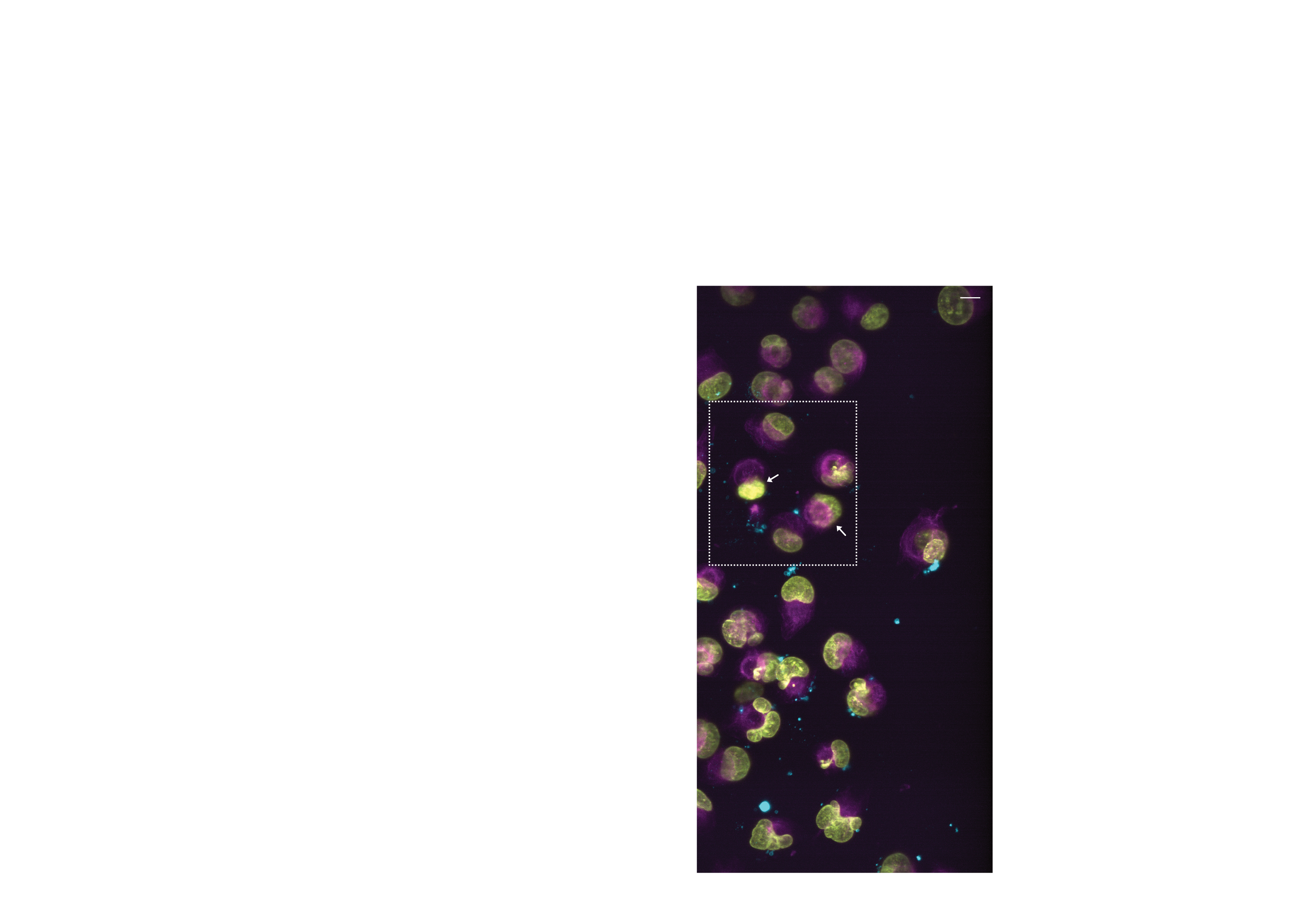

Supplement: Supplementary file 14 — Source data Fig. 7 [file 44318_2026_742_MOESM14_ESM.zip › FIgure 7/7E/Mia_post.tiff]

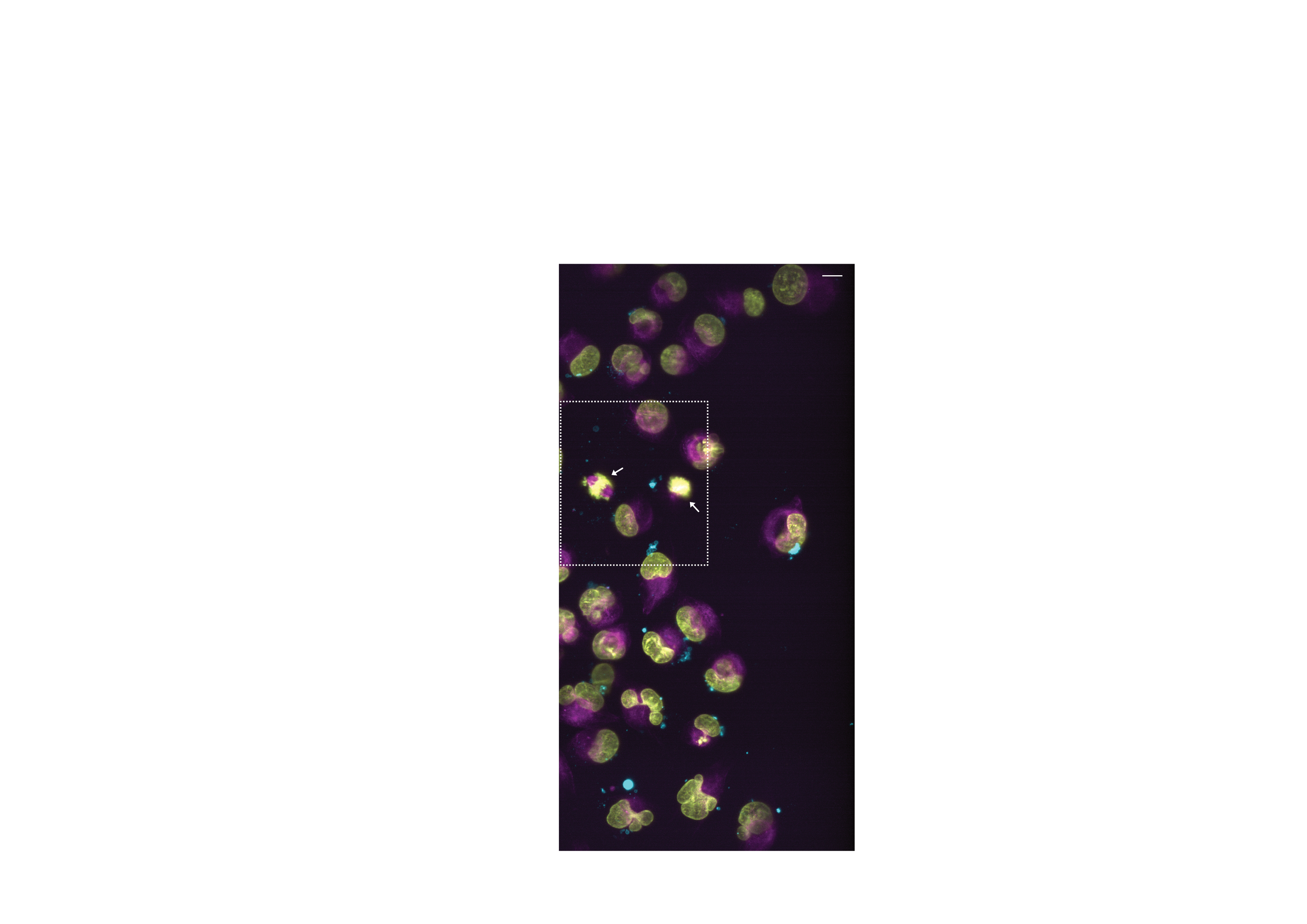

Supplement: Supplementary file 14 — Source data Fig. 7 [file 44318_2026_742_MOESM14_ESM.zip › FIgure 7/7E/Mia_arr.tiff]

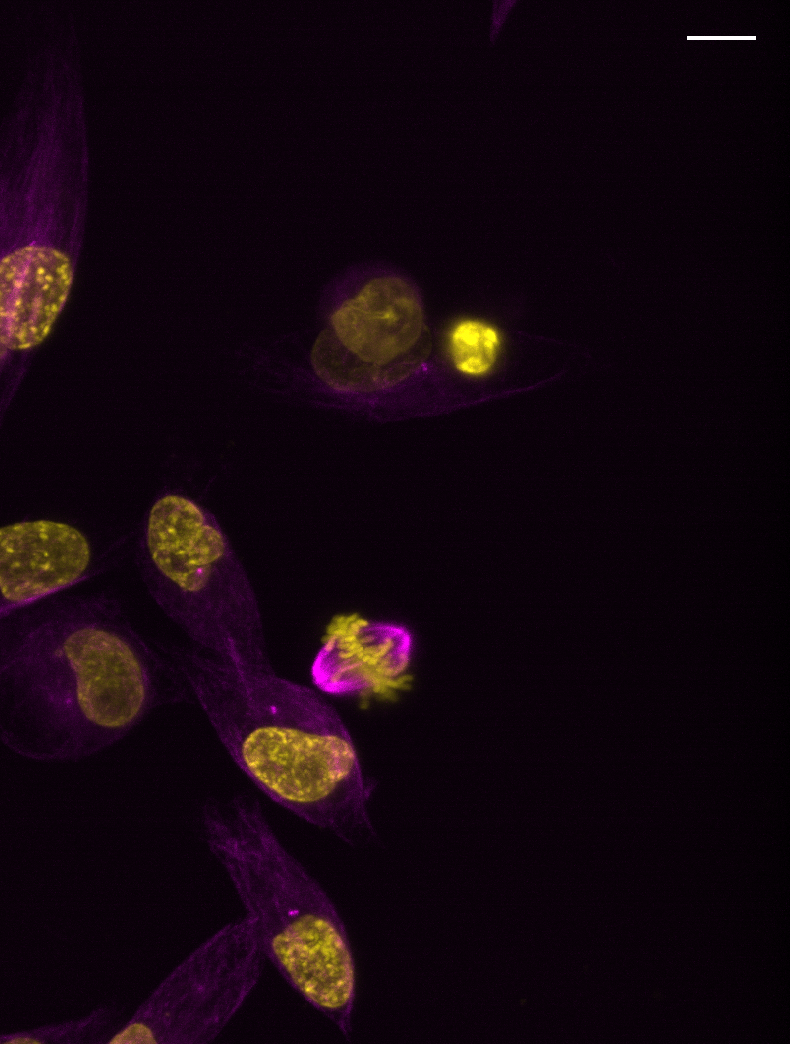

Supplement: Supplementary file 14 — Source data Fig. 7 [file 44318_2026_742_MOESM14_ESM.zip › FIgure 7/7A/MDA_unt_40.png]

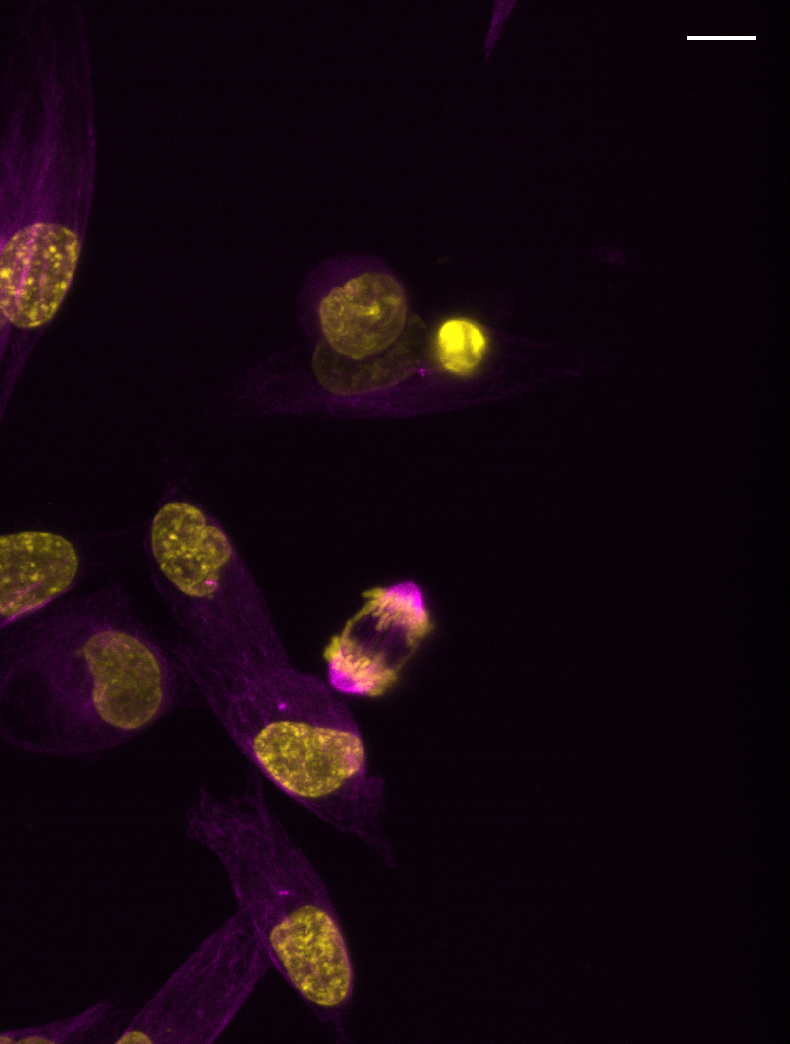

Supplement: Supplementary file 14 — Source data Fig. 7 [file 44318_2026_742_MOESM14_ESM.zip › FIgure 7/7A/MDA_unt_50.png]

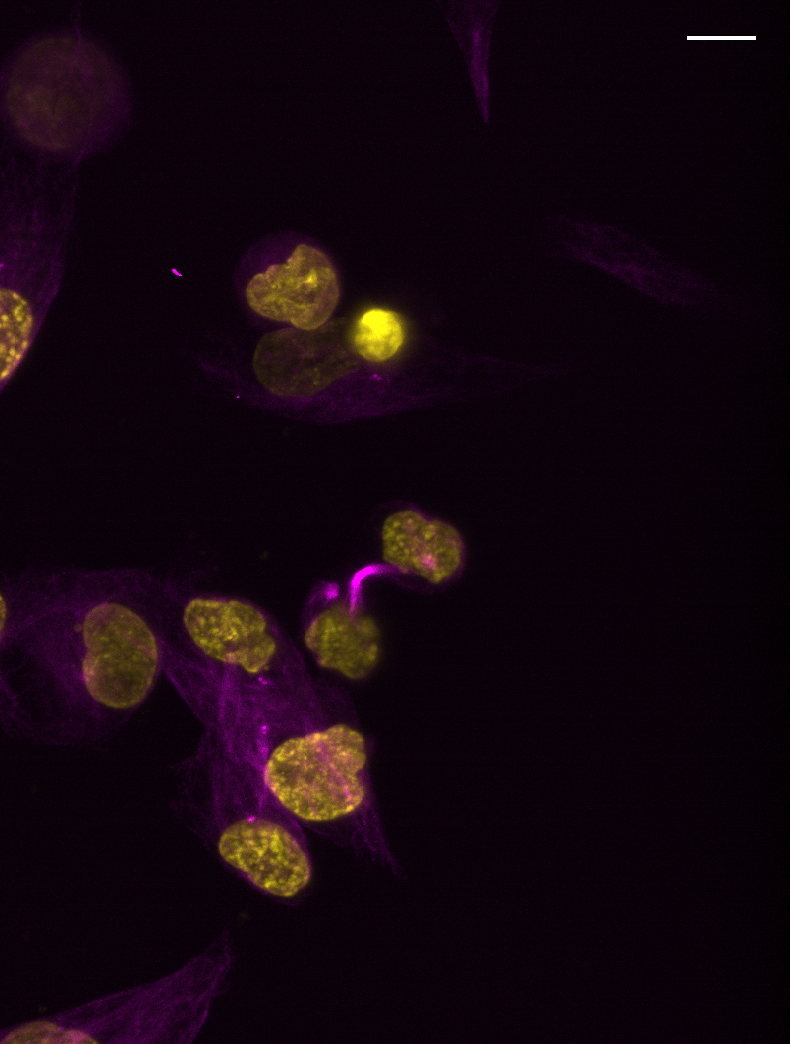

Supplement: Supplementary file 14 — Source data Fig. 7 [file 44318_2026_742_MOESM14_ESM.zip › FIgure 7/7A/MDA_unt_90.png]

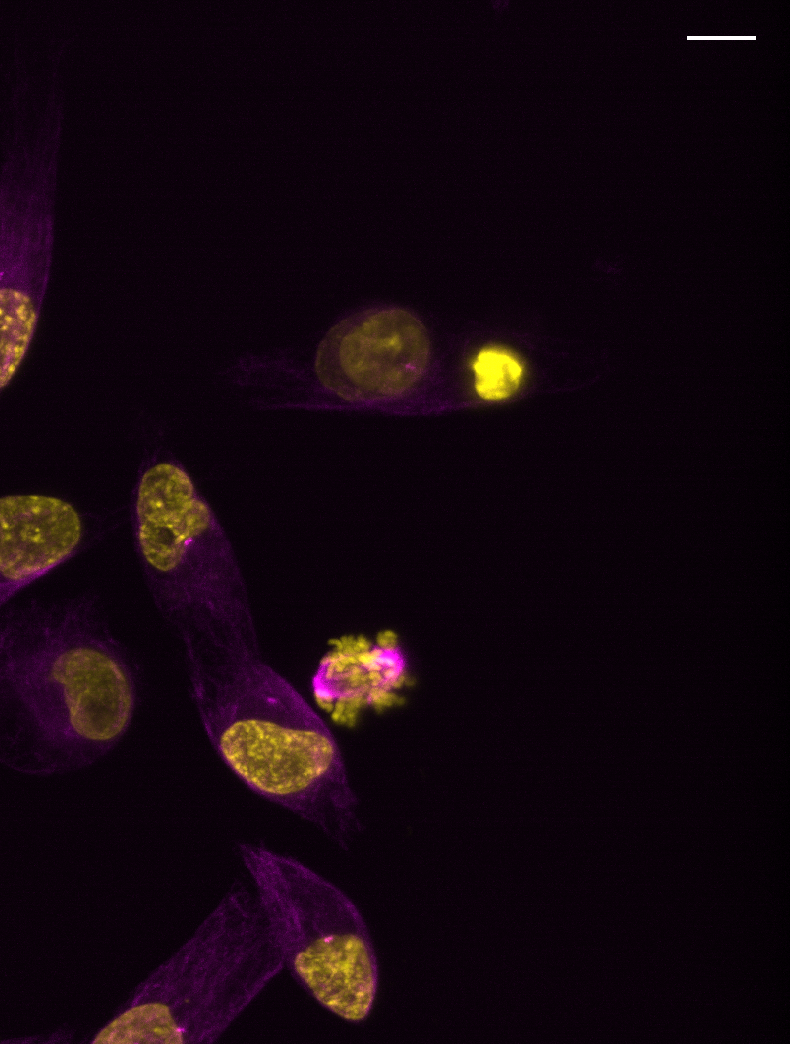

Supplement: Supplementary file 14 — Source data Fig. 7 [file 44318_2026_742_MOESM14_ESM.zip › FIgure 7/7A/MDA_unt_20.png]

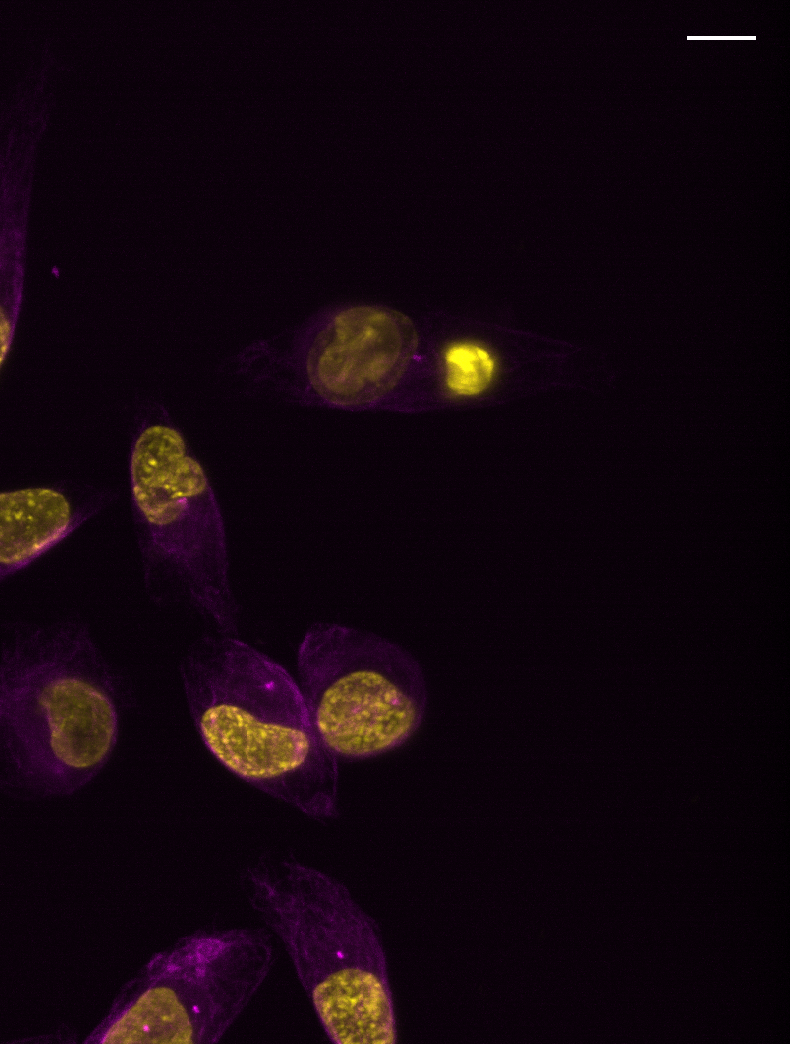

Supplement: Supplementary file 14 — Source data Fig. 7 [file 44318_2026_742_MOESM14_ESM.zip › FIgure 7/7A/MDA_unt_0.png]

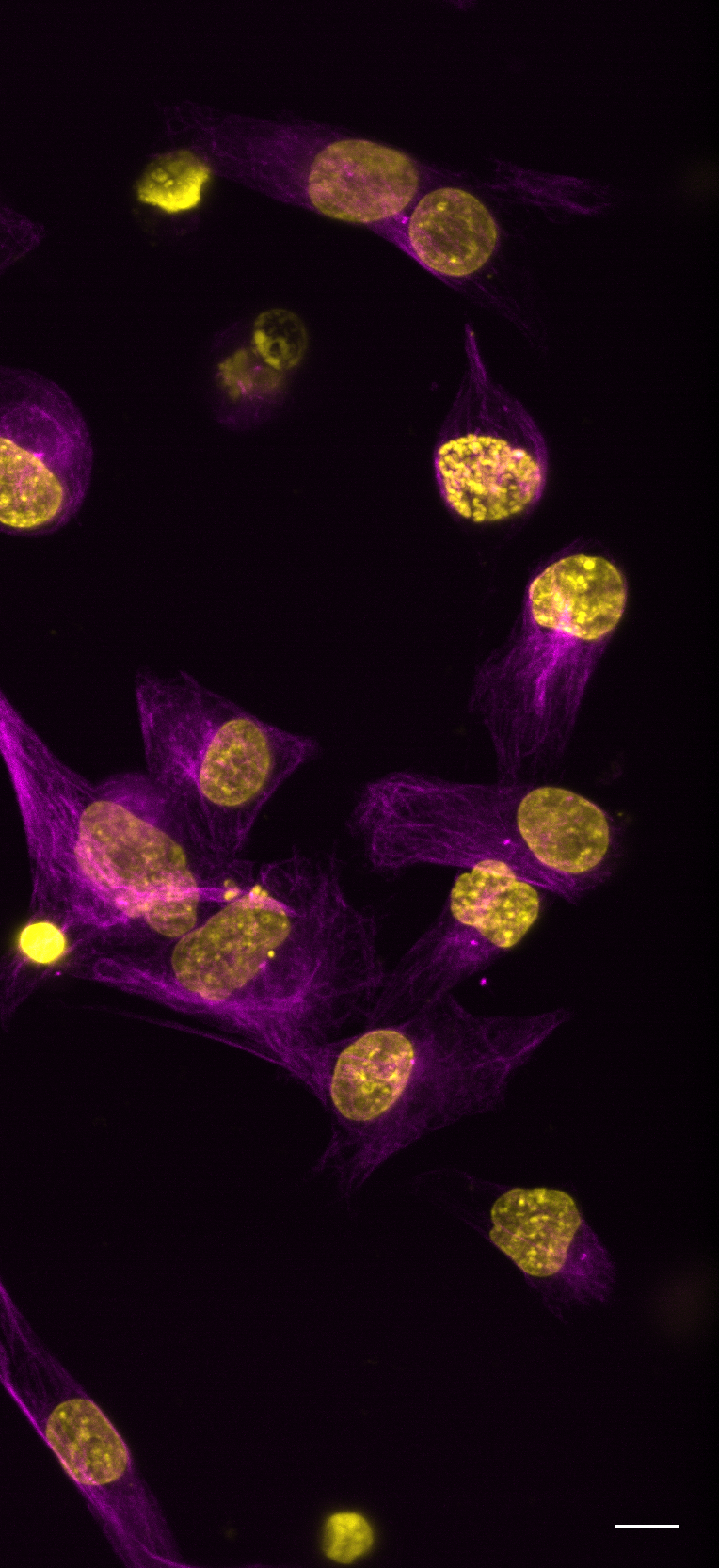

Supplement: Supplementary file 14 — Source data Fig. 7 [file 44318_2026_742_MOESM14_ESM.zip › FIgure 7/7A/MDA_tr_10.png]

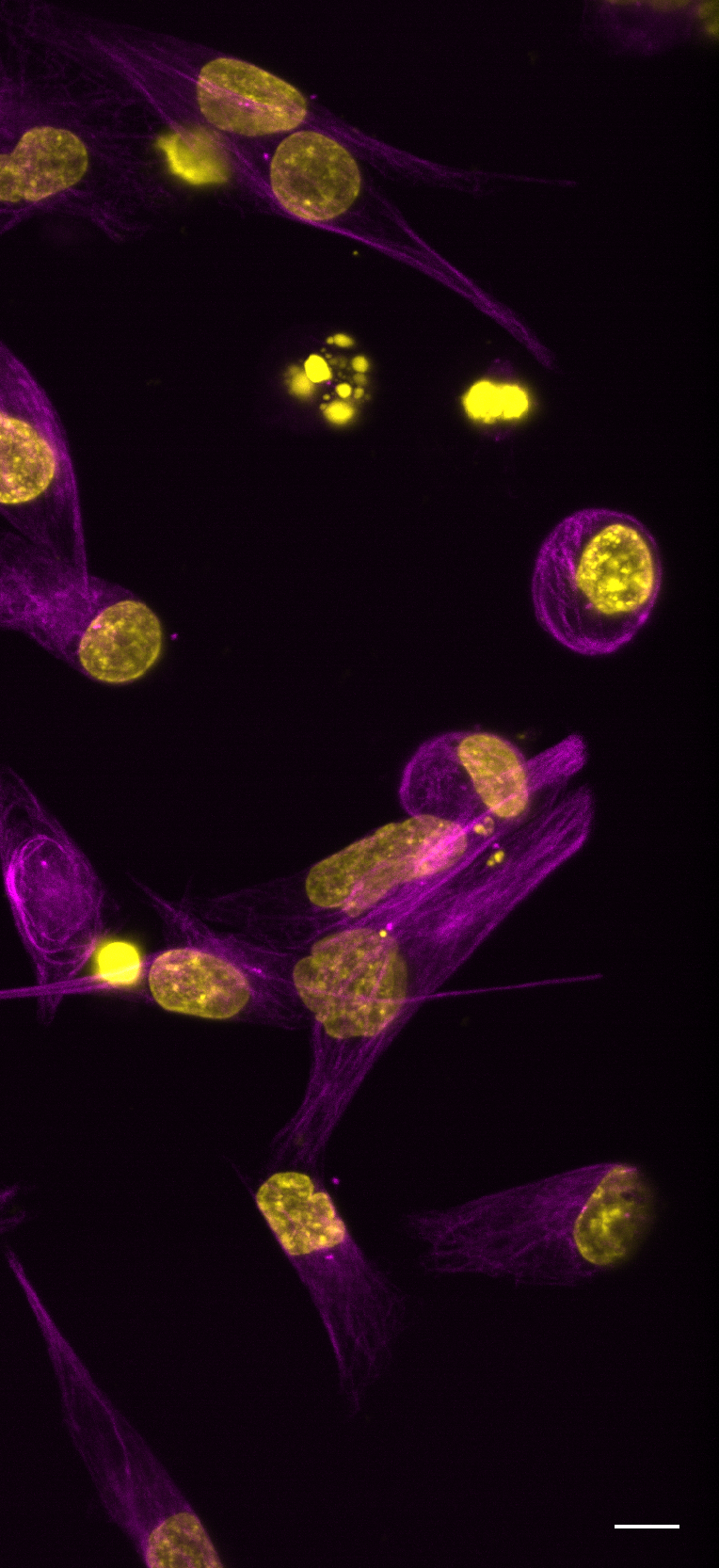

Supplement: Supplementary file 14 — Source data Fig. 7 [file 44318_2026_742_MOESM14_ESM.zip › FIgure 7/7A/MDA_tr_180.png]

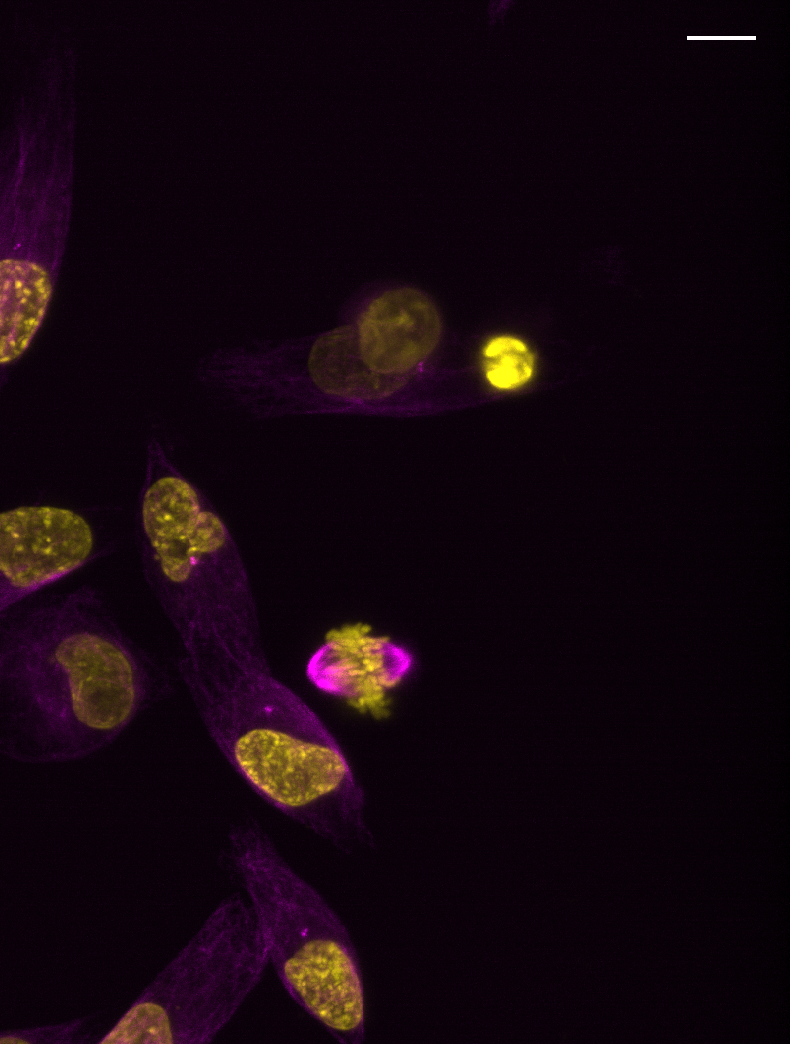

Supplement: Supplementary file 14 — Source data Fig. 7 [file 44318_2026_742_MOESM14_ESM.zip › FIgure 7/7A/MDA_unt_30.png]

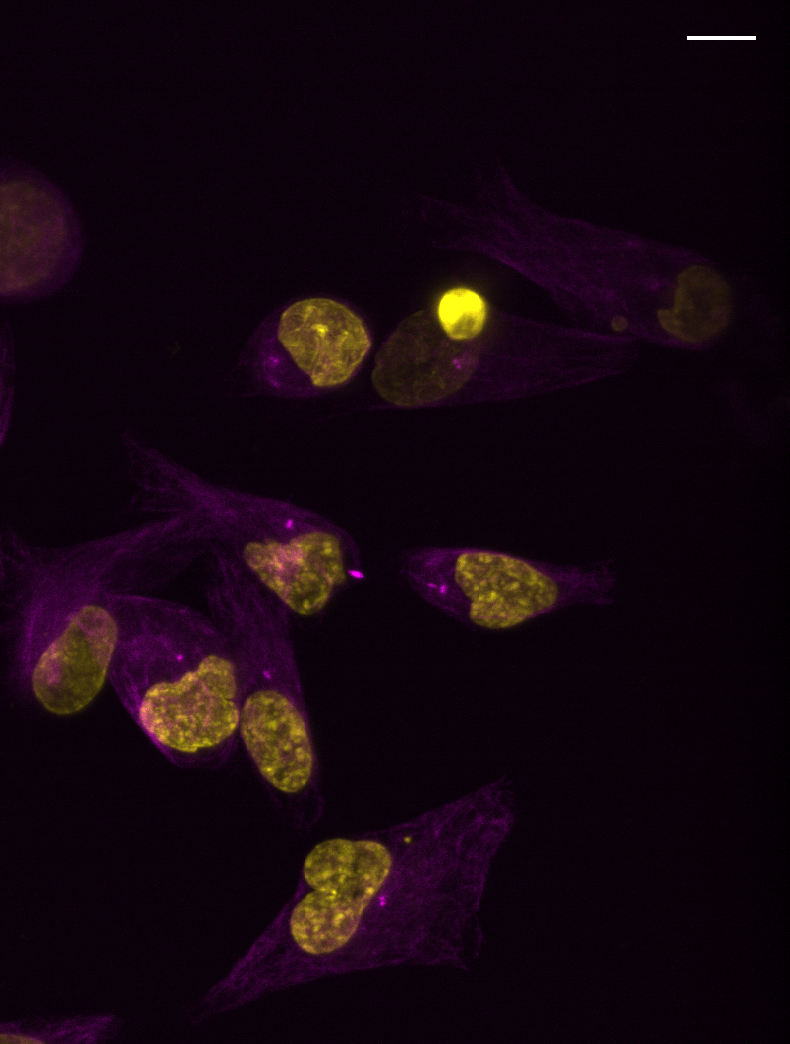

Supplement: Supplementary file 14 — Source data Fig. 7 [file 44318_2026_742_MOESM14_ESM.zip › FIgure 7/7A/MDA_unt_180.png]

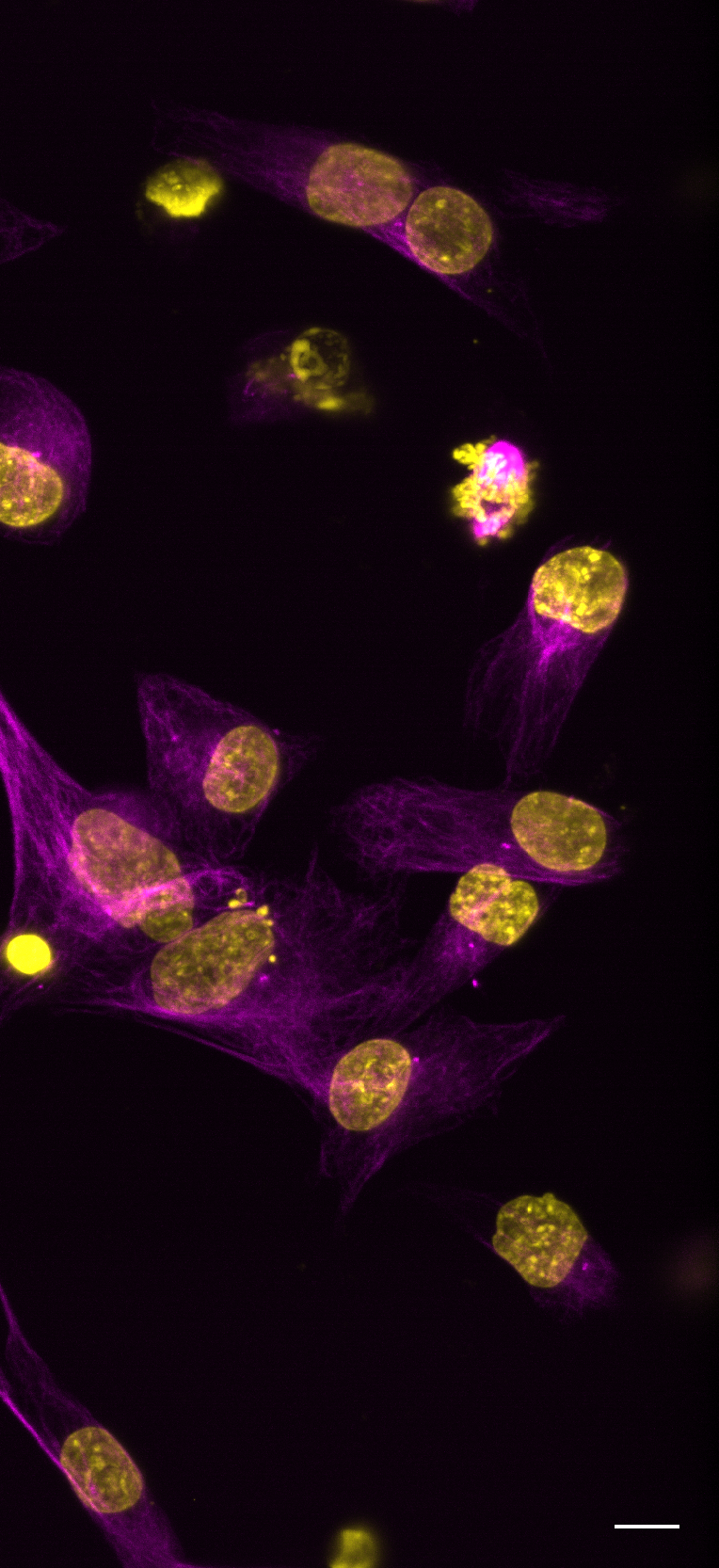

Supplement: Supplementary file 14 — Source data Fig. 7 [file 44318_2026_742_MOESM14_ESM.zip › FIgure 7/7A/MDA_tr_20.png]

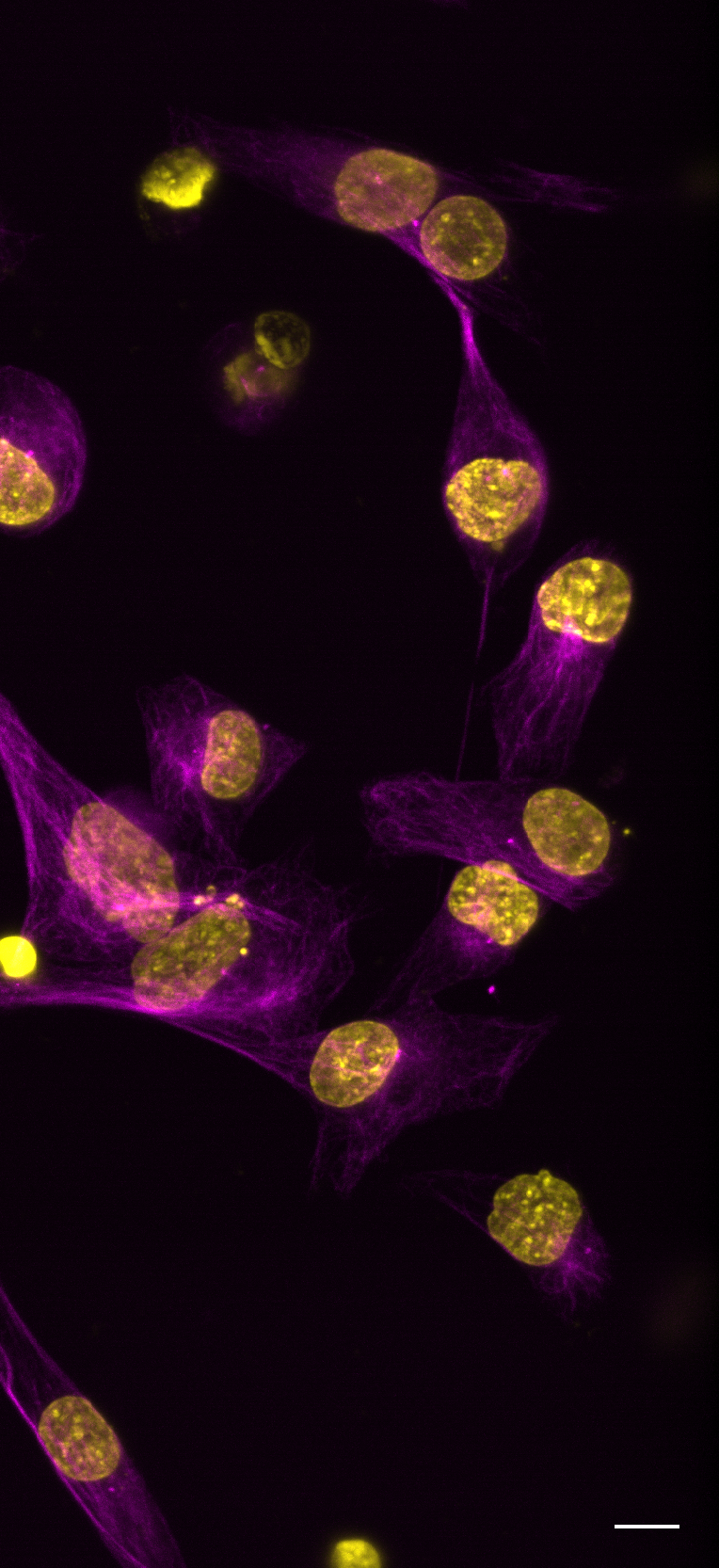

Supplement: Supplementary file 14 — Source data Fig. 7 [file 44318_2026_742_MOESM14_ESM.zip › FIgure 7/7A/MDA_tr_0.png]

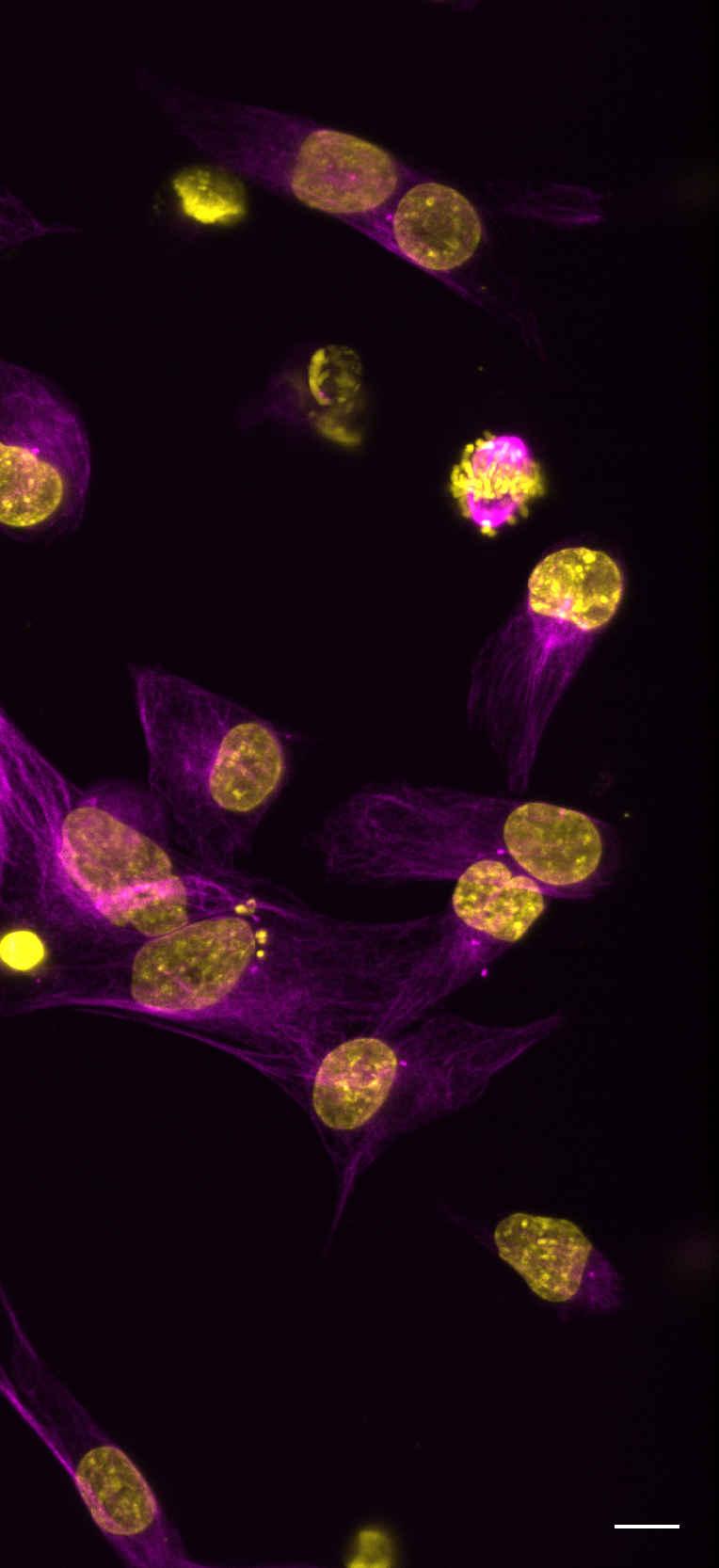

Supplement: Supplementary file 14 — Source data Fig. 7 [file 44318_2026_742_MOESM14_ESM.zip › FIgure 7/7A/MDA_tr_30.png]

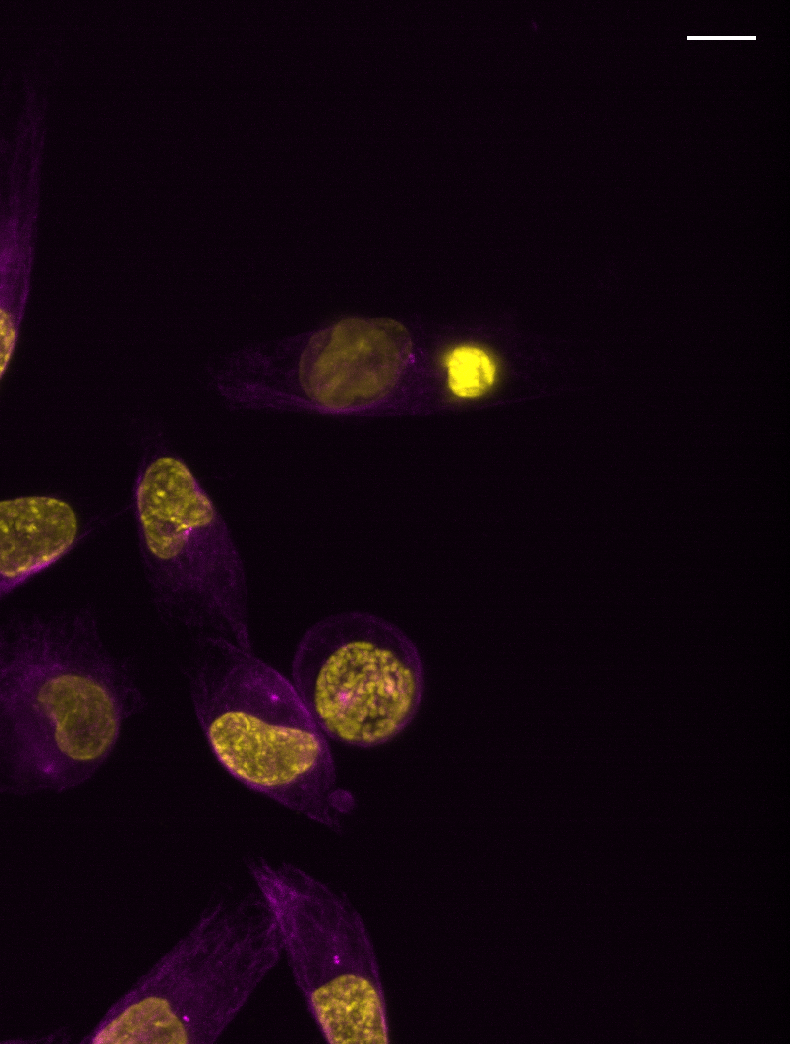

Supplement: Supplementary file 14 — Source data Fig. 7 [file 44318_2026_742_MOESM14_ESM.zip › FIgure 7/7A/MDA_unt_10.png]

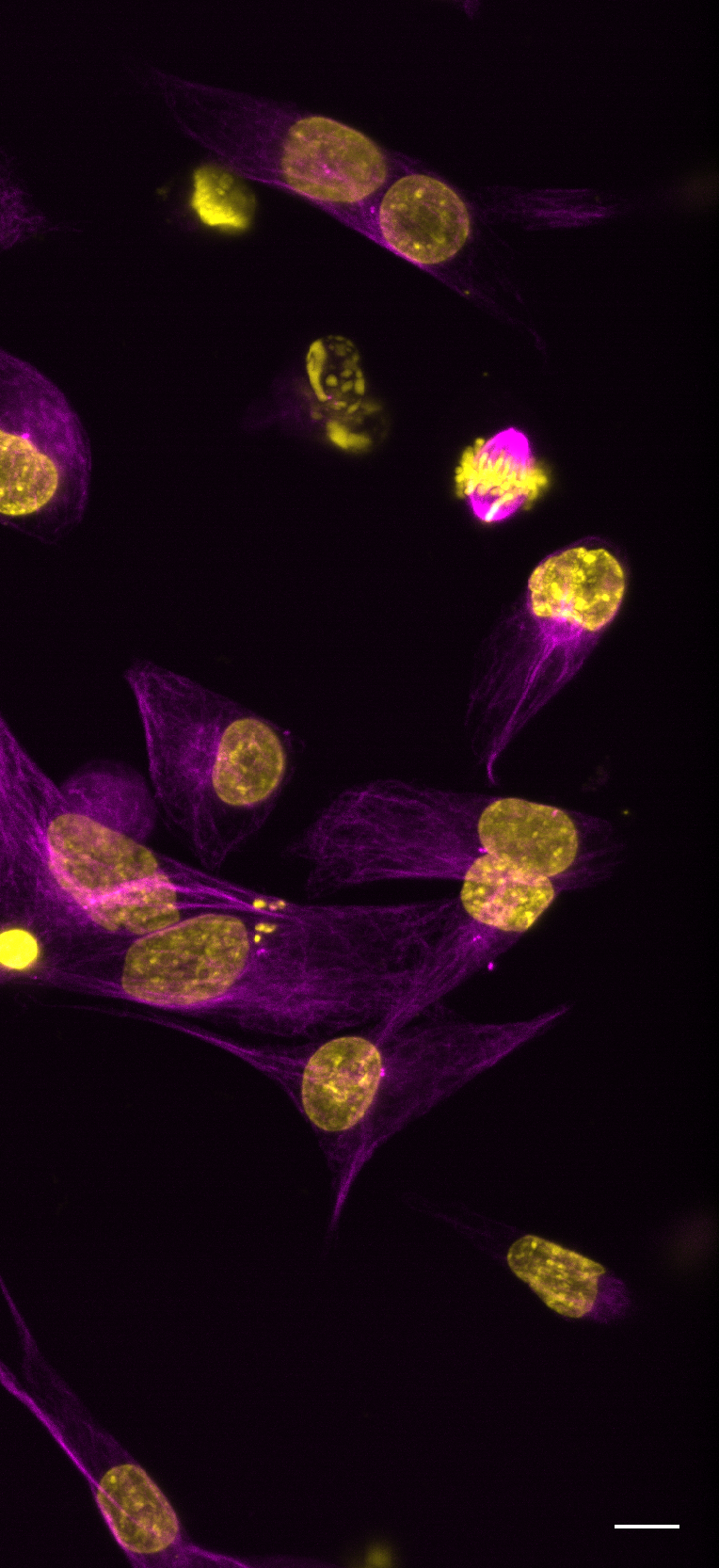

Supplement: Supplementary file 14 — Source data Fig. 7 [file 44318_2026_742_MOESM14_ESM.zip › FIgure 7/7A/MDA_tr_40.png]

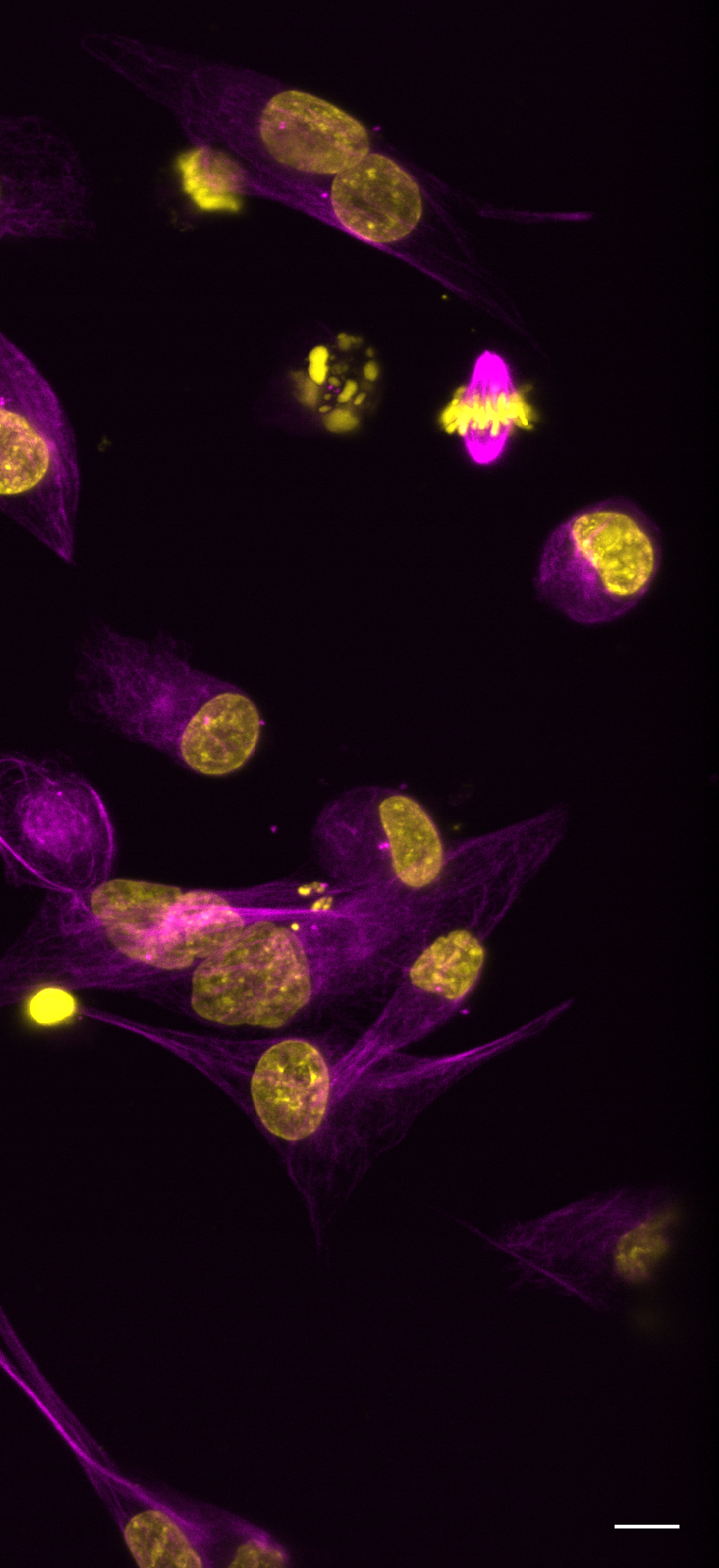

Supplement: Supplementary file 14 — Source data Fig. 7 [file 44318_2026_742_MOESM14_ESM.zip › FIgure 7/7A/MDA_tr_90.png]

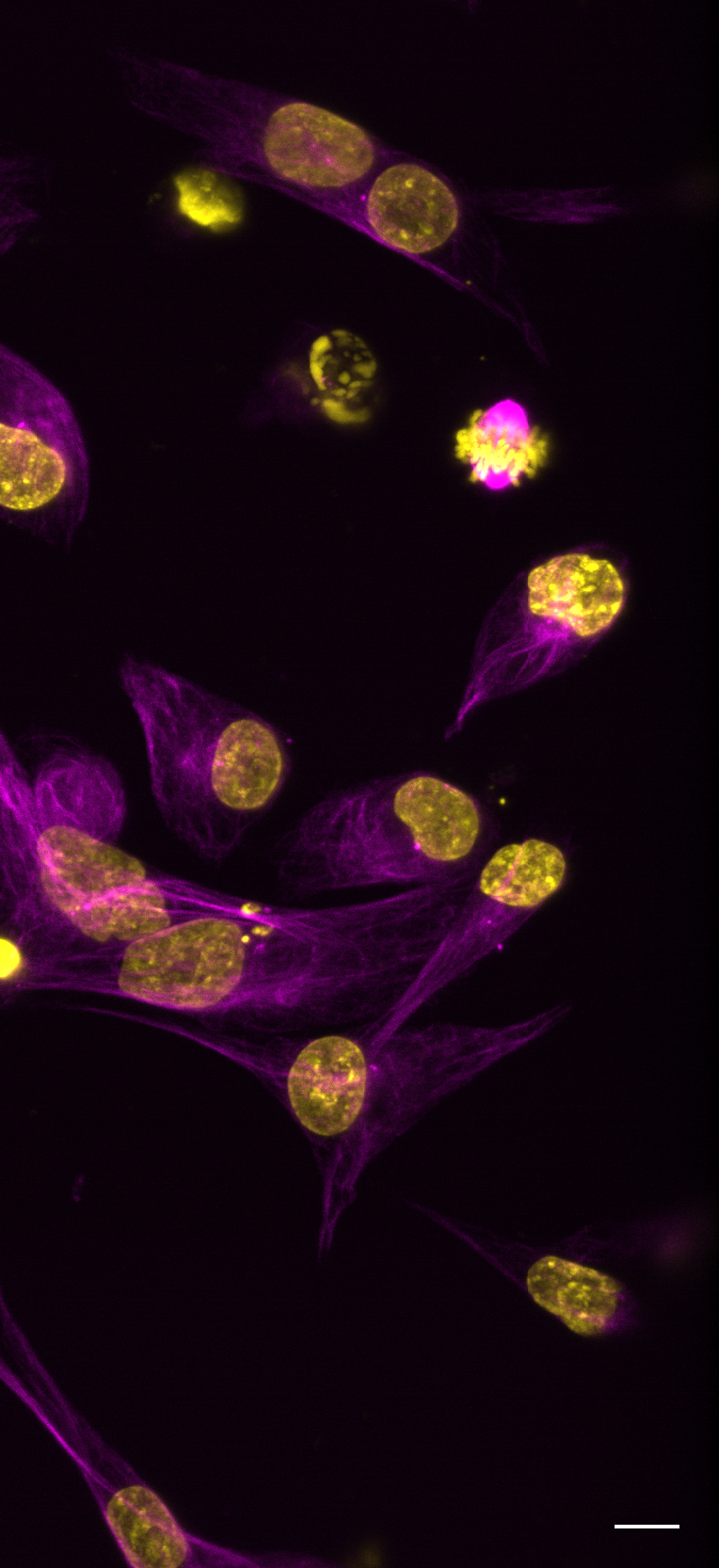

Supplement: Supplementary file 14 — Source data Fig. 7 [file 44318_2026_742_MOESM14_ESM.zip › FIgure 7/7A/MDA_tr_50.png]
