# Supplementary material for: Global analysis of cancer cell responses to USP9X inhibition
Source: EMBO J. 2026 Apr 7;45(9):3306–31. doi: 10.1038/s44318-026-00742-y (PMC13144739; doi:10.1038/s44318-026-00742-y)
Supplement: Supplementary file 16 — Expanded View Figures [file 44318_2026_742_MOESM16_ESM.pdf]

Expanded View Figures

Figure EV1. Time resolved ubiquitome profiling of WEHI-092-sensitive cell lines.

(**A**) Time resolved profiling of high-confidence USP9X substrates in SK-MEL-2 cells. Venn diagram showing the overlap of proteins with enhanced ubiquitination at 30 min (log2 >twofold, adjusted *P* value < 0.05 over untreated control) and decreased abundance at either 30 min, 360 min or 24 h WEHI-092 treatment (10 μM; log2 <−0.585, adjusted *P* value < 0.05 over untreated control) across four or five biological repeats per condition. Heatmap colours indicate fold change in protein ubiquitination (left) and protein expression (right) of proteins that showed significant induction (fold change log2 > 2, adjusted *P* value < 0.05 over untreated control) of at least one ubiquitination site at 30 min of WEHI-092 treatment (10 μM) and that were significantly downregulated (fold change log2 <−0.585, adjusted *P* value < 0.05 over untreated control) at 30 min/360 min/24 h. The data were averaged across four or five biological repeats per condition, and the data were matched based on gene ID level. Hierarchical clustering was performed on proteins (rows) with Euclidean distance as the similarity metric. (**B**) As in (**A**) but for UO-31 cells. Source data are available online for this figure.

▶

© The Author(s)

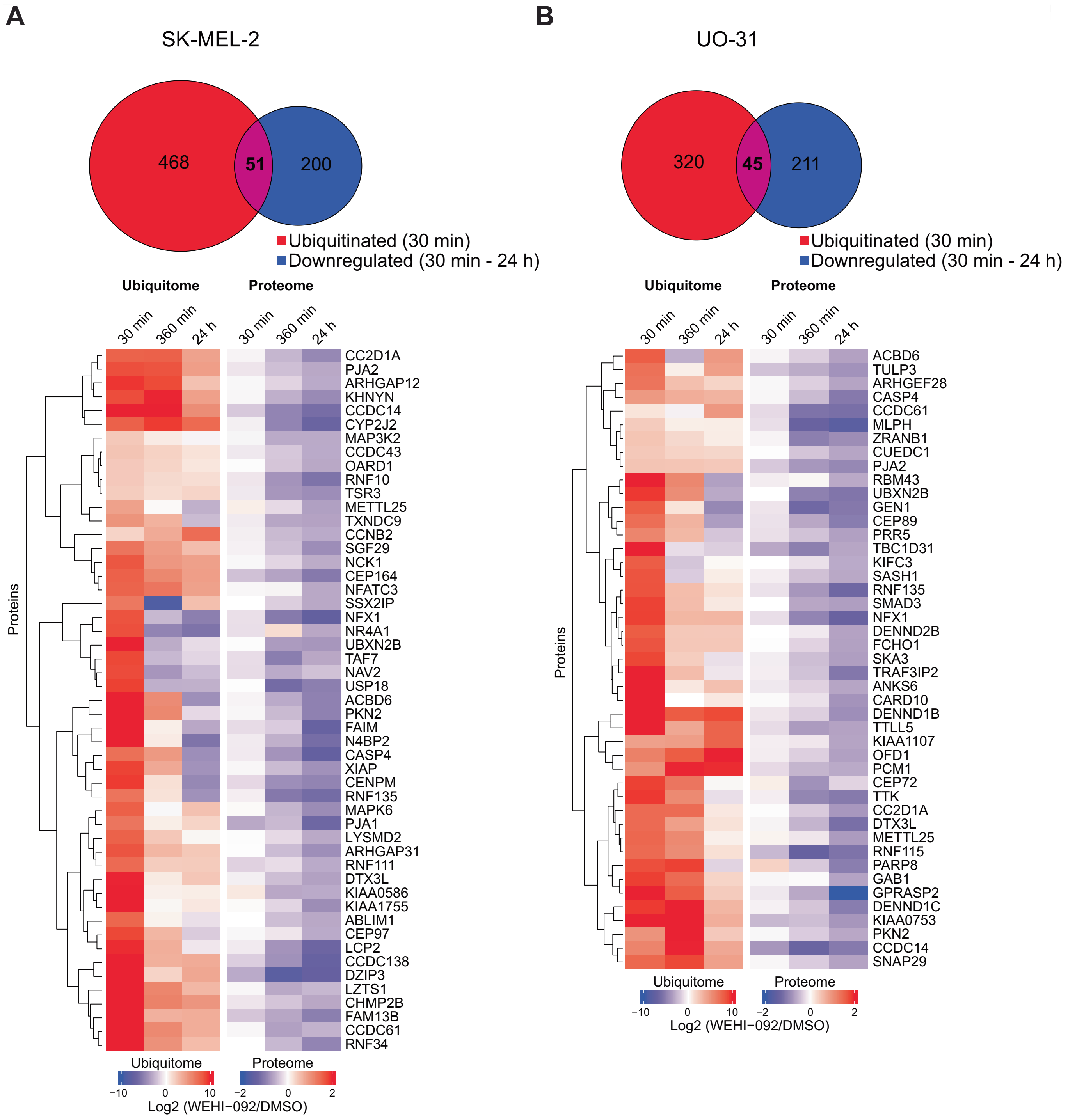
A
SK-MEL-2
468
51
200
Ubiquitinated (30 min)
Downregulated (30 min - 24 h)
Ubiquitome
Proteome
30 min
360 min
24 h
30 min
360 min
24 h
Proteins
CC2D1A
PJA2
ARHGAP12
KHNYN
CCDC14
CYP2J2
MAP3K2
CCDC43
OARD1
RNF10
TSR3
METTL25
TXNDC9
CCNB2
SGF29
NCK1
CEP164
NFATC3
SSX2IP
NFX1
NR4A1
UBXN2B
TAF7
NAV2
USP18
ACBD6
PKN2
FAIM
N4BP2
CASP4
XIAP
CENPM
RNF135
MAPK6
PJA1
LYSMD2
ARHGAP31
RNF111
DTX3L
KIAA0586
KIAA1755
ABLIM1
CEP97
LCP2
CCDC138
DZIP3
LZTS1
CHMP2B
FAM13B
CCDC61
RNF34
Ubiquitome
Proteome
−10
0
10
−2
0
2
Log2 (WEHI−092/DMSO)
B
UO-31
320
45
211
Ubiquitinated (30 min)
Downregulated (30 min - 24 h)
Ubiquitome
Proteome
30 min
360 min
24 h
30 min
360 min
24 h
Proteins
ACBD6
TULP3
ARHGEF28
CASP4
CCDC61
MLPH
ZRANB1
CUEDC1
PJA2
RBM43
UBXN2B
GEN1
CEP89
PRR5
TBC1D31
KIFC3
SASH1
RNF135
SMAD3
NFX1
DENND2B
FCHO1
SKA3
TRAF3IP2
ANKS6
CARD10
DENND1B
TTLL5
KIAA1107
OFD1
PCM1
CEP72
TTK
CC2D1A
DTX3L
METTL25
RNF115
PARP8
GAB1
GPRASP2
DENND1C
KIAA0753
PKN2
CCDC14
SNAP29
Ubiquitome
Proteome
−10
0
10
−2
0
2
Log2 (WEHI−092/DMSO)

 © The Author(s)
